# Metabolic alterations in plasma from patients with familial and idiopathic Parkinson’s disease

**DOI:** 10.18632/aging.103992

**Published:** 2020-09-09

**Authors:** Sokhna M.S. Yakhine-Diop, José A. Morales-García, Mireia Niso-Santano, Rosa A. González-Polo, Elisabet Uribe-Carretero, Guadalupe Martinez-Chacon, Sylvere Durand, Maria Chiara Maiuri, Ana Aiastui, Miren Zulaica, Javier Ruíz-Martínez, Adolfo López de Munain, Jordi Pérez-Tur, Ana Pérez-Castillo, Guido Kroemer, José M. Bravo-San Pedro, José M. Fuentes

**Affiliations:** 1 Departamento de Bioquímica y Biología Molecular y Genética, Facultad de Enfermería y Terapia Ocupacional, Universidad de Extremadura, Cáceres, Spain; 2 Centro de Investigación Biomédica en Red en Enfermedades Neurodegenerativas (CIBERNED), Madrid, Spain; 3 Instituto Universitario de Investigación Biosanitaria de Extremadura (INUBE), Cáceres, Spain; 4 Instituto de Investigaciones Biomédicas (CSIC-UAM) “Alberto Sols” (CSIC-UAM), Madrid, Spain; 5 Departamento de Biología Celular, Facultad de Medicina, Universidad Complutense de Madrid, Madrid, Spain; 6 Metabolomics and Cell Biology Platforms, Institut Gustave Roussy, Villejuif, France; 7 Centre de Recherche des Cordeliers, Equipe labellisée par la Ligue contre le cancer, Inserm U1138, Université de Paris, Sorbonne Université, Paris, France; 8 Cell Culture Platform, Biodonostia Health Research Institute, San Sebastián, Spain; 9 Neuroscience Area of Biodonostia Health Research Institute, Donostia University Hospital, San Sebastián, Spain; 10 Donostia University Hospital, Department of Neurology, OSAKIDETZA, Spain; 11 Ilundain Foundation, San Sebastian, Spain; 12 Department of Neurosciences, University of the Basque Country UPV-EHU, San Sebastián, Spain; 13 Instituto de Biomedicina de Valencia-CSIC, Unidad de Genética Molecular, Valencia, Spain; 14 Unidad Mixta de Genética y Neurología, Instituto de Investigación Sanitaria La Fe, Valencia, Spain; 15 Pôle de Biologie, Hôpital Européen Georges Pompidou, AP-HP, France; 16 Suzhou Institute for Systems Medicine, Chinese Academy of Medical Sciences, Suzhou, China; 17 Karolinska Institute, Department of Women's and Children's Health, Karolinska University Hospital, Stockholm, Sweden; 18 Departamento de Fisiología, Facultad de Medicina, Universidad Complutense de Madrid, Madrid, Spain

**Keywords:** bile acids, biomarkers, metabolism, Parkinson’s disease, purines

## Abstract

The research of new biomarkers for Parkinson’s disease is essential for accurate and precocious diagnosis, as well as for the discovery of new potential disease mechanisms and drug targets. The main objective of this work was to identify metabolic changes that might serve as biomarkers for the diagnosis of this neurodegenerative disorder. For this, we profiled the plasma metabolome from mice with neurotoxin-induced Parkinson’s disease as well as from patients with familial or sporadic Parkinson’s disease. By using mass spectrometry technology, we analyzed the complete metabolome from healthy volunteers compared to patients with idiopathic or familial (carrying the G2019S or R1441G mutations in the *LRRK2* gene) Parkinson’s disease, as well as, from mice treated with 6-hydroxydopamine to induce Parkinson disease. Both human and murine Parkinson was accompanied by an increase in plasma levels of unconjugated bile acids (cholic acid, deoxycholic acid and lithocholic acid) and purine base intermediary metabolites, in particular hypoxanthine. The comprehensive metabolomic analysis of plasma from Parkinsonian patients underscores the importance of bile acids and purine metabolism in the pathophysiology of this disease. Therefore, plasma measurements of certain metabolites related to these pathways might contribute to the diagnosis of Parkinson’s Disease.

## INTRODUCTION

Parkinson’s disease (PD) is the second most common neurodegenerative disease after Alzheimer’s disease, affecting 7-10 million people worldwide. PD results from a complex interaction between genetic and environmental factors, appearing mostly as idiopathic cases, with no identifiable cause. Nevertheless, the discovery of several gene mutations associated with PD onset point to a genetic origin of the disease [1]. Mutations in the PARK8/*LRRK2* gene are a common monogenic cause of PD. They are frequently found in early and late onset disease, in addition to this, familial or sporadic cases have been detected [2]. The substitution of glycine for serine in exon 41 of the protein kinase domain in *LRRK2* (G2019S mutation), is the most common mutation, as was estimated by the international LRRK2 consortium, representing 1% of sporadic and 4% of familial PD cases worldwide [3]. Also, different substitutions in the conserved GTPase domain, in exon 31 of *LRRK2* (R1441C, R1441G, and R1441H) have been identified as important genetic causes of familial PD [4].

Several therapies have been developed to relieve PD-related symptoms, improving the patient's quality of life [5]. However, there are no efficient therapies available to stop this neurodegenerative process, and it is necessary to discover the mechanisms that trigger the onset of neurodegeneration in order to develop etiological therapies [6].

Metabolomic analysis offers an interesting tool to identify biochemical networks linked with the pathogenesis of this poorly understood disease [7]. Most of metabolomic studies are based on the analysis of metabolites in cerebrospinal fluid (CSF) [8] and blood samples [9], although there are also some studies that have examined other biological samples such as urine [10, 11] or feces [12]. By using metabolomics, potential biomarkers have been discovered, including the biopyrrin, described as a new marker of idiopathic PD, after being found increased in the urine of these patients [11]. Moreover, dysregulated levels of polyamines, glutathione, kynurenine, pyruvate or cholesterol were all found in the plasma of PD patients compared to healthy individuals [13–18].

Cholesterol is a critical component of membrane bilayers and precursor of all steroid hormones and bile acids [19]. It plays key structural and functional roles in the general metabolism. Deregulations in cholesterol [9, 17, 18, 20–22] or in some of its products such as bile acids [23–25] have been linked to PD. However, studies on the association between serum cholesterol level and the risks of neurodegenerative diseases are currently under debate. High blood cholesterol, is a well-established risk factor for coronary disease and stroke [26, 27], but its role in PD remains controversial. Blood concentrations of cholesterol have been associated with a higher prevalence of PD [28, 29], and the use of cholesterol-lowering drugs, such as statins, have been associated with a decrease of PD [18]. However, another study failed to detect a significant association between serum cholesterol level and PD risk [30].

In addition, a correlation between changes in plasma levels of uric acid (UA) and the progression of neurodegenerative disorders has been described [14, 31]. Most notably, decreased UA levels in blood (hypouricemia) were found in PD patients [32]. In contrast, high levels of UA in blood (hyperuricemia) were shown to lower risks for the disease [33–35], and to protect against clinical progression in PD [36]. However, the mechanisms of this neuroprotective role of UA needs to be investigated further, as there are some contradictory studies that have shown an association between gout (medical condition associated with high levels of blood uric acid) and a higher [37] or lower [38] risk of PD.

Here, we report an extensive mass spectrometry metabolomic analysis of plasma samples from patients with genetic and idiopathic PD and from a mouse model in which PD was induced by 6-hydroxydopamine (6-OHDA).

## RESULTS

### Convergent metabolic changes observed in plasma from PD patients and a mouse model of PD

**Figure 1:**
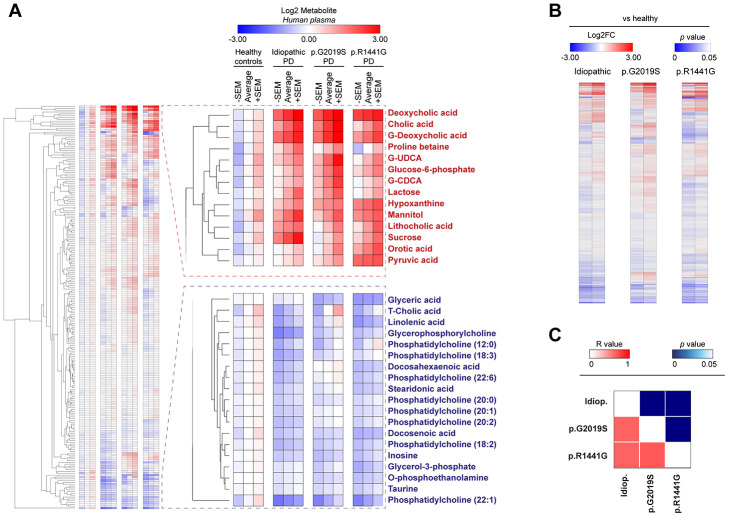
Heatmap clustered by Euclidean distance of changes in plasma metabolite concentrations depicted as Log2 in the control group (healthy), idiopathic and familial (carrying the p.G2019S or p.R1441G mutations in *LRRK2* gene) Parkinson’s disease (PD) patients (**A**) or Log2-fold change between each PD group respect to control group (**B**). Correlation matrix of all plasma metabolites changed in PD patients is shown by color codes (*p* values and Pearson’s coefficients of correlation ®) (**C**).

We performed mass spectrometric metabolomics of plasma from: *i*, patients with idiopathic PD; *ii*, PD patients with the p.G2019S or p.R1441G mutations of the *LRRK2* gene (Figure 1A and Supplementary Table 1); and *iii*, from a PD mouse model (Supplementary Table 2). These mice were treated with the neurotoxin 6-OHDA, that triggers a rapid degeneration of midbrain dopaminergic neurons in the *substantia nigra*.

Our data revealed that both PD cases exhibit important metabolic changes compared to matched controls, both in humans (Figure 1A) and in the mouse model (Supplementary Table 2). A deep analysis of the metabolites levels showed that PD patients groups (idiopathic and familial p.G2019S or p.R1441G) clustered together, underscoring that the differences observed in all metabolome (Figure 1B) or in specific biological pathways are very similar between these groups (Supplementary Figure 1A). When splitting the correlation analysis into subtypes of metabolites, we found that bile acids and purine pathways were significantly modulated in all PD patients (R value > 0.86 in all comparisons) (Figure 1C and Supplementary Figure 1), drawing our attention to these two pathways.

### Increased level of unconjugated bile acids in PD patients’ plasma

**Figure 2:**
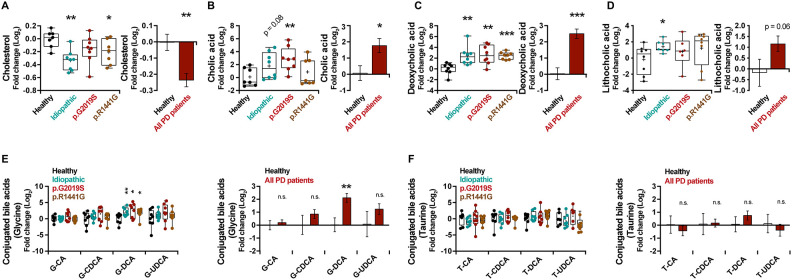
Box and whisker plots and graphs with average ± SEM of fold change (Log2) concentrations of cholesterol (**A**), CA (**B**), DCA (**C**), LCA (**D**), G-conjugated bile acids (**E**) and T-conjugated bile acids (**F**) in the control group (healthy), idiopathic and familial (harboring the p.G2019S or p.R1441G mutations in *LRRK2* gene) Parkinson’s disease (PD) patients. Abbreviations: CA, cholic acid; DCA, deoxycholic acid; G, glycine; LCA, lithocholic acid; PD, Parkinson’s disease; T, taurine.

Bile acid synthesis takes place in liver and consists in the oxidation of cholesterol (Supplementary Figure 2A). By analyzing the metabolite level, a decrease in the levels of cholesterol in plasma of PD patients (Figure 2A) (*p* = 0.006) and an important increase of the unconjugated bile acids, cholic acid (CA, *p* = 0.04) (Figure 2B), deoxycholic acid (DCA, *p* < 0.001) (Figure 2C) and lithocholic acid (LCA, *p* = 0.06) (Figure 2D) were observed. However, except for the glycine-conjugated DCA (G-DCA) metabolite, no general changes were found for other bile acids conjugated with glycine (G) (Figure 2E) or taurine (T) (Figure 2F).

**Figure 3:**
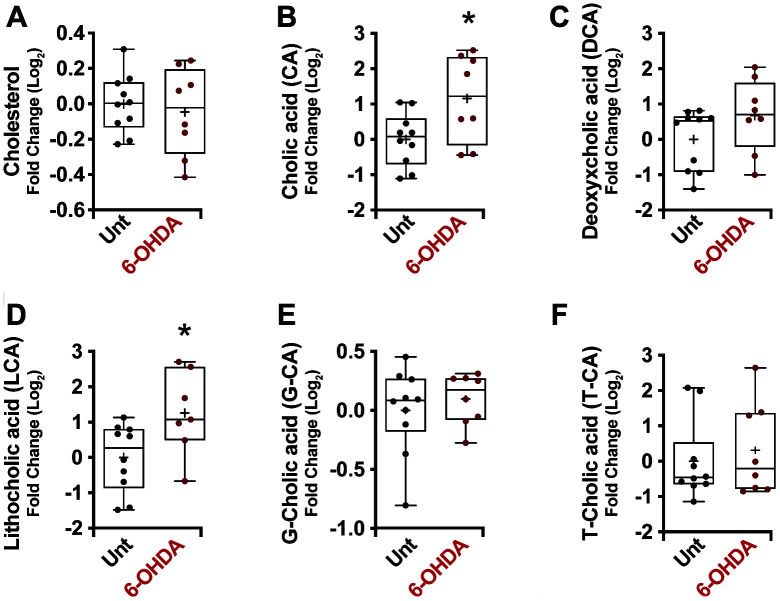
Box and whisker plots and graphs with average ± SEM of fold change (Log2) concentrations of cholesterol (**A**), CA (**B**), DCA (**C**), LCA (**D**), G-CA (**E**) and T-CA (**F**) in PD-mouse model. Abbreviations: 6-OHDA, 6-hydroxydopamine; CA, cholic acid; DCA, deoxycholic acid; G, glycine; LCA, lithocholic acid; PD, Parkinson’s disease; T, taurine; Unt, untreated.

No significant differences in cholesterol plasma levels (Figure 3A) were noticed in mice treated with 6-OHDA. Conversely, a general increase in unconjugated bile acid levels was observed in the plasma of these mice (CA, *p* = 0.02; DCA, *p* = 0.14; LCA, *p* = 0.02) (Figure 3B–3D), with no significant differences in conjugated bile acids (Figure 3E, 3F), consistent with the results obtained in human PD.

Additionally, bile acids have been described to boost the synthesis and storage of glycogen in the liver, which leads to an FXR-dependent decrease in blood glucose levels [39]. In our models, both in PD patients (all patients, Glucose, *p* value = 0.035) (Supplementary Table 1) and in the murine model (All patients, Glucose, *p* value = 0.048) (Supplementary Table 2), we can remark a slight but significant hypoglycemia that could be an indirect consequence of the increase of bile acids.

### The levels of uric acid and purine metabolic pathways are altered in plasma of all patients with PD

**Figure 4:**
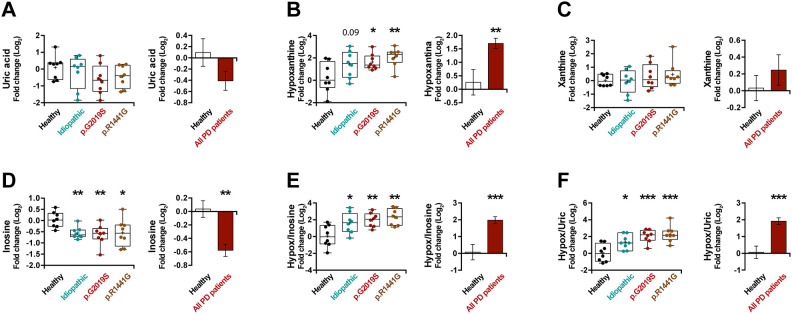
Box and whisker plots and graphs with average ± SEM of fold change (Log2) concentrations of uric acid (**A**), hypoxanthine (**B**), xanthine (**C**), inosine (**D**), hypoxanthine/inosine ratio (**E**) and hypoxanthine/uric acid ratio (**F**) in the control group (health), idiopathic and familial (carrying the p.G2019S or p.R1441G mutations in *LRRK2*) Parkinson’s disease (PD) patients.

UA has been reported as a risk factor in PD [35]. Accordingly, we observed lower UA levels in the plasma of PD patients (Figure 4A) (*p* = 0.13), however, this decrease was not spectacular. As UA is the end product of the metabolism of exogenous- and endogenous-derived purines (Supplementary Figure 3A), we decided to analyze in depth the rest of the metabolites of the purine pathway. Interestingly, the hypoxanthine levels in patients with PD are much higher than in healthy individuals (Figure 4B) (*p* < 0.01), but no major changes in xanthine levels (Figure 4C) were observed in these patients. In addition, analyzing the levels of a precursor of hypoxanthine, we can see a decrease in the levels of inosine (Figure 4D) (*p* < 0.001). On the same line, we noticed a very significant increase in PD patients in the ratios between hypoxanthine and its precursor (hypoxanthine/inosine ratio) (Figure 4E) (*p* < 0.001) or its product (hypoxanthine/UA ratio) (Figure 4F) (*p* < 0.001), confirming the accumulation of hypoxanthine. In summary, we observed an increase in hypoxanthine levels and a decrease in both its product and its precursor, suggesting a blockade of this metabolic cascade.

**Figure 5:**
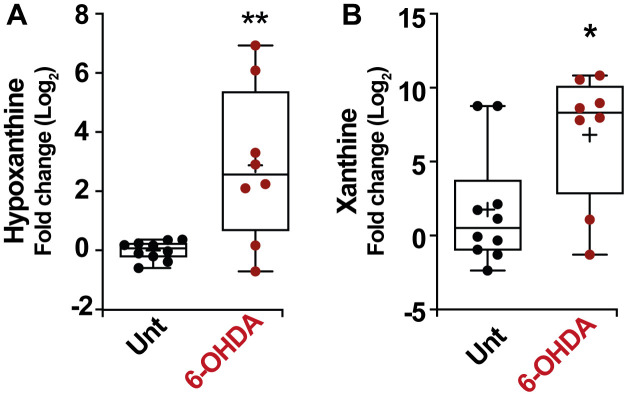
Box and whisker plots and graphs with average ± SEM of fold change (Log2) concentrations of hypoxanthine (**A**), xanthine (**B**), in PD-mouse model. Abbreviations: 6-OHDA, 6-hydroxydopamine; Unt, untreated.

As in the previous section, in order to verify these results, we also analyzed the modulation of the purine pathways in the murine PD model. Remarkably, an increase in the level of hypoxanthine was also observed in the serum of mice treated with 6-OHDA (Figure 5A) (*p* < 0.01) similarly to the result obtained in patients. In this model, the change in hypoxanthine level was accompanied by an increase in xanthine levels as well (Figure 5B) (*p* < 0.05). All these results together point to the robustness of the PD-associated increase in hypoxanthine.

## DISCUSSION

PD is a progressive disorder caused by degeneration of neurons in the *substantia nigra*, the area of the brain that controls movement. The first clinical symptoms of the disease appear when 80% of these nerve cells have disappeared, reducing the effectiveness of dopaminergic neurotransmission in this area [40]. Different theories related to environmental toxins, genetic factors, and accelerated aging have been discussed as possible causes of this disease, but most patients diagnosed with PD (around 80-85 percent) have what is called primary parkinsonism or idiopathic PD, meaning that the cause of the disease is unknown. Only a small percentage of patients present a genetic cause of PD origin. Five Mendelian genes causing familial PD have been identified: *PARK1*/*SNCA*, *PARK2/PARKN*, *PARK6/PINK1*, *PARK7*/DJ1, as well as the most prevalent one, *PARK8/LRRK2* [1, 41].

Our previous work has shown a basal autophagy impairment associated with an changes in intracellular protein acetylation levels in both genetic and idiopathic PD patients, leading to inefficient cellular responses to stress and increased susceptibility of cells to neurotoxins [41–43]. Indeed, several among the gene defects that cause human PD compromise the capacity of cells to destroy damaged mitochondria by autophagy (mitophagy), increasing cellular vulnerability to external and internal stress.

Extensive studies have been performed applying different “omic” technologies and particularly the study of the metabolome, the collection of small molecules (metabolites) contained in biological samples [7]. When performed in a high throughput manner [44], metabolomics can be considered as an emerging technology to explore PD-related biomarkers. Changes in metabolite concentrations have been detected in CSF samples from PD patients, as reported for oxidized glutathione, 3-hydroxykynurenine or homovanillate [8, 45–47]. However, the extraction of CSF carries some associated risks and is highly invasive [48]. Metabolomic studies performed on plasma samples, mainly focusing on idiopathic PD patients, identified new potential biomarkers in PD such as: polyamines [13], long-chain acylcarnitines [13], caffeine [49], tryptophan or bilirubin [50], glutathione or purine metabolism [14], cholesterol [9], kynurenine [15] or pyruvate [16]. However, in the past, only one study has been simultaneously performed on plasma from idiopathic and genetic (G2019S *LRRK2* mutation) PD patients. That study described that an aberration of the purine pathway in PD would account for UA changes [51]. Furthermore, one metabolomic study has been carried out in parallel on both humans idiopathic PD and mice treated with the Parkinsonian toxin MPTP. That study reported convergent changes in L-DOPA and DOPAC levels in plasma, as well as an increase in DRD3 expression on lymphocytes in human and murine PD [52]. In the present study, we explored for the first time the general metabolome of plasma from idiopathic or familial (G2019S or p.R1441G mutations in *LRRK2* gene) PD patients and also from a 6-OHDA-treated mice, providing evidence that bile acids and purine metabolic pathways play a role in the pathogenesis of PD.

We performed an in-depth analysis of the metabolic changes observed in plasma from all PD patients (idiopathic, p.G2019S or p.R1441G), observing that the subgroup of bile acids-pathways metabolites were convergently and significantly modulated in all PD patients and in 6-OHDA-treated mice. As previously shown [53], a significant decrease in the levels of cholesterol in the plasma from PD patients was observed. However, these alterations in cholesterol concentrations were relatively minor compared to the large increase in unconjugated bile acids in plasma from PD patients (Supplementary Figure 2B) or mice with PD (Supplementary Figure 2C). Considering that; *i,* there is still some uncertainty about the role of cholesterol in PD; *ii*, variations on bile acids occur in a murine model of prodromal PD in which mice received injections of α-SNCA fibrils [24], as well as in human idiopathic PD patients [54]; and *iii*, alterations of unconjugated bile acids in plasma occur in other neurodegenerative diseases, such as Alzheimer’s disease [55]; these results highlight the importance of bile acids as a potential early biomarker of PD.

Bile containing bile acids, cholesterol and other organic molecules is secreted by hepatocytes into canaliculi, flowing into bile ducts. In the liver, the synthesis of bile acids represents the majority of cholesterol breakdown of the body and plays a critical role in the digestion and absorption of lipids in the small intestine. Multiple waste products are removed from the organism by their secretion into bile and finally discarded in feces [56]. However, bile acids can be deconjugated and/or dehydroxylated by the intestinal microbiota, return to the liver via the portal circulation, where they undergo a new round of metabolic modification (such as reconjugation) and become detectable in systemic venous plasma [57]. The increase of unconjugated bile acids in plasma levels that we observed in PD may be explained by an increased bacterial degradation of conjugated bile acids and/or a less efficient removal of unconjugated bile acids from the peripheral circulation. Interestingly, recent results suggested a bidirectional communication between the gut and the brain [58, 59], and PD patients indeed exhibit a high prevalence of small intestinal bacterial overgrowth (SIBO) [60]. Therefore, a possible method to understand the influence of intestinal dysbiosis on these results would be through differential bacteria proportions, by performing a thorough analysis of fecal microbiota and measuring metabolite levels after the removal of the bacterial influence.

Another pathway that is significantly modified in both human and murine PD concerns purine metabolism. UA is one of the products of purine metabolism and many studies have shown a correlation between high levels of plasma UA and reduced prevalence of idiopathic PD [35], suggesting a protective role for this antioxidant. We observed a trend towards lower UA levels in plasma from PD patients, and also an important increase in hypoxanthine levels, an UA precursor (Supplementary Figure 3B). Similarly, significant changes were observed in this pathway in the PD mouse model, where hypoxanthine and xanthine plasma levels increased (Supplementary Figure 3C). The mechanisms leading to changes in UA associated to PD are not known, and the hypothesis that aberrations in the purine pathway occur in PD has to be examined in more detail. In 2009, Johansen *et al.* observed that differences in the UA precursors could be responsible, at least in part, for the final decrease in UA levels observed in PD patients [51]. It is interesting to note that in asymptomatic *LRRK2* p.G2019S carriers, hypoxanthine levels were significantly lower, but changes in UA levels were not significant. However, in advanced PD, the hypoxanthine levels were not significantly modified, and the levels of UA were notably reduced [51]. Thus, as the disease progresses, there appears to be a tendency to increase the levels of UA precursors when UA level decrease.

The strength of this study is the reproducibility of similar results in several different PD models: plasma from idiopathic, familial (p.G2019S and p.R1441G *LRRK2*) PD patients, and from a 6-OHDA-induced mouse model of PD, being the first complete metabolomic study carried out with these characteristics. The main limitation of our study lies in the number of individuals being tested. Increasing the number of patients studied would most likely strengthen the results. Moreover, it might be interesting to analyze asymptomatic *LRRK2* mutation carriers to understand whether the metabolomic shifts observed here occur before the disease becomes clinically apparent.

In summary, the present study identified robust changes in bile acids and purine pathways that may constitute biomarkers for both idiopathic and familial PD and potentially reflect pathogenesis-relevant metabolic alterations.

## MATERIALS AND METHODS

### PD patients:

Clinically established PD patients according to UK Bank criteria and healthy controls were divided into four groups (N=8/group): 1) healthy control individuals, 2) idiopathic PD patients, patients with 3) *LRRK2* p.G2019S mutation, and 4) *LRRK2* p.R1441G mutation. All the patient information (including age, gender and treatment received) is shown in Table 1. No interactions between gender or age and metabolic changes were observed (Table 2). All PD patients were treated with different antiparkinsonian medications (mainly levodopa/carbidopa or statins+levodopa/|carbidopa), but no significant differences in the metabolic changes between the two sub-groups within the PD patients were reported (Table 2). After informed consent approved by the Ethical Board of Hospital Donostia (ALM-LRRK2-2016-01) a blood sample was taken from antecubital vein after overnight fast and immediately processed to separate plasma and cells and stored at -80ºC until further experiments.

**Table 1:** Patient information.

**Sample ID code**	**Age (years, at plasma collection)**	**Gender**	**Health Condition**	**Treatment**
FH17-07	64	Female	Healthy individual	Untreated
FH17-08	77	Male	Healthy individual	Untreated
FH17-09	68	Female	Healthy individual	Untreated
FH17-10	65	Female	Healthy individual	Untreated
FH17-11	72	Female	Healthy individual	Untreated
FH17-12	72	Female	Healthy individual	Untreated
FH17-16	76	Female	Healthy individual	Untreated
FH17-18	68	Female	Healthy individual	Untreated
FH16-24	77	Male	Idiopathic PD patient	Statins
FH16-25	65	Male	Idiopathic PD patient	Statins + Levodopa
FH16-26	63	Male	Idiopathic PD patient	Levodopa
FH16-31	81	Female	Idiopathic PD patient	Levodopa
FH16-32	85	Male	Idiopathic PD patient	Levodopa
FH16-34	71	Male	Idiopathic PD patient	Levodopa
FH16-35	71	Male	Idiopathic PD patient	Statins
FH16-38	48	Male	Idiopathic PD patient	Levodopa
FH13-11	84	Female	p.G2019S PD patient	Levodopa
FH13-13	73	Female	p.G2019S PD patient	Levodopa
FH16-39	81	Male	p.G2019S PD patient	Levodopa
FH16-43	77	Female	p.G2019S PD patient	Statins + Levodopa
FH16-44	79	Female	p.G2019S PD patient	Statins + Levodopa
FH17-03	78	Female	p.G2019S PD patient	Levodopa
FH17-01	67	Female	p.G2019S PD patient	Statins + Levodopa
FH16-45	73	Female	p.G2019S PD patient	Levodopa
FH16-27	72	Male	p.R1441G PD patient	Levodopa
FH16-28	68	Male	p.R1441G PD patient	Levodopa
FH17-15	67	Male	p.R1441G PD patient	Statins + Levodopa
FH13-11	88	Female	p.R1441G PD patient	Statins + Levodopa
FH13-13	77	Female	p.R1441G PD patient	Untreated
FH09-78	67	Male	p.R1441G PD patient	Statins + Levodopa
FH10-11	54	Male	p.R1441G PD patient	Levodopa
FH10-12	59	Female	p.R1441G PD patient	Levodopa

Abbreviations: PD, Parkinson’s disease.

**Table 2:** Effects of gender, age and treatment received on the results obtained.

**Analysis by gender**						
**Metabolite**	**Mean of healthy female**	**Mean of PD female**	***p* value**	**Mean of PD male**	**Mean of PD female**	***p* value**
**Uric acid**	-0,0788	-0,8681	*	-0,0805	-0,8681	*
**Hypoxanthine**	0,2608	1,7860	*	1,6430	1,7860	n.s.
**Inosine**	0,1051	-0,5730	**	-0,5820	-0,5730	n.s.
**Xanthine**	-0,0384	0,4768	n.s.	0,0812	0,4768	n.s.
**Cholesterol**	0,0027	-0,2105	*	-0,2738	-0,2105	n.s.
**CA**	-0,2193	2,0500	*	1,7480	2,0500	n.s.
**LCA**	-0,4863	1,0380	n.s.	1,2440	1,0380	n.s.
**DCA**	-0,1294	2,4460	**	2,5520	2,4460	n.s.
**G-CA**	-0,0499	0,2865	n.s.	0,1431	0,2865	n.s.
**G-DCA**	-0,1316	2,0740	*	2,1380	2,0740	n.s.
**T-CA**	-0,0576	-0,4365	n.s.	-0,4339	-0,4365	n.s.
**T-DCA**	-0,1311	0,4159	n.s.	0,9882	0,4159	n.s.

**Analysis by age (Correlations between age and metabolic changes)**										
**Metabolite**	**All individuals**		**PD patients**		**Idiopathic patients**		**p.G2019S patients**		**p.R1441G patients**	
	**R squared**	***p* value (two-tailed)**	**R squared**	***p* value (two-tailed)**	**R squared**	***p* value (two-tailed)**	**R squared**	***p* value (two-tailed)**	**R squared**	***p* value (two-tailed)**
**Uric acid**	0,0004	n.s.	0,0019	n.s.	0,3176	n.s.	0,0461	n.s.	0,6650	*
**Hypoxanthine**	0,0516	n.s.	0,0963	n.s.	0,3447	n.s.	0,1044	n.s.	0,1376	n.s.
**Inosine**	0,0000	n.s.	0,0020	n.s.	0,0171	n.s.	0,0003	n.s.	0,0070	n.s.
**Xanthine**	0,0148	n.s.	0,0226	n.s.	0,1705	n.s.	0,0184	n.s.	0,0348	n.s.
**Cholesterol**	0,0045	n.s.	0,0086	n.s.	0,1640	n.s.	0,0053	n.s.	0,0457	n.s.
**CA**	0,0428	n.s.	0,0321	n.s.	0,0459	n.s.	0,2118	n.s.	0,0876	n.s.
**LCA**	0,0137	n.s.	0,0218	n.s.	0,1566	n.s.	0,0259	n.s.	0,0010	n.s.
**DCA**	0,0305	n.s.	0,0163	n.s.	0,0007	n.s.	0,0036	n.s.	0,1576	n.s.
**G-CA**	0,0289	n.s.	0,0220	n.s.	0,0231	n.s.	0,0291	n.s.	0,0920	n.s.
**G-DCA**	0,0527	n.s.	0,0516	n.s.	0,1204	n.s.	0,1568	n.s.	0,0250	n.s.
**T-CA**	0,0004	n.s.	0,0047	n.s.	0,0002	n.s.	0,0252	n.s.	0,3312	n.s.
**T-DCA**	0,0080	n.s.	0,0368	n.s.	0,0852	n.s.	0,0125	n.s.	0,5425	*

**Analysis by treatments**									
**Metabolite**	**Mean of healthy (Untreated)**	**Mean of PD patients (Levodopa)**	***p* value**	**Mean of healthy (Untreated)**	**Mean of PD patients (Levodopa+ Statins)**	***p* value**	**Mean of PD patients (Levodopa)**	**Mean of PD patients (Levodopa+ Statins)**	***p* value**
**Uric acid**	0,0962	-0,3709	n.s.	0,0962	-0,1443	n.s.	-0,3709	-0,1443	n.s.
**Hypoxanthine**	0,2592	1,7140	**	0,2592	2,3000	**	1,7140	2,3000	n.s.
**Inosine**	0,0368	-0,6213	**	0,0368	-0,4592	n.s.	-0,6213	-0,4592	n.s.
**Xanthine**	0,0317	0,2150	n.s.	0,0317	0,7492	*	0,2150	0,7492	n.s.
**Cholesterol**	-0,0165	-0,2613	**	-0,0165	-0,1298	n.s.	-0,2613	-0,1298	n.s.
**CA**	0,0898	1,6490	n.s.	0,0898	2,0230	*	1,6490	2,0230	n.s.
**LCA**	-0,1801	1,2230	n.s.	-0,1801	0,5337	n.s.	1,2230	0,5337	n.s.
**DCA**	0,0340	2,5920	***	0,0340	1,7480	*	2,5920	1,7480	n.s.
**G-CA**	-0,0016	0,2709	n.s.	-0,0016	0,0892	n.s.	0,2709	0,0892	n.s.
**G-DCA**	0,0326	2,0660	**	0,0326	1,7110	n.s.	2,0660	1,7110	n.s.
**T-CA**	0,0415	-0,3467	n.s.	0,0415	-0,5246	n.s.	-0,3467	-0,5246	n.s.
**T-DCA**	0,0710	1,1230	n.s.	0,0710	-0,1293	n.s.	1,1230	-0,1293	n.s.

**Abbreviations**: CA, cholic acid; DCA, deoxycholic acid; G, glycine; LCA, lithocholic acid; PD, Parkinson’s disease; T, taurine. Differences were considered statistically significant when p-value: *(p<0.05), **(p<0.01), ***(p<0.001), and n.s. = not significant (p>0.05).

### Mouse strains and housing

All animal experiments were approved by the “Ethics Committee for Animal Experimentation” of the Biomedical Research Institute “Alberto Sols” (CSIC-UAM) in Madrid (Spain) and carried out in accordance with the European Communities Council Directive (2010/63/EEC) and National regulations (normative RD1386/2018). Adult, male wild type C57/BL6 mice were obtained from Jackson Laboratories. The animals were housed in a cage (2–3 animals per cage) with free access to food and water under a 12 h light/dark cycle. Special care was taken to minimize pain or discomfort of animals.

### Animal model of PD

Parkinsonism was induced as previously described [61]. Briefly, anaesthetized mice were placed in a stereotaxic apparatus (Kopf Instruments, CA) and 6-OHDA (5μg in 2μL saline with 0.02% ascorbic acid) was unilaterally injected into the *substantia nigra* *pars compacta* at the following coordinates from bregma: posterior, -3.2 mm; lateral, +2.0 mm; and ventral, +4.7 mm, with the skull flat between lambda and bregma, according to the atlas of Paxinos and Franklin [62]. Mice were then housed to recover.

### Plasma sample preparation

A volume of 25 μL of plasma were mixed with 250 μL a cold solvent mixture with ISTD (MeOH/Water/Chloroform, 9/1/1, -20°C), into 1.5 mL microtube, vortexed and centrifuged (10 min at 15000 g, 4°C) to obtain protein precipitation. Then upper phase of supernatant was split in parts: 50 μL were used for GC-MS experiment in injection vial, and 50 μL were used for other UHPLC-MS experimentations. GC-MS aliquot was evaporated and 50 μL of methoxyamine (20 mg/mL in pyridine) were added on dried extracts, then stored at room temperature in dark, during 16 hours. After that, 80 μL of N-Methyl-N-(trimethylsilyl) trifluoroacetamide (MSTFA) was added and final derivatization occurred at 40°C during 30 minutes. Samples were directly injected into GC-MS. The whole was heated at 40°C during 1h. 60 μL of H_2_0 was added and the whole was injected into UHPLC-MS. Concerning the LC-MS aliquots, the 50 μL collected supernatant was evaporated at 40°C in a pneumatically-assisted concentrator (Techne DB3, Staffordshire, UK). The LC-MS dried extracts were solubilized with 150 μL of MilliQ water. Samples were aliquoted for LC methods and backup. Biological samples and QC aliquots are kept at -80°C until injection or transferred in vials for direct analysis by UHPLC/MS. All the reagents used in this study are, if not specified, from Sigma-Aldrich.

### Widely-targeted analysis of metabolites gas chromatography (GC) coupled to a triple quadrupole (QQQ) mass spectrometer

GC-MS/MS method was performed on a 7890B gas chromatography coupled to a triple quadrupole 7000C (Agilent Technologies, Waldbronn, Germany) equipped with a High sensitivity electronic impact source (EI) operating in positive mode. Front inlet temperature was 250°C, injection was performed in splitless mode. Transfer line and ion-source temperature were 250°C and 230°C, respectively. Septum purge flow was fixed at 3 mL/min, purge flow to split vent operated at 80 mL/min during 1 min and gas saver mode was set to 15 mL/min after 5 min. Helium gas flowed through column (J&WScientificHP-5MS, 30m x 0.25 mm, i.d. 0.25 mm, d.f., Agilent Technologies Inc.) at 1 mL/min. Column temperature was held at 60°C for 1 min, then raised to 210°C (10°C/min), followed by a step to 230°C (5°C/min) and reached 325°C (15°C/min), and be hold at this temperature for 5 min.

Collision gas was nitrogen. Scan mode used was MRM for biological samples. Peak detection and integration of analytes were performed using Agilent Mass Hunter quantitative software (B.07.01).

### Untargeted analysis of metabolites by ultra-high performance liquid chromatography (UHPLC) coupled to a Q-Exactive mass spectrometer. Reversed phase acetonitrile method

The profiling experiment was performed with a Dionex Ultimate 3000 UHPLC system (Thermo Scientific) coupled to a Q-Exactive (Thermo Scientific) equipped with an electrospray source operating in both positive and negative mode and full scan mode from 100 to 1200 m/z. The Q-Exactive parameters were: sheath gas flow rate 55 au, auxiliary gas flow rate 15 au, spray voltage 3.3 kV, capillary temperature 300°C, S-Lens RF level 55 V. The mass spectrometer was calibrated with sodium acetate solution dedicated to low mass calibration.

10 μL of sample were injected on a SB-Aq column (100 mm × 2.1 mm particle size 1.8 μm) from Agilent Technologies, protected by a guard column XDB-C18 (5 mm × 2.1 mm particle size 1.8 μm) and heated at 40°C by a Pelletier oven. The gradient mobile phase consists of water with 0.2% of acetic acid (A) and acetonitrile (B). The flow rate was set to 0.3 mL/min. Initial condition is 98% phase A and 2% phase B. Molecules were then eluted using a gradient from 2% to 95% phase B in 22 min. The column was washed using 95% mobile phase B for 2 minutes and equilibrated using 2% mobile phase B for 4 min.

The autosampler was kept at 4°C. Peak detection and integration were performed using the Thermo Xcalibur quantitative software (3.1.).

### Quantification and statistical analysis

Data are reported as Box and whisker plots (mean, first and third quartiles, and maximum and minimum values) or mean ± standard error of the mean (SEM). The number of independent data points (*n*) is indicated in the corresponding figure or in the Supplementary Tables 1 and 2. It’s important to note that not all metabolites are detected properly in all plasma samples. For this reason, the number of patients shown for each metabolite may suffer slight and punctual variations. For statistical analyses, *p* values were calculated by one-way ANOVA test (analyzing the metabolites individually) (Figures 2–5), multiple tests with false discovery rate (FDR) (analyzing the metabolites as a whole, being part of a specific metabolic pathway) (Supplementary Figures 2 and 3) and Pearson’s correlation coefficients with their 95% confidence interval was also used (Pearson’s correlation coefficient ® (Figure 1 and Supplementary Figure 1) and their p-value are shown). Clusterings and heatmaps have been performed using ‘‘dist’’ and ‘‘hclust’’ functions, using Euclidean distance method. (Prism version 7, GraphPad Software). Differences were considered statistically significant when *p*-values: * (p<0.05), ** (p<0.01), *** (p<0.001), and n.s. = not significant (p>0.05).

### Ethics approval

Approval from the “Ethical Board” of Hospital Donostia (ALM-LRRK2-2016-01) and “Ethics Committee for Animal Experimentation” of the Biomedical Research Institute “Alberto Sols” (CSIC-UAM) in Madrid (Spain), in accordance with the European Communities Council Directive (2010/63/EEC) and National regulations (normative RD1386/2018) was obtained prior to the experiments.

## Supplementary Material

Supplementary Figures

Supplementary Table 1

Supplementary Table 2

## References

[1] Farrer MJ. Genetics of Parkinson disease: paradigm shifts and future prospects. Nat Rev Genet. 2006; 7:306–18. 10.1038/nrg1831 16543934

[2] Paisán-Ruíz C, Jain S, Evans EW, Gilks WP, Simón J, van der Brug M, López de Munain A, Aparicio S, Gil AM, Khan N, Johnson J, Martinez JR, Nicholl D, et al. Cloning of the gene containing mutations that cause PARK8-linked Parkinson’s disease. Neuron. 2004; 44:595–600. 10.1016/j.neuron.2004.10.023 15541308

[3] Healy DG, Falchi M, O’Sullivan SS, Bonifati V, Durr A, Bressman S, Brice A, Aasly J, Zabetian CP, Goldwurm S, Ferreira JJ, Tolosa E, Kay DM, et al, and International LRRK2 Consortium. Phenotype, genotype, and worldwide genetic penetrance of LRRK2-associated Parkinson’s disease: a case-control study. Lancet Neurol. 2008; 7:583–90. 10.1016/S1474-4422(08)70117-0 18539534 PMC2832754

[4] Mata IF, Kachergus JM, Taylor JP, Lincoln S, Aasly J, Lynch T, Hulihan MM, Cobb SA, Wu RM, Lu CS, Lahoz C, Wszolek ZK, Farrer MJ. Lrrk2 pathogenic substitutions in Parkinson’s disease. Neurogenetics. 2005; 6:171–77. 10.1007/s10048-005-0005-1 16172858

[5] Obeso JA, Stamelou M, Goetz CG, Poewe W, Lang AE, Weintraub D, Burn D, Halliday GM, Bezard E, Przedborski S, Lehericy S, Brooks DJ, Rothwell JC, et al. Past, present, and future of Parkinson’s disease: a special essay on the 200^th^ anniversary of the shaking palsy. Mov Disord. 2017; 32:1264–310. 10.1002/mds.27115 28887905 PMC5685546

[6] Connolly BS, Lang AE. Pharmacological treatment of Parkinson disease: a review. JAMA. 2014; 311:1670–83. 10.1001/jama.2014.3654 24756517

[7] Shao Y, Le W. Recent advances and perspectives of metabolomics-based investigations in Parkinson’s disease. Mol Neurodegener. 2019; 14:3. 10.1186/s13024-018-0304-2 30634989 PMC6330496

[8] Maetzler W, Schmid SP, Wurster I, Liepelt I, Gaenslen A, Gasser T, Berg D. Reduced but not oxidized cerebrospinal fluid glutathione levels are lowered in lewy body diseases. Mov Disord. 2011; 26:176–81. 10.1002/mds.23358 20842692

[9] Guo X, Song W, Chen K, Chen X, Zheng Z, Cao B, Huang R, Zhao B, Wu Y, Shang HF. The serum lipid profile of Parkinson’s disease patients: a study from China. Int J Neurosci. 2015; 125:838–44. 10.3109/00207454.2014.979288 25340257

[10] Luan H, Liu LF, Meng N, Tang Z, Chua KK, Chen LL, Song JX, Mok VC, Xie LX, Li M, Cai Z. LC-MS-based urinary metabolite signatures in idiopathic Parkinson’s disease. J Proteome Res. 2015; 14:467–78. 10.1021/pr500807t 25271123

[11] Luan H, Liu LF, Tang Z, Mok VC, Li M, Cai Z. Elevated excretion of biopyrrin as a new marker for idiopathic Parkinson’s disease. Parkinsonism Relat Disord. 2015; 21:1371–72. 10.1016/j.parkreldis.2015.09.009 26372622

[12] Lin A, Zheng W, He Y, Tang W, Wei X, He R, Huang W, Su Y, Huang Y, Zhou H, Xie H. Gut microbiota in patients with Parkinson’s disease in southern China. Parkinsonism Relat Disord. 2018; 53:82–88. 10.1016/j.parkreldis.2018.05.007 29776865

[13] Saiki S, Sasazawa Y, Fujimaki M, Kamagata K, Kaga N, Taka H, Li Y, Souma S, Hatano T, Imamichi Y, Furuya N, Mori A, Oji Y, et al. A metabolic profile of polyamines in Parkinson disease: a promising biomarker. Ann Neurol. 2019; 86:251–63. 10.1002/ana.25516 31155745 PMC6772170

[14] Bogdanov M, Matson WR, Wang L, Matson T, Saunders-Pullman R, Bressman SS, Flint Beal M. Metabolomic profiling to develop blood biomarkers for Parkinson’s disease. Brain. 2008; 131:389–96. 10.1093/brain/awm304 18222993

[15] Chang KH, Cheng ML, Tang HY, Huang CY, Wu YR, Chen CM. Alternations of metabolic profile and kynurenine metabolism in the plasma of Parkinson’s disease. Mol Neurobiol. 2018; 55:6319–28. 10.1007/s12035-017-0845-3 29294246

[16] Ahmed SS, Santosh W, Kumar S, Christlet HT. Metabolic profiling of Parkinson’s disease: evidence of biomarker from gene expression analysis and rapid neural network detection. J Biomed Sci. 2009; 16:63. 10.1186/1423-0127-16-63 19594911 PMC2720938

[17] Ikeda K, Nakamura Y, Kiyozuka T, Aoyagi J, Hirayama T, Nagata R, Ito H, Iwamoto K, Murata K, Yoshii Y, Kawabe K, Iwasaki Y. Serological profiles of urate, paraoxonase-1, ferritin and lipid in Parkinson’s disease: changes linked to disease progression. Neurodegener Dis. 2011; 8:252–58. 10.1159/000323265 21282940

[18] Huang X, Chen H, Miller WC, Mailman RB, Woodard JL, Chen PC, Xiang D, Murrow RW, Wang YZ, Poole C. Lower low-density lipoprotein cholesterol levels are associated with Parkinson’s disease. Mov Disord. 2007; 22:377–81. 10.1002/mds.21290 17177184 PMC1906875

[19] Schroeder F, Jefferson JR, Kier AB, Knittel J, Scallen TJ, Wood WG, Hapala I. Membrane cholesterol dynamics: cholesterol domains and kinetic pools. Proc Soc Exp Biol Med. 1991; 196:235–52. 10.3181/00379727-196-43185 1998001

[20] Huang X, Sterling NW, Du G, Sun D, Stetter C, Kong L, Zhu Y, Neighbors J, Lewis MM, Chen H, Hohl RJ, Mailman RB. Brain cholesterol metabolism and Parkinson’s disease. Mov Disord. 2019; 34:386–95. 10.1002/mds.27609 30681742 PMC6420391

[21] Jin U, Park SJ, Park SM. Cholesterol metabolism in the brain and its association with Parkinson’s disease. Exp Neurobiol. 2019; 28:554–67. 10.5607/en.2019.28.5.554 31698548 PMC6844833

[22] García-Sanz P, Orgaz L, Bueno-Gil G, Espadas I, Rodríguez-Traver E, Kulisevsky J, Gutierrez A, Dávila JC, González-Polo RA, Fuentes JM, Mir P, Vicario C, Moratalla R. N370S-GBA1 mutation causes lysosomal cholesterol accumulation in Parkinson’s disease. Mov Disord. 2017; 32:1409–22. 10.1002/mds.27119 28779532

[23] Hasuike Y, Endo T, Koroyasu M, Matsui M, Mori C, Yamadera M, Fujimura H, Sakoda S. Bile acid abnormality induced by intestinal dysbiosis might explain lipid metabolism in Parkinson’s disease. Med Hypotheses. 2020; 134:109436. 10.1016/j.mehy.2019.109436 31678900

[24] Graham SF, Rey NL, Ugur Z, Yilmaz A, Sherman E, Maddens M, Bahado-Singh RO, Becker K, Schulz E, Meyerdirk LK, Steiner JA, Ma J, Brundin P. Metabolomic profiling of bile acids in an experimental model of prodromal Parkinson’s disease. Metabolites. 2018; 8:71. 10.3390/metabo8040071 30384419 PMC6316593

[25] Greenamyre JT, Sanders LH, Gasser T. Fruit flies, bile acids, and Parkinson disease: a mitochondrial connection? Neurology. 2015; 85:838–39. 10.1212/WNL.0000000000001912 26253445

[26] Klag MJ, Ford DE, Mead LA, He J, Whelton PK, Liang KY, Levine DM. Serum cholesterol in young men and subsequent cardiovascular disease. N Engl J Med. 1993; 328:313–18. 10.1056/NEJM199302043280504 8419817

[27] Lewington S, Whitlock G, Clarke R, Sherliker P, Emberson J, Halsey J, Qizilbash N, Peto R, Collins R, and Prospective Studies Collaboration. Blood cholesterol and vascular mortality by age, sex, and blood pressure: a meta-analysis of individual data from 61 prospective studies with 55,000 vascular deaths. Lancet. 2007; 370:1829–39. 10.1016/S0140-6736(07)61778-4 18061058

[28] de Lau LM, Koudstaal PJ, Hofman A, Breteler MM. Serum cholesterol levels and the risk of Parkinson’s disease. Am J Epidemiol. 2006; 164:998–1002. 10.1093/aje/kwj283 16905642

[29] Hu G, Antikainen R, Jousilahti P, Kivipelto M, Tuomilehto J. Total cholesterol and the risk of Parkinson disease. Neurology. 2008; 70:1972–79. 10.1212/01.wnl.0000312511.62699.a8 18401018

[30] Gudala K, Bansal D, Muthyala H. Role of serum cholesterol in Parkinson’s disease: a meta-analysis of evidence. J Parkinsons Dis. 2013; 3:363–70. 10.3233/JPD-130196 23948990

[31] Maiuolo J, Oppedisano F, Gratteri S, Muscoli C, Mollace V. Regulation of uric acid metabolism and excretion. Int J Cardiol. 2016; 213:8–14. 10.1016/j.ijcard.2015.08.109 26316329

[32] Andreadou E, Nikolaou C, Gournaras F, Rentzos M, Boufidou F, Tsoutsou A, Zournas C, Zissimopoulos V, Vassilopoulos D. Serum uric acid levels in patients with Parkinson’s disease: their relationship to treatment and disease duration. Clin Neurol Neurosurg. 2009; 111:724–28. 10.1016/j.clineuro.2009.06.012 19632030

[33] Weisskopf MG, O’Reilly E, Chen H, Schwarzschild MA, Ascherio A. Plasma urate and risk of Parkinson’s disease. Am J Epidemiol. 2007; 166:561–67. 10.1093/aje/kwm127 17584757 PMC2391073

[34] Cipriani S, Chen X, Schwarzschild MA. Urate: a novel biomarker of Parkinson’s disease risk, diagnosis and prognosis. Biomark Med. 2010; 4:701–12. 10.2217/bmm.10.94 20945982 PMC3049925

[35] Davis JW, Grandinetti A, Waslien CI, Ross GW, White LR, Morens DM. Observations on serum uric acid levels and the risk of idiopathic Parkinson’s disease. Am J Epidemiol. 1996; 144:480–84. 10.1093/oxfordjournals.aje.a008954 8781463

[36] Ascherio A, LeWitt PA, Xu K, Eberly S, Watts A, Matson WR, Marras C, Kieburtz K, Rudolph A, Bogdanov MB, Schwid SR, Tennis M, Tanner CM, et al, and Parkinson Study Group DATATOP Investigators. Urate as a predictor of the rate of clinical decline in Parkinson disease. Arch Neurol. 2009; 66:1460–68. 10.1001/archneurol.2009.247 19822770 PMC2795011

[37] Singh JA, Cleveland JD. Gout and the risk of Parkinson’s disease in older adults: a study of U.S. Medicare data. BMC Neurol. 2019; 19:4. 10.1186/s12883-018-1234-x 30611222 PMC6321725

[38] De Vera M, Rahman MM, Rankin J, Kopec J, Gao X, Choi H. Gout and the risk of Parkinson’s disease: a cohort study. Arthritis Rheum. 2008; 59:1549–54. 10.1002/art.24193 18975349

[39] Zhang Y, Lee FY, Barrera G, Lee H, Vales C, Gonzalez FJ, Willson TM, Edwards PA. Activation of the nuclear receptor FXR improves hyperglycemia and hyperlipidemia in diabetic mice. Proc Natl Acad Sci USA. 2006; 103:1006–11. 10.1073/pnas.0506982103 16410358 PMC1347977

[40] Lang AE, Lozano AM. Parkinson’s disease. First of two parts. N Engl J Med. 1998; 339:1044–53. 10.1056/NEJM199810083391506 9761807

[41] Yakhine-Diop SM, Bravo-San Pedro JM, Gómez-Sánchez R, Pizarro-Estrella E, Rodríguez-Arribas M, Climent V, Aiastui A, López de Munain A, Fuentes JM, González-Polo RA. G2019S LRRK2 mutant fibroblasts from Parkinson’s disease patients show increased sensitivity to neurotoxin 1-methyl-4-phenylpyridinium dependent of autophagy. Toxicology. 2014; 324:1–9. 10.1016/j.tox.2014.07.001 25017139

[42] Bravo-San Pedro JM, Niso-Santano M, Gómez-Sánchez R, Pizarro-Estrella E, Aiastui-Pujana A, Gorostidi A, Climent V, López de Maturana R, Sanchez-Pernaute R, López de Munain A, Fuentes JM, González-Polo RA. The LRRK2 G2019S mutant exacerbates basal autophagy through activation of the MEK/ERK pathway. Cell Mol Life Sci. 2013; 70:121–36. 10.1007/s00018-012-1061-y 22773119 PMC11113213

[43] Yakhine-Diop SM, Niso-Santano M, Rodríguez-Arribas M, Gómez-Sánchez R, Martínez-Chacón G, Uribe-Carretero E, Navarro-García JA, Ruiz-Hurtado G, Aiastui A, Cooper JM, López de Munaín A, Bravo-San Pedro JM, González-Polo RA, Fuentes JM. Impaired mitophagy and protein acetylation levels in fibroblasts from Parkinson’s disease patients. Mol Neurobiol. 2019; 56:2466–81. 10.1007/s12035-018-1206-6 30032424

[44] Johnson CH, Ivanisevic J, Siuzdak G. Metabolomics: beyond biomarkers and towards mechanisms. Nat Rev Mol Cell Biol. 2016; 17:451–59. 10.1038/nrm.2016.25 26979502 PMC5729912

[45] Lewitt PA, Li J, Lu M, Beach TG, Adler CH, Guo L, and Arizona Parkinson’s Disease Consortium. 3-hydroxykynurenine and other Parkinson’s disease biomarkers discovered by metabolomic analysis. Mov Disord. 2013; 28:1653–60. 10.1002/mds.25555 23873789

[46] Amara AW, Standaert DG. Metabolomics and the search for biomarkers in Parkinson’s disease. Mov Disord. 2013; 28:1620–21. 10.1002/mds.25644 24105981 PMC4050667

[47] LeWitt PA, Li J, Lu M, Guo L, Auinger P, and Parkinson Study Group–DATATOP Investigators. Metabolomic biomarkers as strong correlates of Parkinson disease progression. Neurology. 2017; 88:862–69. 10.1212/WNL.0000000000003663 28179471 PMC5331866

[48] Peskind ER, Riekse R, Quinn JF, Kaye J, Clark CM, Farlow MR, Decarli C, Chabal C, Vavrek D, Raskind MA, Galasko D. Safety and acceptability of the research lumbar puncture. Alzheimer Dis Assoc Disord. 2005; 19:220–25. 10.1097/01.wad.0000194014.43575.fd 16327349

[49] Fujimaki M, Saiki S, Li Y, Kaga N, Taka H, Hatano T, Ishikawa KI, Oji Y, Mori A, Okuzumi A, Koinuma T, Ueno SI, Imamichi Y, et al. Serum caffeine and metabolites are reliable biomarkers of early Parkinson disease. Neurology. 2018; 90:e404–11. 10.1212/WNL.0000000000004888 29298852 PMC5791797

[50] Hatano T, Saiki S, Okuzumi A, Mohney RP, Hattori N. Identification of novel biomarkers for Parkinson’s disease by metabolomic technologies. J Neurol Neurosurg Psychiatry. 2016; 87:295–301. 10.1136/jnnp-2014-309676 25795009

[51] Johansen KK, Wang L, Aasly JO, White LR, Matson WR, Henchcliffe C, Beal MF, Bogdanov M. Metabolomic profiling in LRRK2-related Parkinson’s disease. PLoS One. 2009; 4:e7551. 10.1371/journal.pone.0007551 19847307 PMC2761616

[52] Kim A, Nigmatullina R, Zalyalova Z, Soshnikova N, Krasnov A, Vorobyeva N, Georgieva S, Kudrin V, Narkevich V, Ugrumov M. Upgraded methodology for the development of early diagnosis of Parkinson’s disease based on searching blood markers in patients and experimental models. Mol Neurobiol. 2019; 56:3437–50. 10.1007/s12035-018-1315-2 30128652

[53] Huang X, Abbott RD, Petrovitch H, Mailman RB, Ross GW. Low LDL cholesterol and increased risk of Parkinson’s disease: prospective results from honolulu-asia aging study. Mov Disord. 2008; 23:1013–18. 10.1002/mds.22013 18381649

[54] Zhao H, Wang C, Zhao N, Li W, Yang Z, Liu X, Le W, Zhang X. Potential biomarkers of Parkinson’s disease revealed by plasma metabolic profiling. J Chromatogr B Analyt Technol Biomed Life Sci. 2018; 1081:101–08. 10.1016/j.jchromb.2018.01.025 29518718

[55] Marksteiner J, Blasko I, Kemmler G, Koal T, Humpel C. Bile acid quantification of 20 plasma metabolites identifies lithocholic acid as a putative biomarker in Alzheimer’s disease. Metabolomics. 2018; 14:1. 10.1007/s11306-017-1297-5 29249916 PMC5725507

[56] Chiang JY. Regulation of bile acid synthesis: pathways, nuclear receptors, and mechanisms. J Hepatol. 2004; 40:539–51. 10.1016/j.jhep.2003.11.006 15123373

[57] Einarsson K, Bergström M, Eklöf R, Nord CE, Björkhem I. Comparison of the proportion of unconjugated to total serum cholic acid and the [14C]-xylose breath test in patients with suspected small intestinal bacterial overgrowth. Scand J Clin Lab Invest. 1992; 52:425–30. 10.3109/00365519209088378 1514020

[58] Schroeder BO, Bäckhed F. Signals from the gut microbiota to distant organs in physiology and disease. Nat Med. 2016; 22:1079–89. 10.1038/nm.4185 27711063

[59] Sharon G, Sampson TR, Geschwind DH, Mazmanian SK. The central nervous system and the gut microbiome. Cell. 2016; 167:915–32. 10.1016/j.cell.2016.10.027 27814521 PMC5127403

[60] Tan AH, Mahadeva S, Thalha AM, Gibson PR, Kiew CK, Yeat CM, Ng SW, Ang SP, Chow SK, Tan CT, Yong HS, Marras C, Fox SH, Lim SY. Small intestinal bacterial overgrowth in Parkinson’s disease. Parkinsonism Relat Disord. 2014; 20:535–40. 10.1016/j.parkreldis.2014.02.019 24637123

[61] Morales-Garcia JA, Aguilar-Morante D, Hernandez-Encinas E, Alonso-Gil S, Gil C, Martinez A, Santos A, Perez-Castillo A. Silencing phosphodiesterase 7B gene by lentiviral-shRNA interference attenuates neurodegeneration and motor deficits in hemiparkinsonian mice. Neurobiol Aging. 2015; 36:1160–73. 10.1016/j.neurobiolaging.2014.10.008 25457552

[62] Paxinos G, Franklin KBJ. The Mouse Brain in Stereotaxic Coordinates. Second Edition, Academic Press, San Diego 2001.

