# Supplementary material for: Metabolic alterations in plasma from patients with familial and idiopathic Parkinson’s disease
Source: Aging (Albany NY). 2020 Sep 9;12(17):16690–708. doi: 10.18632/aging.103992 (PMC7521510; doi:10.18632/aging.103992)
Supplement: Supplementary Figures [file aging-12-103992-s003..pdf]

SUPPLEMENTARY FIGURES

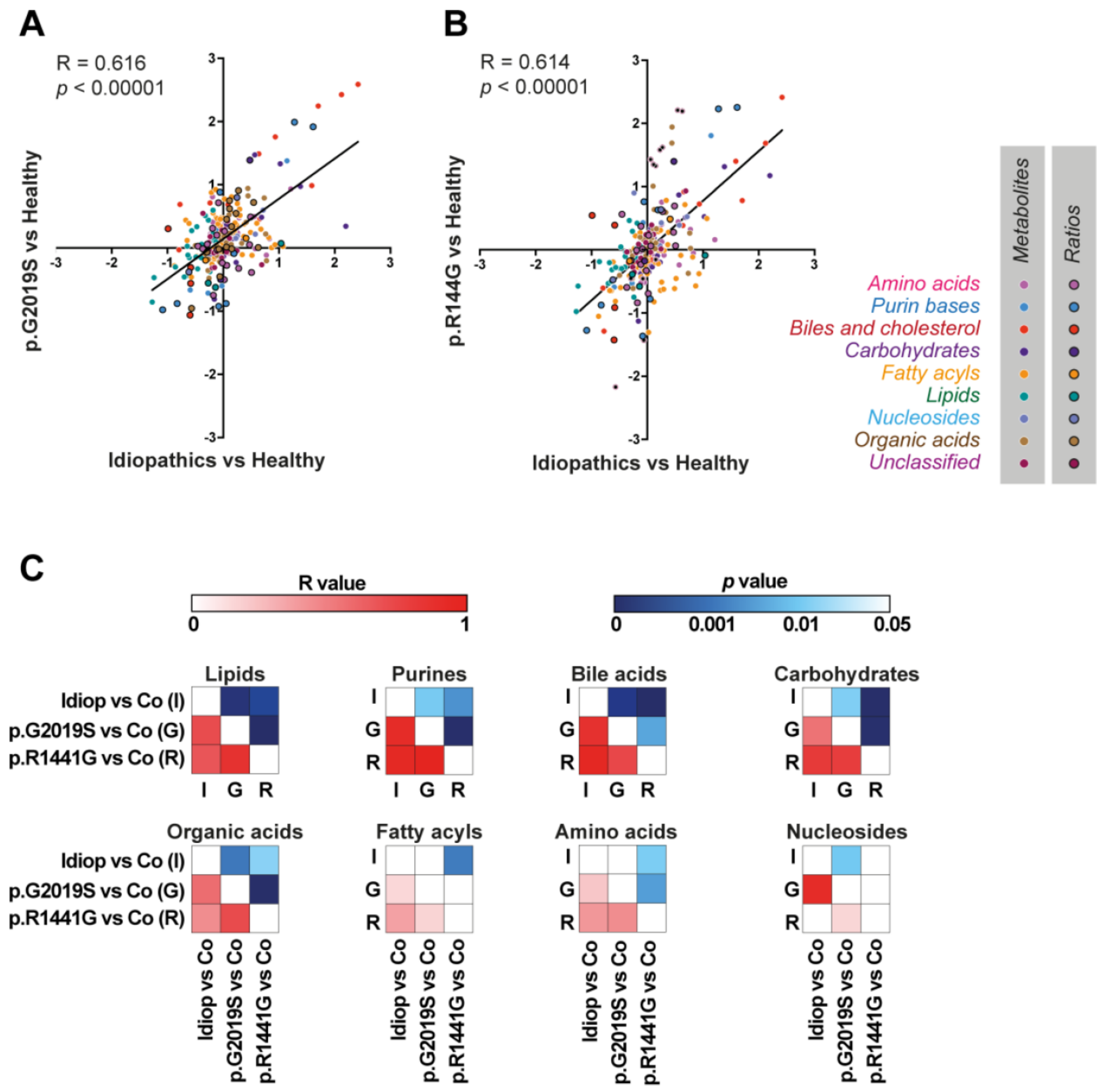

Supplementary Figure 1. Pearson's correlations in plasma samples between changes metabolite correlations (Log2FC) in idiopathic and p.G2019S PD patients (**A**) or idiopathic and p.R1441G PD patients (**B**). The correlation analysis divided by subtypes of metabolites (lipids, purines, bile acids, carbohydrates, organic acids, fatty acyls, amino acids and nucleosides) is shown by color codes (p values and Pearson's coefficients of correlation (R)) (**C**).

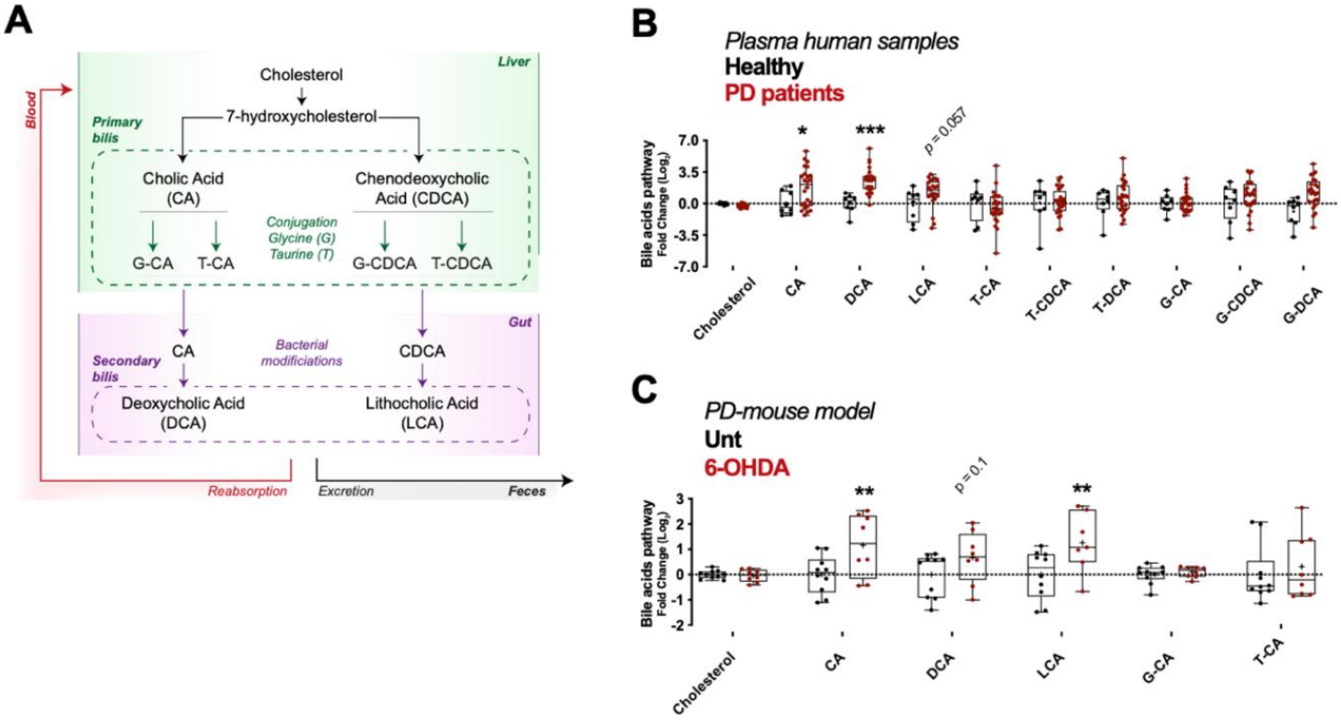

Supplementary Figure 2. Scheme of the pathways of bile synthesis from cholesterol (**A**). Box and whisker plots of fold change (Log2) concentrations of bile acid pathway metabolites (cholesterol, CA, DCA, LCA, G-conjugated bile acids and T-conjugated bile acids) in human plasma in the control group (healthy) and PD individuals (**B**) and PD-mouse model (**C**). Abbreviations: 6-OHDA, 6-hydroxydopamine; CA, cholic acid; DCA, deoxycholic acid; G, glycine; LCA, lithocholic acid; PD, Parkinson's disease; T, taurine; Unt, untreated. Differences were considered statistically significant when p-values: * (p<0.05), ** (p<0.01), *** (p<0.001).

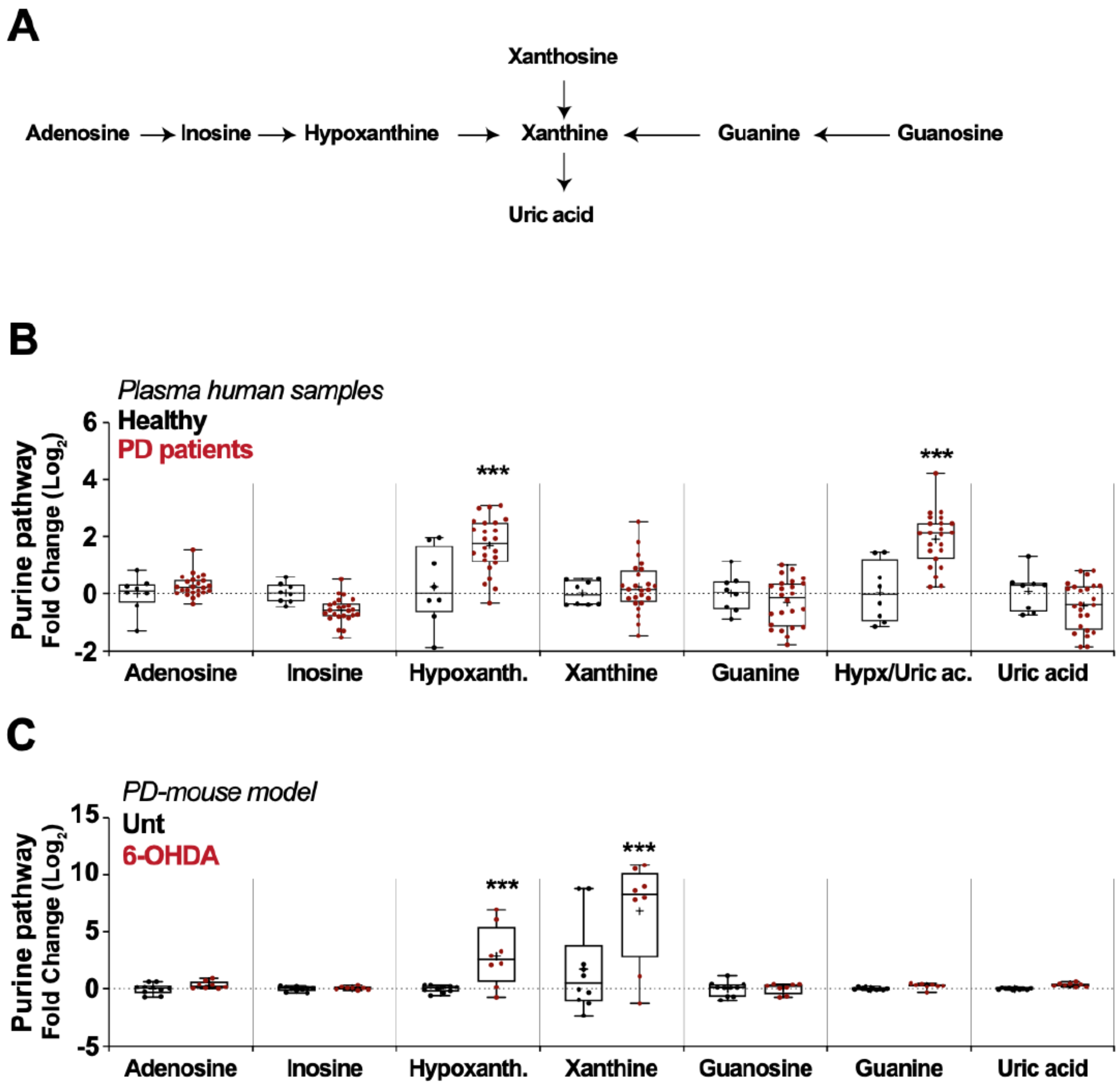

Supplementary Figure 3. Scheme of the purine metabolism pathways (**A**). Box and whisker plots of fold change (Log2) concentrations of purine acids pathway metabolites (uric acid, hypoxanthine, xanthine, inosine, adenosine, guanine, guanosine and hypoxanthine/uric acid ratio) in human plasma from the control group (healthy) and PD individuals (**B**) and our PD-mouse model (**C**). Abbreviations: 6-OHDA, 6-hydroxydopamine; PD, Parkinson's disease; Unt, untreated. Differences were considered statistically significant when p-values: *** ($p<0.001$).
