# Supplementary material for: Metabolic alterations in plasma from patients with familial and idiopathic Parkinson’s disease
Source: Aging (Albany NY). 2020 Sep 9;12(17):16690–708. doi: 10.18632/aging.103992 (PMC7521510; doi:10.18632/aging.103992)
Supplement: Supplementary Table 1 [file aging-12-103992-s002..docx]

**Supplementary Table 1. Metabolite changes in plasma from controls and PD patients.**

| **Human plasma samples** | | **Area values** | | | | | | | | | | | | | | | **Fold Change (Log2) PD patients vs controls (Co)** | | | | | | | |
| --- | --- | --- | --- | --- | --- | --- | --- | --- | --- | --- | --- | --- | --- | --- | --- | --- | --- | --- | --- | --- | --- | --- | --- | --- |
|  |  | **Healthy (Controls)** | | | **Idiopathic** | |  | **G2019S** | |  | **R1441G** | |  | **All PD patients** | |  | **Idiopathics vs Co** | | **G2019S vs Co** | | **R1441G vs Co** | | **All PD vs Co** | |
| **Group** | **Metabolites** | **N** | **Average** | **SEM** | **N** | **Average** | **SEM** | **N** | **Average** | **SEM** | **N** | **Average** | **SEM** | **N** | **Average** | **SEM** | **FC** | **TTEST (pvalue)** | **FC** | **TTEST (pvalue)** | **FC** | **TTEST (pvalue)** | **FC** | **TTEST (pvalue)** |
| **Amino acids** | **2-aminoadipic acid** | 8 | -0,0077 | 0,2654 | 8 | 0,0240 | 0,1523 | 8 | 0,3774 | 0,1710 | 8 | 0,2074 | 0,1108 | 24 | 0,2030 | 0,0864 | 0,032 | 0,919 | 0,385 | 0,243 | 0,215 | 0,467 | 0,211 | 0,328 |
|  | **3-methylhistidine** | 8 | 0,0244 | 0,2161 | 8 | 0,7992 | 0,4140 | 8 | -0,0897 | 0,2690 | 8 | 0,3475 | 0,2037 | 24 | 0,3523 | 0,1862 | 0,775 | 0,119 | -0,114 | 0,746 | 0,323 | 0,295 | 0,328 | 0,353 |
|  | **4-hydroxyproline** | 8 | 0,0325 | 0,2790 | 8 | 0,8607 | 0,5366 | 8 | 0,1765 | 0,3860 | 8 | -0,0190 | 0,1545 | 24 | 0,3394 | 0,2301 | 0,828 | 0,192 | 0,144 | 0,767 | -0,051 | 0,874 | 0,307 | 0,483 |
|  | **5-oxoproline** | 8 | -0,0052 | 0,1392 | 8 | 0,0593 | 0,0619 | 8 | -0,0075 | 0,1384 | 8 | -0,2524 | 0,1049 | 24 | -0,0669 | 0,0650 | 0,065 | 0,678 | -0,002 | 0,991 | -0,247 | 0,178 | -0,062 | 0,658 |
|  | **Alanine** | 8 | 0,0181 | 0,3288 | 8 | 0,3040 | 0,1088 | 8 | 0,4938 | 0,1956 | 8 | 0,6463 | 0,0867 | 24 | 0,4814 | 0,0818 | 0,286 | 0,423 | 0,476 | 0,234 | 0,628 | 0,086 | 0,463 | 0,056 |
|  | **Arginine** | 8 | 0,0358 | 0,1832 | 8 | -0,0165 | 0,1399 | 8 | 0,1473 | 0,1222 | 8 | -0,4785 | 0,2370 | 24 | -0,1159 | 0,1107 | -0,052 | 0,824 | 0,112 | 0,621 | -0,514 | 0,108 | -0,152 | 0,494 |
|  | **Asparagine** | 8 | 0,0430 | 0,2262 | 8 | -0,1004 | 0,2073 | 8 | 0,3144 | 0,2006 | 8 | -0,2005 | 0,1596 | 24 | 0,0045 | 0,1148 | -0,143 | 0,647 | 0,271 | 0,385 | -0,244 | 0,394 | -0,039 | 0,872 |
|  | **Aspartic acid** | 8 | 0,0515 | 0,2819 | 8 | 0,2799 | 0,1443 | 8 | 0,3450 | 0,2216 | 8 | 0,3927 | 0,1864 | 24 | 0,3392 | 0,1035 | 0,228 | 0,483 | 0,294 | 0,427 | 0,341 | 0,330 | 0,288 | 0,240 |
|  | **Betaine;Valine** | 8 | -0,0031 | 0,0980 | 8 | 0,2589 | 0,0764 | 8 | 0,1658 | 0,0667 | 8 | 0,1242 | 0,0632 | 24 | 0,1830 | 0,0398 | 0,262 | 0,053 | 0,169 | 0,176 | 0,127 | 0,294 | 0,186 | 0,044 |
|  | **Citrulline** | 8 | 0,0136 | 0,2264 | 8 | 0,0161 | 0,1121 | 8 | 0,1630 | 0,1792 | 8 | -0,3650 | 0,2343 | 24 | -0,0620 | 0,1107 | 0,002 | 0,992 | 0,149 | 0,613 | -0,379 | 0,265 | -0,076 | 0,746 |
|  | **Creatine** | 8 | 0,0862 | 0,3834 | 7 | 0,3461 | 0,2092 | 7 | 0,8026 | 0,2635 | 7 | 0,8404 | 0,2449 | 21 | 0,6630 | 0,1408 | 0,260 | 0,578 | 0,716 | 0,159 | 0,754 | 0,133 | 0,577 | 0,088 |
|  | **Creatinine** | 8 | -0,0091 | 0,0658 | 8 | 0,1452 | 0,1395 | 8 | -0,0974 | 0,1593 | 8 | -0,1519 | 0,1025 | 24 | -0,0347 | 0,0796 | 0,154 | 0,334 | -0,088 | 0,616 | -0,143 | 0,261 | -0,026 | 0,860 |
|  | **Cystine;Cysteine** | 8 | 0,1550 | 0,4957 | 8 | -0,0366 | 0,4737 | 8 | 0,9643 | 0,3904 | 8 | -0,0854 | 0,3867 | 24 | 0,2808 | 0,2521 | -0,192 | 0,784 | 0,809 | 0,220 | -0,240 | 0,708 | 0,126 | 0,811 |
|  | **Dimethylarginine** | 8 | 0,0139 | 0,0570 | 8 | -0,1173 | 0,0844 | 8 | 0,1477 | 0,1125 | 8 | -0,2768 | 0,1127 | 24 | -0,0821 | 0,0680 | -0,131 | 0,219 | 0,134 | 0,307 | -0,291 | 0,037 | -0,096 | 0,442 |
|  | **Dimethylglycine** | 8 | 0,0209 | 0,1245 | 8 | 0,0156 | 0,0943 | 8 | 0,2541 | 0,1553 | 8 | 0,0066 | 0,0719 | 24 | 0,0921 | 0,0667 | -0,005 | 0,973 | 0,233 | 0,261 | -0,014 | 0,922 | 0,071 | 0,604 |
|  | **GABA** | 8 | 0,0164 | 0,1523 | 8 | 0,5888 | 0,4681 | 8 | 0,0036 | 0,3279 | 8 | -0,2223 | 0,1483 | 24 | 0,1234 | 0,2011 | 0,572 | 0,264 | -0,013 | 0,972 | -0,239 | 0,280 | 0,107 | 0,770 |
|  | **Glutamic acid** | 8 | 0,0926 | 0,2912 | 8 | 0,6492 | 0,2314 | 8 | 0,5890 | 0,3238 | 8 | 0,5360 | 0,2886 | 24 | 0,5914 | 0,1569 | 0,557 | 0,157 | 0,496 | 0,273 | 0,443 | 0,298 | 0,499 | 0,129 |
|  | **Glutamine** | 8 | 0,0196 | 0,2420 | 8 | 0,1036 | 0,1640 | 8 | 0,4640 | 0,1758 | 8 | 0,1867 | 0,1538 | 24 | 0,2514 | 0,0964 | 0,084 | 0,778 | 0,444 | 0,160 | 0,167 | 0,569 | 0,232 | 0,292 |
|  | **Glycine** | 8 | 0,0536 | 0,1486 | 8 | -0,0582 | 0,1732 | 8 | -0,0859 | 0,2189 | 8 | -0,4678 | 0,1550 | 24 | -0,2040 | 0,1089 | -0,112 | 0,632 | -0,140 | 0,606 | -0,521 | 0,029 | -0,258 | 0,225 |
|  | **Histidine** | 8 | 0,1061 | 0,3548 | 8 | -0,1195 | 0,2852 | 8 | 0,3260 | 0,3007 | 8 | -0,1786 | 0,2791 | 24 | 0,0093 | 0,1659 | -0,226 | 0,628 | 0,220 | 0,644 | -0,285 | 0,539 | -0,097 | 0,785 |
|  | **Homoserine** | 8 | 0,0367 | 0,3070 | 8 | 0,1338 | 0,1411 | 8 | 0,2696 | 0,1329 | 8 | 0,0752 | 0,1626 | 24 | 0,1595 | 0,0824 | 0,097 | 0,778 | 0,233 | 0,498 | 0,039 | 0,913 | 0,123 | 0,587 |
|  | **Isoleucine** | 8 | -0,0136 | 0,2053 | 8 | 0,0744 | 0,1723 | 8 | 0,3549 | 0,3411 | 8 | 0,1937 | 0,2663 | 24 | 0,2077 | 0,1503 | 0,088 | 0,748 | 0,369 | 0,370 | 0,207 | 0,547 | 0,221 | 0,447 |
|  | **Leucine** | 8 | -0,0145 | 0,2275 | 8 | 0,0314 | 0,1679 | 8 | 0,4341 | 0,3026 | 8 | 0,2465 | 0,2037 | 24 | 0,2373 | 0,1324 | 0,046 | 0,873 | 0,449 | 0,256 | 0,261 | 0,407 | 0,252 | 0,348 |
|  | **Lysine** | 8 | 0,0243 | 0,2067 | 8 | -0,0931 | 0,1345 | 8 | 0,3330 | 0,1998 | 8 | -0,0022 | 0,1915 | 24 | 0,0792 | 0,1052 | -0,117 | 0,641 | 0,309 | 0,301 | -0,027 | 0,926 | 0,055 | 0,802 |
|  | **Methionine** | 8 | 0,0404 | 0,3182 | 8 | 0,1348 | 0,1532 | 8 | 0,5401 | 0,2559 | 8 | 0,3882 | 0,1817 | 24 | 0,3543 | 0,1166 | 0,094 | 0,793 | 0,500 | 0,241 | 0,348 | 0,359 | 0,314 | 0,255 |
|  | **N-acetylglutamic acid** | 8 | 0,0094 | 0,2777 | 8 | 0,1161 | 0,2487 | 8 | -0,0736 | 0,3334 | 8 | 0,2336 | 0,2120 | 24 | 0,0920 | 0,1510 | 0,107 | 0,779 | -0,083 | 0,851 | 0,224 | 0,532 | 0,083 | 0,789 |
|  | **N-acetylglutamine** | 8 | -0,0074 | 0,0615 | 8 | -0,1195 | 0,0601 | 8 | -0,1679 | 0,0624 | 8 | 0,0011 | 0,0364 | 24 | -0,0954 | 0,0334 | -0,112 | 0,213 | -0,160 | 0,088 | 0,009 | 0,906 | -0,088 | 0,204 |
|  | **N6-acetyllysine;N2-acetyllysine** | 6 | -0,1516 | 0,3750 | 7 | 0,1074 | 0,2689 | 8 | 0,0854 | 0,1805 | 7 | 0,2517 | 0,1425 | 22 | 0,1454 | 0,1124 | 0,259 | 0,578 | 0,237 | 0,548 | 0,403 | 0,309 | 0,297 | 0,309 |
|  | **Ornithine** | 8 | 0,0176 | 0,1834 | 8 | -0,1508 | 0,1646 | 8 | 0,1206 | 0,1222 | 8 | -0,2108 | 0,1897 | 24 | -0,0803 | 0,0939 | -0,168 | 0,505 | 0,103 | 0,647 | -0,228 | 0,401 | -0,098 | 0,617 |
|  | **Pantothenic acid** | 8 | -0,0120 | 0,2090 | 8 | -0,2253 | 0,1828 | 8 | 0,1890 | 0,1918 | 8 | -0,0095 | 0,1089 | 24 | -0,0153 | 0,0978 | -0,213 | 0,455 | 0,201 | 0,490 | 0,002 | 0,992 | -0,003 | 0,987 |
|  | **Phenylalanine** | 8 | -0,0128 | 0,1831 | 8 | -0,0001 | 0,1060 | 8 | 0,0926 | 0,1812 | 8 | -0,0393 | 0,1714 | 24 | 0,0177 | 0,0871 | 0,013 | 0,953 | 0,105 | 0,689 | -0,027 | 0,917 | 0,031 | 0,869 |
|  | **Proline betaine** | 8 | -0,1403 | 0,8130 | 8 | 1,0734 | 0,7077 | 8 | 0,7951 | 0,6806 | 8 | 0,0204 | 0,7986 | 24 | 0,6296 | 0,4137 | 1,214 | 0,279 | 0,935 | 0,393 | 0,161 | 0,890 | 0,770 | 0,375 |
|  | **Proline** | 8 | 0,0249 | 0,3604 | 8 | 0,3265 | 0,3427 | 8 | 0,5320 | 0,3913 | 8 | -0,0608 | 0,3247 | 24 | 0,2659 | 0,2019 | 0,302 | 0,554 | 0,507 | 0,357 | -0,086 | 0,862 | 0,241 | 0,558 |
|  | **Serine** | 8 | 0,0195 | 0,2486 | 8 | -0,0733 | 0,1975 | 8 | 0,1332 | 0,1495 | 8 | -0,2620 | 0,2683 | 24 | -0,0674 | 0,1211 | -0,093 | 0,774 | 0,114 | 0,701 | -0,282 | 0,454 | -0,087 | 0,734 |
|  | **Threonine** | 8 | -0,0012 | 0,2393 | 8 | 0,0801 | 0,1379 | 8 | 0,3411 | 0,1494 | 8 | -0,0732 | 0,1596 | 24 | 0,1160 | 0,0897 | 0,081 | 0,773 | 0,342 | 0,245 | -0,072 | 0,806 | 0,117 | 0,574 |
|  | **Trimethyl-lysine** | 8 | 0,0459 | 0,2363 | 8 | 0,2350 | 0,1049 | 8 | -0,0088 | 0,1496 | 8 | -0,1503 | 0,1362 | 24 | 0,0253 | 0,0798 | 0,189 | 0,476 | -0,055 | 0,848 | -0,196 | 0,484 | -0,021 | 0,916 |
|  | **Tyrosine** | 8 | -0,0043 | 0,1649 | 8 | -0,1889 | 0,2139 | 8 | 0,4040 | 0,1492 | 8 | -0,0272 | 0,1763 | 24 | 0,0626 | 0,1130 | -0,185 | 0,505 | 0,408 | 0,088 | -0,023 | 0,926 | 0,067 | 0,761 |
|  | **Valine** | 8 | -0,0137 | 0,1984 | 8 | 0,0382 | 0,1344 | 8 | 0,2055 | 0,2048 | 8 | 0,1616 | 0,1510 | 24 | 0,1351 | 0,0928 | 0,052 | 0,832 | 0,219 | 0,455 | 0,175 | 0,494 | 0,149 | 0,455 |
| **Bases** | **Guanine** | 8 | 0,0241 | 0,2292 | 8 | 0,0006 | 0,3106 | 8 | -0,5769 | 0,3164 | 8 | -0,2987 | 0,2388 | 24 | -0,2917 | 0,1678 | -0,024 | 0,952 | -0,601 | 0,146 | -0,323 | 0,346 | -0,316 | 0,332 |
|  | **Hypoxanthine** | 8 | 0,2592 | 0,4698 | 8 | 1,4049 | 0,4229 | 8 | 1,6367 | 0,2501 | 8 | 2,0674 | 0,2975 | 24 | 1,7030 | 0,1917 | 1,146 | 0,091 | 1,378 | 0,021 | 1,808 | 0,006 | 1,444 | 0,002 |
|  | **Inosine** | 8 | 0,0368 | 0,1226 | 8 | -0,5542 | 0,0921 | 8 | -0,6292 | 0,1599 | 8 | -0,5513 | 0,2126 | 24 | -0,5782 | 0,0900 | -0,591 | 0,002 | -0,666 | 0,005 | -0,588 | 0,031 | -0,615 | 0,001 |
|  | **Thymine** | 8 | -0,0433 | 0,1411 | 8 | 0,0133 | 0,1451 | 8 | -0,0996 | 0,2013 | 8 | 0,1975 | 0,1757 | 24 | 0,0371 | 0,1002 | 0,057 | 0,784 | -0,056 | 0,822 | 0,241 | 0,303 | 0,080 | 0,679 |
|  | **Uric acid** | 8 | 0,0962 | 0,2454 | 8 | -0,1758 | 0,3450 | 8 | -0,6180 | 0,3123 | 8 | -0,4323 | 0,2329 | 24 | -0,4087 | 0,1700 | -0,272 | 0,531 | -0,714 | 0,094 | -0,528 | 0,141 | -0,505 | 0,134 |
|  | **Xanthine** | 8 | 0,0317 | 0,1500 | 8 | -0,0441 | 0,3079 | 8 | 0,3215 | 0,3228 | 8 | 0,4606 | 0,3241 | 24 | 0,2460 | 0,1811 | -0,076 | 0,828 | 0,290 | 0,429 | 0,429 | 0,250 | 0,214 | 0,519 |
| **Bilic acid** | **CA** | 7 | 0,0898 | 0,5309 | 8 | 1,7935 | 0,7205 | 8 | 2,8477 | 0,6551 | 7 | 0,8704 | 0,7894 | 23 | 1,8793 | 0,4309 | 1,704 | 0,086 | 2,758 | 0,007 | 0,781 | 0,428 | 1,789 | 0,042 |
|  | **Cholesterol** | 7 | -0,0165 | 0,0502 | 8 | -0,3496 | 0,0560 | 8 | -0,1768 | 0,0755 | 8 | -0,2157 | 0,0609 | 24 | -0,2474 | 0,0389 | -0,333 | 0,001 | -0,160 | 0,111 | -0,199 | 0,028 | -0,231 | 0,006 |
|  | **DCA** | 8 | 0,0339 | 0,3650 | 8 | 2,4529 | 0,6045 | 8 | 2,6220 | 0,6312 | 8 | 2,4479 | 0,2370 | 24 | 2,5076 | 0,2889 | 2,419 | 0,004 | 2,588 | 0,003 | 2,414 | 0,000 | 2,474 | 0,000 |
|  | **G-CA** | 8 | -0,0016 | 0,3558 | 8 | 0,0870 | 0,2112 | 8 | 0,6843 | 0,5069 | 8 | -0,1628 | 0,2594 | 24 | 0,2029 | 0,2072 | 0,089 | 0,833 | 0,686 | 0,287 | -0,161 | 0,720 | 0,204 | 0,625 |
|  | **G-CDCA** | 8 | 0,0233 | 0,7410 | 8 | 0,6608 | 0,4014 | 8 | 1,5106 | 0,7980 | 8 | 0,4089 | 0,5201 | 24 | 0,8601 | 0,3436 | 0,638 | 0,462 | 1,487 | 0,194 | 0,386 | 0,677 | 0,837 | 0,261 |
|  | **G-DCA** | 8 | 0,0326 | 0,5367 | 8 | 2,1548 | 0,4537 | 8 | 2,4600 | 0,7998 | 8 | 1,7198 | 0,5287 | 24 | 2,1116 | 0,3437 | 2,122 | 0,009 | 2,427 | 0,024 | 1,687 | 0,042 | 2,079 | 0,004 |
|  | **G-UDCA** | 8 | 0,0806 | 0,9746 | 8 | 1,0135 | 0,4524 | 8 | 1,8392 | 0,9805 | 8 | 0,8067 | 0,6761 | 24 | 1,2198 | 0,4163 | 0,933 | 0,400 | 1,759 | 0,224 | 0,726 | 0,550 | 1,139 | 0,220 |
|  | **LCA** | 8 | -0,1801 | 0,6291 | 7 | 1,4102 | 0,2728 | 6 | 0,8056 | 0,7436 | 8 | 1,2219 | 0,7684 | 21 | 1,1657 | 0,3586 | 1,590 | 0,046 | 0,986 | 0,330 | 1,402 | 0,180 | 1,346 | 0,064 |
|  | **T-CA** | 8 | 0,0415 | 0,6708 | 8 | -0,7518 | 0,4002 | 8 | 0,0116 | 1,0271 | 8 | -0,5648 | 0,4344 | 24 | -0,4350 | 0,3834 | -0,793 | 0,327 | -0,030 | 0,981 | -0,606 | 0,461 | -0,476 | 0,540 |
|  | **T-CDCA** | 8 | 0,0849 | 0,8193 | 8 | -0,1501 | 0,5584 | 8 | 0,7971 | 0,6131 | 8 | -0,1592 | 0,4071 | 24 | 0,1626 | 0,3088 | -0,235 | 0,816 | 0,712 | 0,498 | -0,244 | 0,793 | 0,078 | 0,913 |
|  | **T-DCA** | 8 | 0,0710 | 0,5823 | 8 | 0,7292 | 0,5175 | 8 | 0,5312 | 0,7866 | 8 | 0,9889 | 0,5555 | 24 | 0,7497 | 0,3504 | 0,658 | 0,412 | 0,460 | 0,645 | 0,918 | 0,273 | 0,679 | 0,336 |
|  | **T-UDCA** | 7 | 0,1201 | 0,7117 | 8 | -0,6537 | 0,6987 | 7 | 0,8100 | 1,0204 | 8 | -1,1671 | 0,6092 | 23 | -0,3868 | 0,4605 | -0,774 | 0,453 | 0,690 | 0,589 | -1,287 | 0,190 | -0,507 | 0,588 |
| **Carbohydrates** | **Arabinose** | 8 | 0,0057 | 0,2320 | 8 | 0,1872 | 0,2448 | 8 | 0,1523 | 0,1987 | 8 | -0,0871 | 0,2321 | 24 | 0,0841 | 0,1273 | 0,182 | 0,599 | 0,147 | 0,639 | -0,093 | 0,782 | 0,078 | 0,763 |
|  | **Arabitol** | 8 | -0,0186 | 0,1669 | 8 | 0,0175 | 0,1715 | 8 | 0,0083 | 0,2206 | 8 | 0,0647 | 0,2966 | 24 | 0,0302 | 0,1299 | 0,036 | 0,882 | 0,027 | 0,924 | 0,083 | 0,810 | 0,049 | 0,844 |
|  | **Erythritol** | 8 | -0,0212 | 0,1505 | 8 | 0,1031 | 0,1959 | 8 | 0,0269 | 0,2092 | 8 | 0,1678 | 0,2642 | 24 | 0,0993 | 0,1247 | 0,124 | 0,623 | 0,048 | 0,855 | 0,189 | 0,544 | 0,120 | 0,611 |
|  | **Fructose** | 8 | -0,0022 | 0,2807 | 8 | 0,6878 | 0,2142 | 8 | 0,4828 | 0,4890 | 8 | 0,5102 | 0,3428 | 24 | 0,5603 | 0,2030 | 0,690 | 0,071 | 0,485 | 0,404 | 0,512 | 0,267 | 0,562 | 0,158 |
|  | **Galactitol;Sorbitol;Mannitol** | 8 | 0,0035 | 0,0836 | 8 | -0,1149 | 0,1195 | 8 | -0,0599 | 0,1005 | 8 | -0,3768 | 0,1189 | 24 | -0,1839 | 0,0688 | -0,118 | 0,431 | -0,063 | 0,635 | -0,380 | 0,020 | -0,187 | 0,158 |
|  | **Glucose** | 8 | 0,0017 | 0,0468 | 8 | -0,0891 | 0,0511 | 8 | -0,0612 | 0,0443 | 8 | -0,2180 | 0,0375 | 24 | -0,1228 | 0,0285 | -0,091 | 0,211 | -0,063 | 0,346 | -0,220 | 0,003 | -0,124 | 0,035 |
|  | **Glucose-6-phosphate** | 8 | 0,1221 | 0,6812 | 8 | 0,6883 | 0,5612 | 8 | 1,5945 | 0,7306 | 8 | 0,9908 | 0,5276 | 24 | 1,0912 | 0,3471 | 0,566 | 0,532 | 1,472 | 0,163 | 0,869 | 0,330 | 0,969 | 0,187 |
|  | **Glucuronic acid** | 8 | 0,0043 | 0,0732 | 8 | 0,1717 | 0,1384 | 8 | 0,2287 | 0,1156 | 8 | 0,0392 | 0,1075 | 24 | 0,1465 | 0,0689 | 0,167 | 0,303 | 0,224 | 0,123 | 0,035 | 0,792 | 0,142 | 0,273 |
|  | **Glyceric acid** | 8 | -0,0323 | 0,0798 | 8 | -0,1907 | 0,1206 | 8 | -0,8432 | 0,2092 | 8 | -1,1602 | 0,0651 | 24 | -0,7313 | 0,1159 | -0,158 | 0,292 | -0,811 | 0,003 | -1,128 | 0,000 | -0,699 | 0,002 |
|  | **Glycerol** | 8 | 0,0038 | 0,1631 | 8 | 0,0308 | 0,3492 | 8 | 0,1809 | 0,2068 | 8 | -0,3498 | 0,1626 | 24 | -0,0460 | 0,1468 | 0,027 | 0,945 | 0,177 | 0,512 | -0,354 | 0,147 | -0,050 | 0,856 |
|  | **Lactose** | 8 | -0,0124 | 0,3483 | 8 | 1,0116 | 0,8355 | 8 | 1,3203 | 0,6395 | 8 | 0,5110 | 0,5870 | 24 | 0,9476 | 0,3900 | 1,024 | 0,277 | 1,333 | 0,089 | 0,523 | 0,456 | 0,960 | 0,186 |
|  | **Maltose;Trehalose** | 8 | -0,0171 | 0,1294 | 8 | 0,2162 | 0,3260 | 8 | -0,2460 | 0,2186 | 8 | -0,2146 | 0,2457 | 24 | -0,0815 | 0,1539 | 0,233 | 0,517 | -0,229 | 0,383 | -0,198 | 0,489 | -0,064 | 0,819 |
|  | **Mannitol** | 8 | 0,3140 | 0,9215 | 8 | 1,6971 | 0,7123 | 8 | 1,2869 | 0,6910 | 8 | 1,6292 | 0,3034 | 24 | 1,5377 | 0,3326 | 1,383 | 0,255 | 0,973 | 0,413 | 1,315 | 0,197 | 1,224 | 0,126 |
|  | **Ribitol** | 8 | -0,0026 | 0,2057 | 8 | 0,0558 | 0,1811 | 8 | 0,3767 | 0,1728 | 8 | 0,3330 | 0,1711 | 24 | 0,2552 | 0,1010 | 0,058 | 0,834 | 0,379 | 0,180 | 0,336 | 0,230 | 0,258 | 0,231 |
|  | **Ribose;Ribulose** | 8 | -0,0104 | 0,1144 | 8 | 0,0789 | 0,1494 | 8 | -0,0563 | 0,2144 | 8 | -0,2805 | 0,1156 | 24 | -0,0860 | 0,0961 | 0,089 | 0,643 | -0,046 | 0,853 | -0,270 | 0,119 | -0,076 | 0,678 |
|  | **Sucrose** | 8 | -0,0332 | 0,5572 | 8 | 2,1670 | 0,6585 | 8 | 0,3114 | 0,6694 | 8 | 1,1413 | 0,6162 | 24 | 1,2066 | 0,3912 | 2,200 | 0,023 | 0,345 | 0,698 | 1,174 | 0,179 | 1,240 | 0,110 |
|  | **Threonic acid** | 8 | -0,0135 | 0,1555 | 8 | 0,3497 | 0,1852 | 8 | 0,1174 | 0,2169 | 8 | 0,0779 | 0,1489 | 24 | 0,1817 | 0,1055 | 0,363 | 0,155 | 0,131 | 0,631 | 0,091 | 0,677 | 0,195 | 0,347 |
|  | **Xylitol** | 8 | 0,0026 | 0,2232 | 8 | 0,0644 | 0,2089 | 8 | 0,3728 | 0,2586 | 8 | 0,2289 | 0,2742 | 24 | 0,2221 | 0,1397 | 0,062 | 0,842 | 0,370 | 0,297 | 0,226 | 0,532 | 0,220 | 0,430 |
|  | **Xylose** | 8 | -0,0112 | 0,6654 | 8 | -0,0025 | 0,6364 | 8 | -0,1467 | 0,2998 | 8 | 0,0501 | 0,5936 | 24 | -0,0330 | 0,2937 | 0,009 | 0,993 | -0,136 | 0,855 | 0,061 | 0,946 | -0,022 | 0,973 |
| **Fatty Acyls** | **3-hydroxyisovaleric acid** | 8 | -0,0226 | 0,0740 | 8 | -0,0639 | 0,0845 | 8 | -0,2309 | 0,0810 | 8 | -0,2571 | 0,0797 | 24 | -0,1840 | 0,0485 | -0,041 | 0,719 | -0,208 | 0,078 | -0,234 | 0,049 | -0,161 | 0,098 |
|  | **3-methylglutarylcarnitine*** | 8 | 0,0935 | 0,3966 | 8 | 0,3535 | 0,2069 | 8 | 0,2741 | 0,3813 | 8 | -0,4180 | 0,1739 | 24 | 0,0699 | 0,1655 | 0,260 | 0,570 | 0,181 | 0,748 | -0,512 | 0,257 | -0,024 | 0,949 |
|  | **Acetylcarnitine** | 8 | 0,0274 | 0,2922 | 8 | -0,2027 | 0,1848 | 8 | -0,0450 | 0,1309 | 8 | 0,0489 | 0,1038 | 24 | -0,0662 | 0,0822 | -0,230 | 0,517 | -0,072 | 0,825 | 0,022 | 0,946 | -0,094 | 0,670 |
|  | **Aminocaproic acid** | 8 | 0,0054 | 0,1404 | 8 | 0,2070 | 0,1060 | 8 | 0,2522 | 0,2055 | 8 | 0,2929 | 0,1089 | 24 | 0,2507 | 0,0817 | 0,202 | 0,271 | 0,247 | 0,338 | 0,288 | 0,128 | 0,245 | 0,143 |
|  | **Arachidic acid** | 8 | -0,0073 | 0,0409 | 8 | -0,0611 | 0,0498 | 8 | -0,0753 | 0,0285 | 8 | -0,1283 | 0,0442 | 24 | -0,0882 | 0,0239 | -0,054 | 0,418 | -0,068 | 0,194 | -0,121 | 0,064 | -0,081 | 0,099 |
|  | **Arachidonic acid** | 8 | 0,0164 | 0,1128 | 8 | -0,1091 | 0,1008 | 8 | 0,4971 | 0,1495 | 8 | 0,2871 | 0,1268 | 24 | 0,2250 | 0,0876 | -0,126 | 0,421 | 0,481 | 0,022 | 0,271 | 0,133 | 0,209 | 0,218 |
|  | **Behenic acid** | 8 | -0,0125 | 0,0715 | 8 | -0,1712 | 0,0794 | 8 | -0,1835 | 0,0599 | 8 | -0,2042 | 0,0906 | 24 | -0,1863 | 0,0429 | -0,159 | 0,160 | -0,171 | 0,088 | -0,192 | 0,119 | -0,174 | 0,050 |
|  | **Capric acid** | 8 | -0,0024 | 0,1253 | 8 | 0,1314 | 0,1532 | 8 | -0,0645 | 0,0991 | 8 | 0,0287 | 0,1085 | 24 | 0,0319 | 0,0696 | 0,134 | 0,510 | -0,062 | 0,703 | 0,031 | 0,854 | 0,034 | 0,809 |
|  | **Caproic acid** | 8 | -0,0051 | 0,0937 | 8 | 0,0340 | 0,0940 | 8 | 0,1779 | 0,0400 | 8 | 0,1663 | 0,0919 | 24 | 0,1260 | 0,0458 | 0,039 | 0,772 | 0,183 | 0,094 | 0,171 | 0,213 | 0,131 | 0,181 |
|  | **Caprylic acid** | 8 | 0,0497 | 0,1758 | 8 | -0,1195 | 0,0354 | 8 | 0,0300 | 0,0627 | 8 | -0,1096 | 0,0848 | 24 | -0,0664 | 0,0382 | -0,169 | 0,361 | -0,020 | 0,917 | -0,159 | 0,428 | -0,116 | 0,336 |
|  | **Carnitine C10:0** | 8 | 0,0091 | 0,2904 | 8 | 0,8790 | 0,4755 | 8 | 0,0130 | 0,4053 | 8 | -0,5613 | 0,2534 | 24 | 0,1103 | 0,2477 | 0,870 | 0,141 | 0,004 | 0,994 | -0,570 | 0,161 | 0,101 | 0,829 |
|  | **Carnitine C10:1** | 8 | 0,0326 | 0,2639 | 8 | 0,5507 | 0,3661 | 8 | -0,0834 | 0,3792 | 8 | -0,4201 | 0,3079 | 24 | 0,0157 | 0,2118 | 0,518 | 0,270 | -0,116 | 0,805 | -0,453 | 0,283 | -0,017 | 0,967 |
|  | **Carnitine C12:0** | 8 | 0,0016 | 0,2859 | 8 | 0,4918 | 0,3980 | 8 | 0,2494 | 0,2160 | 8 | -0,6125 | 0,1940 | 24 | 0,0429 | 0,1854 | 0,490 | 0,334 | 0,248 | 0,500 | -0,614 | 0,097 | 0,041 | 0,910 |
|  | **Carnitine C12:1** | 8 | 0,0007 | 0,3079 | 8 | 0,2380 | 0,3973 | 8 | 0,3707 | 0,3496 | 8 | -0,6419 | 0,2100 | 24 | -0,0111 | 0,2041 | 0,237 | 0,644 | 0,370 | 0,440 | -0,643 | 0,107 | -0,012 | 0,977 |
|  | **Carnitine C14:0(OH)** | 8 | 0,0173 | 0,2101 | 8 | 0,2701 | 0,2207 | 8 | 0,7179 | 0,1558 | 8 | 0,0449 | 0,1957 | 24 | 0,3443 | 0,1212 | 0,253 | 0,421 | 0,701 | 0,018 | 0,028 | 0,925 | 0,327 | 0,188 |
|  | **Carnitine C14:0** | 8 | -0,0012 | 0,2373 | 8 | 0,2095 | 0,3197 | 8 | 0,3952 | 0,1094 | 8 | -0,0108 | 0,1627 | 24 | 0,1980 | 0,1244 | 0,211 | 0,605 | 0,396 | 0,152 | -0,010 | 0,974 | 0,199 | 0,441 |
|  | **Carnitine C14:1** | 8 | 0,0099 | 0,2872 | 8 | 0,3042 | 0,3771 | 8 | 0,6223 | 0,2539 | 8 | -0,6122 | 0,1879 | 24 | 0,1047 | 0,1909 | 0,294 | 0,545 | 0,612 | 0,133 | -0,622 | 0,091 | 0,095 | 0,800 |
|  | **Carnitine C14:2** | 8 | 0,0224 | 0,3130 | 8 | 0,1446 | 0,3328 | 8 | 0,2236 | 0,2981 | 8 | -0,7985 | 0,3169 | 24 | -0,1435 | 0,1995 | 0,122 | 0,793 | 0,201 | 0,649 | -0,821 | 0,087 | -0,166 | 0,674 |
|  | **Carnitine C16:0** | 8 | -0,0082 | 0,1901 | 8 | 0,2295 | 0,2346 | 8 | 0,5130 | 0,0994 | 8 | 0,3015 | 0,2277 | 24 | 0,3480 | 0,1117 | 0,238 | 0,444 | 0,521 | 0,029 | 0,310 | 0,314 | 0,356 | 0,120 |
|  | **Carnitine C16:1** | 8 | 0,0002 | 0,2473 | 8 | 0,1511 | 0,3709 | 8 | 0,8079 | 0,1699 | 8 | -0,2033 | 0,2222 | 24 | 0,2519 | 0,1718 | 0,151 | 0,740 | 0,808 | 0,018 | -0,203 | 0,550 | 0,252 | 0,453 |
|  | **Carnitine C16:2** | 8 | 0,0241 | 0,3439 | 8 | 0,0484 | 0,4726 | 8 | 0,2584 | 0,4040 | 8 | -1,2809 | 0,4805 | 24 | -0,3247 | 0,2878 | 0,024 | 0,967 | 0,234 | 0,665 | -1,305 | 0,044 | -0,349 | 0,523 |
|  | **Carnitine C18:0** | 8 | -0,0201 | 0,2288 | 8 | 0,0520 | 0,2257 | 8 | 0,0020 | 0,1131 | 8 | 0,0532 | 0,2281 | 24 | 0,0357 | 0,1085 | 0,072 | 0,826 | 0,022 | 0,932 | 0,073 | 0,824 | 0,056 | 0,809 |
|  | **Carnitine C18:1** | 8 | -0,0191 | 0,1819 | 8 | 0,1037 | 0,2934 | 8 | 0,5476 | 0,1125 | 8 | 0,1085 | 0,1904 | 24 | 0,2533 | 0,1248 | 0,123 | 0,727 | 0,567 | 0,019 | 0,128 | 0,635 | 0,272 | 0,267 |
|  | **Carnitine C18:2** | 8 | -0,0119 | 0,2281 | 8 | -0,0298 | 0,2075 | 8 | 0,1728 | 0,1250 | 8 | 0,0297 | 0,2762 | 24 | 0,0576 | 0,1184 | -0,018 | 0,954 | 0,185 | 0,489 | 0,042 | 0,909 | 0,069 | 0,777 |
|  | **Carnitine C3:0** | 8 | 0,0089 | 0,2787 | 8 | 0,6858 | 0,1867 | 8 | 0,3051 | 0,2217 | 8 | 0,7240 | 0,2204 | 24 | 0,5716 | 0,1225 | 0,677 | 0,063 | 0,296 | 0,420 | 0,715 | 0,064 | 0,563 | 0,042 |
|  | **Carnitine C4:0(OH)** | 8 | 0,0245 | 0,5814 | 8 | 0,4043 | 0,3040 | 8 | 0,4041 | 0,3137 | 8 | 0,3033 | 0,3725 | 24 | 0,3706 | 0,1831 | 0,380 | 0,572 | 0,380 | 0,575 | 0,279 | 0,692 | 0,346 | 0,454 |
|  | **Carnitine C4:0** | 8 | -0,0076 | 0,1902 | 8 | 1,0635 | 0,4063 | 8 | 0,0180 | 0,2043 | 8 | 0,4883 | 0,2047 | 24 | 0,5233 | 0,1822 | 1,071 | 0,032 | 0,026 | 0,928 | 0,496 | 0,098 | 0,531 | 0,125 |
|  | **Carnitine C5:0** | 8 | 0,0254 | 0,2608 | 8 | 0,3670 | 0,2157 | 8 | -0,0806 | 0,2878 | 8 | 0,8090 | 0,1853 | 24 | 0,3652 | 0,1495 | 0,342 | 0,330 | -0,106 | 0,789 | 0,784 | 0,028 | 0,340 | 0,266 |
|  | **Carnitine C6:0** | 8 | 0,0371 | 0,3130 | 8 | 0,8541 | 0,3400 | 8 | 0,2027 | 0,2774 | 8 | -0,0756 | 0,2789 | 24 | 0,3271 | 0,1845 | 0,817 | 0,099 | 0,166 | 0,698 | -0,113 | 0,792 | 0,290 | 0,436 |
|  | **Carnitine C8:0** | 8 | 0,0138 | 0,2646 | 8 | 0,9431 | 0,4614 | 8 | 0,0637 | 0,3599 | 8 | -0,3646 | 0,2808 | 24 | 0,2141 | 0,2358 | 0,929 | 0,103 | 0,050 | 0,913 | -0,378 | 0,343 | 0,200 | 0,651 |
|  | **Carnitine C8:1** | 8 | 0,0259 | 0,3284 | 8 | 0,6292 | 0,3119 | 8 | 0,4172 | 0,3315 | 8 | -0,0605 | 0,3563 | 24 | 0,3286 | 0,1937 | 0,603 | 0,204 | 0,391 | 0,416 | -0,086 | 0,861 | 0,303 | 0,438 |
|  | **Docosadienoic acid** | 8 | -0,0240 | 0,1845 | 8 | -0,0782 | 0,2088 | 8 | 0,3239 | 0,1304 | 8 | -0,2218 | 0,2291 | 24 | 0,0080 | 0,1174 | -0,054 | 0,848 | 0,348 | 0,146 | -0,198 | 0,512 | 0,032 | 0,891 |
|  | **Docosahexaenoic acid** | 8 | -0,0448 | 0,2290 | 8 | -0,6915 | 0,2213 | 8 | 0,0754 | 0,1150 | 8 | -0,6940 | 0,1341 | 24 | -0,4367 | 0,1176 | -0,647 | 0,062 | 0,120 | 0,646 | -0,649 | 0,028 | -0,392 | 0,116 |
|  | **Docosanedioic acid** | 8 | -0,0070 | 0,0377 | 8 | -0,0281 | 0,0293 | 8 | -0,0664 | 0,0201 | 8 | -0,0061 | 0,0552 | 24 | -0,0335 | 0,0215 | -0,021 | 0,665 | -0,059 | 0,186 | 0,001 | 0,989 | -0,027 | 0,543 |
|  | **Docosapentaenoic acid** | 8 | -0,0477 | 0,2121 | 8 | -0,7180 | 0,3081 | 8 | 0,3925 | 0,2171 | 8 | -0,6918 | 0,1429 | 24 | -0,3391 | 0,1677 | -0,670 | 0,095 | 0,440 | 0,169 | -0,644 | 0,025 | -0,291 | 0,365 |
|  | **Docosatetraenoic acid** | 8 | 0,0103 | 0,1652 | 8 | -0,2007 | 0,2483 | 8 | 0,8875 | 0,1767 | 8 | 0,2314 | 0,1547 | 24 | 0,3061 | 0,1434 | -0,211 | 0,491 | 0,877 | 0,003 | 0,221 | 0,345 | 0,296 | 0,278 |
|  | **Docosatrienoic acid** | 8 | -0,0064 | 0,1803 | 8 | 0,0173 | 0,3016 | 8 | 0,7680 | 0,1706 | 8 | 0,1721 | 0,1927 | 24 | 0,3191 | 0,1432 | 0,024 | 0,947 | 0,774 | 0,008 | 0,179 | 0,510 | 0,326 | 0,238 |
|  | **Docosenoic acid** | 8 | -0,0291 | 0,4482 | 8 | -0,5995 | 0,1277 | 8 | -0,4899 | 0,0742 | 8 | -0,8434 | 0,0879 | 24 | -0,6443 | 0,0628 | -0,570 | 0,241 | -0,461 | 0,328 | -0,814 | 0,096 | -0,615 | 0,032 |
|  | **Dodecenedioic acid** | 8 | 0,0409 | 0,0898 | 8 | 0,1451 | 0,0897 | 8 | -0,0052 | 0,0494 | 8 | 0,2642 | 0,1374 | 24 | 0,1347 | 0,0592 | 0,104 | 0,425 | -0,046 | 0,660 | 0,223 | 0,195 | 0,094 | 0,422 |
|  | **Dodecenoic acid** | 8 | -0,0108 | 0,2237 | 8 | 0,2898 | 0,3100 | 8 | 0,5050 | 0,3475 | 8 | -0,0192 | 0,2365 | 24 | 0,2585 | 0,1723 | 0,301 | 0,445 | 0,516 | 0,232 | -0,008 | 0,980 | 0,269 | 0,416 |
|  | **Eicosadienoic acid** | 8 | -0,0134 | 0,1980 | 8 | -0,1953 | 0,2445 | 8 | 0,5420 | 0,2401 | 8 | -0,2862 | 0,2757 | 24 | 0,0202 | 0,1600 | -0,182 | 0,572 | 0,555 | 0,096 | -0,273 | 0,435 | 0,034 | 0,912 |
|  | **Eicosapentaenoic acid** | 8 | -0,0393 | 0,1621 | 8 | -0,3596 | 0,0776 | 8 | -0,1776 | 0,1036 | 8 | -0,4500 | 0,0535 | 24 | -0,3291 | 0,0505 | -0,320 | 0,096 | -0,138 | 0,484 | -0,411 | 0,031 | -0,290 | 0,029 |
|  | **Eicosatrienoic acid** | 8 | -0,0120 | 0,1442 | 8 | -0,6245 | 0,1734 | 8 | 0,3765 | 0,2421 | 8 | -0,2048 | 0,1112 | 24 | -0,1509 | 0,1326 | -0,613 | 0,017 | 0,389 | 0,190 | -0,193 | 0,307 | -0,139 | 0,576 |
|  | **Eicosenoic acid** | 8 | -0,0261 | 0,2793 | 8 | -0,1352 | 0,2806 | 8 | 0,4412 | 0,2998 | 8 | -0,4560 | 0,2472 | 24 | -0,0500 | 0,1712 | -0,109 | 0,787 | 0,467 | 0,273 | -0,430 | 0,268 | -0,024 | 0,944 |
|  | **Etherolenic acid** | 8 | 0,0019 | 0,0674 | 8 | -0,1517 | 0,0725 | 8 | -0,1420 | 0,1394 | 8 | -0,2572 | 0,0854 | 24 | -0,1837 | 0,0580 | -0,154 | 0,143 | -0,144 | 0,368 | -0,259 | 0,032 | -0,186 | 0,097 |
|  | **Glutarylcarnitine** | 8 | 0,1740 | 0,1424 | 8 | 0,5419 | 0,1017 | 8 | 0,3558 | 0,1475 | 8 | 0,2551 | 0,1573 | 24 | 0,3843 | 0,0799 | 0,368 | 0,054 | 0,182 | 0,390 | 0,081 | 0,708 | 0,210 | 0,201 |
|  | **Heptadecatrienoic acid** | 8 | 0,0235 | 0,0333 | 8 | -0,0475 | 0,0887 | 8 | 0,0266 | 0,0568 | 8 | -0,0859 | 0,0320 | 24 | -0,0356 | 0,0364 | -0,071 | 0,466 | 0,003 | 0,964 | -0,109 | 0,033 | -0,059 | 0,380 |
|  | **Heptadecenoic acid** | 8 | -0,0061 | 0,1295 | 8 | -0,0411 | 0,1693 | 8 | 0,4713 | 0,2258 | 8 | -0,1831 | 0,1802 | 24 | 0,0824 | 0,1217 | -0,035 | 0,872 | 0,477 | 0,088 | -0,177 | 0,438 | 0,088 | 0,697 |
|  | **Hexacosanoic acid** | 8 | 0,0005 | 0,0786 | 8 | -0,0782 | 0,0668 | 8 | -0,1149 | 0,0434 | 8 | -0,1458 | 0,0529 | 24 | -0,1130 | 0,0310 | -0,079 | 0,458 | -0,115 | 0,219 | -0,146 | 0,145 | -0,114 | 0,114 |
|  | **Hexadecadienoic acid** | 8 | 0,0170 | 0,1818 | 8 | -0,0750 | 0,2253 | 8 | 0,3960 | 0,2403 | 8 | -0,1184 | 0,2229 | 24 | 0,0675 | 0,1357 | -0,092 | 0,755 | 0,379 | 0,229 | -0,135 | 0,645 | 0,051 | 0,846 |
|  | **Hexadecanedioic acid** | 8 | -0,0034 | 0,0182 | 8 | 0,0202 | 0,0212 | 8 | -0,0143 | 0,0193 | 8 | 0,0137 | 0,0305 | 24 | 0,0065 | 0,0137 | 0,024 | 0,412 | -0,011 | 0,690 | 0,017 | 0,637 | 0,010 | 0,704 |
|  | **Hydroxydodecanoic acid*** | 8 | -0,0017 | 0,0221 | 8 | -0,0073 | 0,0170 | 8 | -0,0143 | 0,0270 | 8 | -0,0698 | 0,0284 | 24 | -0,0305 | 0,0148 | -0,006 | 0,845 | -0,013 | 0,724 | -0,068 | 0,079 | -0,029 | 0,325 |
|  | **Lauric acid** | 8 | -0,0003 | 0,0656 | 8 | 0,0929 | 0,1048 | 8 | 0,0778 | 0,1063 | 8 | -0,0109 | 0,0768 | 24 | 0,0533 | 0,0543 | 0,093 | 0,463 | 0,078 | 0,542 | -0,011 | 0,918 | 0,054 | 0,603 |
|  | **Linoleic acid** | 8 | 0,0200 | 0,2674 | 8 | 0,0216 | 0,3287 | 8 | 0,4900 | 0,4090 | 8 | -0,3581 | 0,2939 | 24 | 0,0512 | 0,2048 | 0,002 | 0,997 | 0,470 | 0,352 | -0,378 | 0,357 | 0,031 | 0,937 |
|  | **Linolenic acid** | 8 | 0,0523 | 0,3105 | 8 | -0,8205 | 0,3098 | 8 | -0,2278 | 0,5452 | 8 | -0,9994 | 0,2629 | 24 | -0,6826 | 0,2272 | -0,873 | 0,066 | -0,280 | 0,662 | -1,052 | 0,022 | -0,735 | 0,101 |
|  | **Myristic acid** | 8 | -0,0132 | 0,0994 | 8 | 0,0224 | 0,1143 | 8 | 0,2282 | 0,1562 | 8 | -0,0902 | 0,1410 | 24 | 0,0535 | 0,0811 | 0,036 | 0,818 | 0,241 | 0,213 | -0,077 | 0,662 | 0,067 | 0,665 |
|  | **Myristoleic acid** | 8 | -0,0142 | 0,1691 | 8 | 0,2333 | 0,2738 | 8 | 0,5443 | 0,2627 | 8 | -0,0212 | 0,2125 | 24 | 0,2522 | 0,1467 | 0,247 | 0,455 | 0,559 | 0,095 | -0,007 | 0,980 | 0,266 | 0,338 |
|  | **Nonadecanoic acid** | 8 | -0,0286 | 0,0643 | 8 | -0,0767 | 0,0619 | 8 | -0,0177 | 0,0397 | 8 | -0,1852 | 0,0886 | 24 | -0,0932 | 0,0394 | -0,048 | 0,599 | 0,011 | 0,887 | -0,157 | 0,175 | -0,065 | 0,413 |
|  | **Nonadecenoic acid** | 8 | 0,0071 | 0,0872 | 8 | -0,0385 | 0,1114 | 8 | 0,2470 | 0,1215 | 8 | -0,1215 | 0,1137 | 24 | 0,0290 | 0,0718 | -0,046 | 0,752 | 0,240 | 0,131 | -0,129 | 0,385 | 0,022 | 0,872 |
|  | **Nonanoic acid** | 8 | 0,0245 | 0,0575 | 8 | -0,0406 | 0,0268 | 8 | -0,0496 | 0,0247 | 8 | -0,0975 | 0,0182 | 24 | -0,0626 | 0,0140 | -0,065 | 0,322 | -0,074 | 0,256 | -0,122 | 0,063 | -0,087 | 0,039 |
|  | **Oleic acid;Elaidic acid;Vaccenic acid** | 8 | 0,0032 | 0,2075 | 8 | -0,0480 | 0,2487 | 8 | 0,4605 | 0,3054 | 8 | -0,3250 | 0,2714 | 24 | 0,0292 | 0,1668 | -0,051 | 0,877 | 0,457 | 0,236 | -0,328 | 0,353 | 0,026 | 0,935 |
|  | **Palmitic acid** | 8 | -0,0098 | 0,0722 | 8 | -0,0334 | 0,1144 | 8 | 0,1628 | 0,0975 | 8 | -0,1368 | 0,1152 | 24 | -0,0024 | 0,0656 | -0,024 | 0,864 | 0,173 | 0,177 | -0,127 | 0,366 | 0,007 | 0,953 |
|  | **Palmitoleic acid** | 8 | 0,0198 | 0,3321 | 8 | -0,1151 | 0,4057 | 8 | 0,9423 | 0,4626 | 8 | -0,1230 | 0,3835 | 24 | 0,2348 | 0,2534 | -0,135 | 0,801 | 0,923 | 0,128 | -0,143 | 0,782 | 0,215 | 0,658 |
|  | **Pentadecanoic acid** | 8 | -0,0207 | 0,0743 | 8 | -0,0068 | 0,0866 | 8 | 0,1288 | 0,1208 | 8 | -0,2009 | 0,1025 | 24 | -0,0263 | 0,0641 | 0,014 | 0,905 | 0,150 | 0,310 | -0,180 | 0,177 | -0,006 | 0,963 |
|  | **Pentadecenoic acid** | 8 | -0,0114 | 0,0486 | 8 | -0,0237 | 0,0645 | 8 | 0,0726 | 0,0601 | 8 | -0,1560 | 0,0406 | 24 | -0,0357 | 0,0366 | -0,012 | 0,882 | 0,084 | 0,295 | -0,145 | 0,038 | -0,024 | 0,730 |
|  | **Sebacic acid** | 8 | -0,0017 | 0,0294 | 8 | 0,0734 | 0,0326 | 8 | 0,0392 | 0,0333 | 8 | -0,0056 | 0,0259 | 24 | 0,0356 | 0,0183 | 0,075 | 0,109 | 0,041 | 0,373 | -0,004 | 0,921 | 0,037 | 0,307 |
|  | **Stearic acid** | 8 | -0,0058 | 0,0308 | 8 | -0,0738 | 0,0474 | 8 | -0,0295 | 0,0176 | 8 | -0,1163 | 0,0481 | 24 | -0,0732 | 0,0234 | -0,068 | 0,248 | -0,024 | 0,515 | -0,111 | 0,074 | -0,067 | 0,140 |
|  | **Stearidonic acid** | 8 | -0,0220 | 0,3178 | 8 | -0,4999 | 0,1744 | 8 | -0,1515 | 0,2336 | 8 | -0,6197 | 0,1092 | 24 | -0,4237 | 0,1074 | -0,478 | 0,209 | -0,130 | 0,748 | -0,598 | 0,097 | -0,402 | 0,131 |
|  | **Tetracosenoic acid** | 8 | -0,0514 | 0,3372 | 8 | -0,2712 | 0,2289 | 8 | -0,1362 | 0,1183 | 8 | -0,4053 | 0,1098 | 24 | -0,2709 | 0,0921 | -0,220 | 0,598 | -0,085 | 0,816 | -0,354 | 0,335 | -0,219 | 0,383 |
|  | **Tetradecadienoic acid** | 8 | -0,0013 | 0,2017 | 8 | 0,1996 | 0,4121 | 7 | 0,2817 | 0,3890 | 8 | -0,2189 | 0,2788 | 23 | 0,0790 | 0,2053 | 0,201 | 0,668 | 0,283 | 0,514 | -0,218 | 0,537 | 0,080 | 0,830 |
|  | **Tetradecanedioic acid** | 8 | -0,0182 | 0,1070 | 8 | -0,2220 | 0,0371 | 8 | -0,2755 | 0,0307 | 8 | -0,3116 | 0,0211 | 24 | -0,2697 | 0,0184 | -0,204 | 0,093 | -0,257 | 0,037 | -0,293 | 0,018 | -0,252 | 0,001 |
|  | **Undecanedioic acid** | 8 | -0,0054 | 0,0260 | 8 | 0,0266 | 0,0085 | 8 | -0,0185 | 0,0270 | 8 | -0,0418 | 0,0200 | 24 | -0,0112 | 0,0125 | 0,032 | 0,262 | -0,013 | 0,730 | -0,036 | 0,286 | -0,006 | 0,825 |
|  | **Undecanoic acid** | 8 | 0,0248 | 0,0638 | 8 | -0,0152 | 0,0281 | 8 | -0,0631 | 0,0317 | 8 | -0,0653 | 0,0269 | 24 | -0,0478 | 0,0167 | -0,040 | 0,575 | -0,088 | 0,238 | -0,090 | 0,214 | -0,073 | 0,125 |
| **Lipids** | **Glycerol-3-phosphate** | 8 | -0,0059 | 0,1633 | 8 | -0,2448 | 0,1099 | 8 | -0,3803 | 0,2640 | 8 | -0,7542 | 0,2622 | 24 | -0,4598 | 0,1315 | -0,239 | 0,245 | -0,374 | 0,248 | -0,748 | 0,030 | -0,454 | 0,077 |
|  | **Glycerophosphorylcholine** | 8 | 0,0079 | 0,2302 | 7 | -1,2328 | 0,2720 | 6 | -0,4602 | 0,3725 | 8 | -0,5668 | 0,2147 | 21 | -0,7583 | 0,1708 | -1,241 | 0,004 | -0,468 | 0,283 | -0,575 | 0,089 | -0,766 | 0,021 |
|  | **PCae(12:0)** | 7 | -0,0243 | 0,2561 | 8 | -0,7001 | 0,3278 | 8 | -0,4782 | 0,4545 | 7 | -0,2279 | 0,5547 | 23 | -0,4792 | 0,2489 | -0,676 | 0,136 | -0,454 | 0,418 | -0,204 | 0,745 | -0,455 | 0,348 |
|  | **PCae(14:0)** | 8 | -0,0044 | 0,2112 | 8 | -0,1885 | 0,1928 | 8 | 0,2158 | 0,3000 | 8 | 0,0251 | 0,2887 | 24 | 0,0175 | 0,1501 | -0,184 | 0,530 | 0,220 | 0,558 | 0,030 | 0,935 | 0,022 | 0,940 |
|  | **PCae(15:0)** | 8 | -0,0289 | 0,1864 | 8 | -0,2271 | 0,1720 | 8 | 0,0983 | 0,2221 | 8 | -0,1546 | 0,2302 | 24 | -0,0945 | 0,1193 | -0,198 | 0,448 | 0,127 | 0,668 | -0,126 | 0,678 | -0,066 | 0,780 |
|  | **PCae(16:0)** | 8 | -0,0202 | 0,1354 | 8 | -0,1919 | 0,1003 | 8 | 0,1377 | 0,1542 | 8 | 0,0039 | 0,1432 | 24 | -0,0168 | 0,0794 | -0,172 | 0,325 | 0,158 | 0,454 | 0,024 | 0,905 | 0,003 | 0,983 |
|  | **PCae(16:1)** | 8 | -0,0389 | 0,2180 | 8 | -0,2198 | 0,1592 | 8 | 0,5592 | 0,2735 | 8 | 0,3823 | 0,2851 | 24 | 0,2406 | 0,1524 | -0,181 | 0,514 | 0,598 | 0,109 | 0,421 | 0,260 | 0,279 | 0,348 |
|  | **PCae(17:0)** | 8 | -0,0375 | 0,1510 | 8 | -0,4348 | 0,1445 | 8 | -0,1827 | 0,1835 | 8 | -0,3101 | 0,1906 | 24 | -0,3092 | 0,0984 | -0,397 | 0,078 | -0,145 | 0,551 | -0,273 | 0,281 | -0,272 | 0,167 |
|  | **PCae(18:0)** | 8 | 0,0604 | 0,1910 | 8 | -0,4282 | 0,1137 | 8 | -0,0809 | 0,1175 | 8 | -0,2333 | 0,1402 | 24 | -0,2475 | 0,0747 | -0,489 | 0,045 | -0,141 | 0,539 | -0,294 | 0,235 | -0,308 | 0,078 |
|  | **PCae(18:1)** | 8 | -0,0517 | 0,2009 | 8 | -0,4373 | 0,0859 | 8 | 0,0068 | 0,1989 | 8 | -0,0882 | 0,1417 | 24 | -0,1729 | 0,0916 | -0,386 | 0,099 | 0,059 | 0,839 | -0,036 | 0,884 | -0,121 | 0,540 |
|  | **PCae(18:2)** | 8 | -0,0476 | 0,1948 | 8 | -0,7437 | 0,1484 | 8 | -0,5068 | 0,1597 | 8 | -0,5804 | 0,2016 | 24 | -0,6103 | 0,0968 | -0,696 | 0,013 | -0,459 | 0,090 | -0,533 | 0,078 | -0,563 | 0,009 |
|  | **PCae(18:3)** | 8 | -0,0535 | 0,2751 | 8 | -0,9117 | 0,1727 | 8 | -0,3554 | 0,2382 | 8 | -0,2410 | 0,2333 | 24 | -0,5027 | 0,1343 | -0,858 | 0,019 | -0,302 | 0,421 | -0,188 | 0,611 | -0,449 | 0,120 |
|  | **PCae(20:0)** | 8 | -0,0273 | 0,1511 | 8 | -0,4855 | 0,1366 | 8 | -0,3586 | 0,1268 | 8 | -0,3235 | 0,1624 | 24 | -0,3892 | 0,0801 | -0,458 | 0,041 | -0,331 | 0,115 | -0,296 | 0,203 | -0,362 | 0,035 |
|  | **PCae(20:1)** | 8 | 0,0343 | 0,2623 | 8 | -0,5744 | 0,0702 | 8 | -0,1194 | 0,1153 | 8 | -0,4346 | 0,0949 | 24 | -0,3761 | 0,0659 | -0,609 | 0,042 | -0,154 | 0,600 | -0,469 | 0,115 | -0,410 | 0,035 |
|  | **PCae(20:2)** | 8 | -0,0530 | 0,2107 | 8 | -0,6843 | 0,1052 | 8 | -0,1658 | 0,1442 | 8 | -0,3683 | 0,1702 | 24 | -0,4061 | 0,0903 | -0,631 | 0,018 | -0,113 | 0,666 | -0,315 | 0,264 | -0,353 | 0,083 |
|  | **PCae(20:3)** | 8 | -0,0656 | 0,2839 | 8 | -0,3314 | 0,1385 | 8 | 0,3746 | 0,3209 | 8 | 0,2292 | 0,2287 | 24 | 0,0908 | 0,1474 | -0,266 | 0,414 | 0,440 | 0,322 | 0,295 | 0,432 | 0,156 | 0,609 |
|  | **PCae(20:4)** | 8 | -0,0128 | 0,1826 | 8 | -0,4838 | 0,1451 | 8 | 0,3221 | 0,1963 | 8 | 0,0329 | 0,1806 | 24 | -0,0429 | 0,1191 | -0,471 | 0,063 | 0,335 | 0,232 | 0,046 | 0,861 | -0,030 | 0,898 |
|  | **PCae(22:1)** | 8 | -0,1183 | 0,5606 | 8 | -1,3908 | 0,2608 | 8 | -0,9734 | 0,5043 | 8 | -1,0932 | 0,4022 | 24 | -1,1524 | 0,2246 | -1,272 | 0,059 | -0,855 | 0,276 | -0,975 | 0,179 | -1,034 | 0,048 |
|  | **PCae(22:4)** | 8 | -0,0267 | 0,1957 | 8 | -0,3479 | 0,1376 | 8 | 0,4848 | 0,1873 | 8 | 0,4378 | 0,2248 | 24 | 0,1916 | 0,1302 | -0,321 | 0,201 | 0,512 | 0,080 | 0,465 | 0,141 | 0,218 | 0,395 |
|  | **PCae(22:5)** | 6 | 0,0154 | 0,2430 | 7 | -0,5492 | 0,2216 | 8 | 0,2873 | 0,2218 | 7 | -0,2639 | 0,2376 | 22 | -0,1542 | 0,1466 | -0,565 | 0,114 | 0,272 | 0,428 | -0,279 | 0,431 | -0,170 | 0,588 |
|  | **PCae(22:6)** | 8 | -0,0502 | 0,2428 | 8 | -0,6946 | 0,1909 | 8 | -0,0231 | 0,1944 | 8 | -0,5489 | 0,1759 | 24 | -0,4222 | 0,1195 | -0,644 | 0,056 | 0,027 | 0,932 | -0,499 | 0,118 | -0,372 | 0,146 |
|  | **PCee(16:0)** | 8 | -0,0281 | 0,1188 | 8 | -0,4224 | 0,1484 | 8 | -0,1819 | 0,1006 | 8 | -0,1313 | 0,1682 | 24 | -0,2452 | 0,0827 | -0,394 | 0,057 | -0,154 | 0,340 | -0,103 | 0,624 | -0,217 | 0,183 |
|  | **Sphingosine-1-phosphate** | 8 | 0,0141 | 0,0450 | 8 | -0,1181 | 0,0934 | 8 | 0,1105 | 0,0988 | 8 | 0,0890 | 0,1113 | 24 | 0,0271 | 0,0599 | -0,132 | 0,223 | 0,096 | 0,389 | 0,075 | 0,543 | 0,013 | 0,904 |
| **Nucleosides** | **Adenosine;2-deoxyguanosine** | 8 | 0,0013 | 0,2218 | 8 | 0,2533 | 0,0699 | 8 | 0,1893 | 0,1022 | 8 | 0,4297 | 0,1976 | 24 | 0,2908 | 0,0772 | 0,252 | 0,297 | 0,188 | 0,454 | 0,428 | 0,171 | 0,289 | 0,125 |
|  | **Guanosine** | 8 | -0,0516 | 0,2456 | 8 | -0,2866 | 0,1838 | 8 | -0,1519 | 0,1804 | 8 | 0,8476 | 0,3410 | 24 | 0,1364 | 0,1722 | -0,235 | 0,456 | -0,100 | 0,747 | 0,899 | 0,050 | 0,188 | 0,575 |
|  | **S-adenosylhomocysteine** | 8 | 0,0217 | 0,2747 | 8 | 0,7361 | 0,3778 | 8 | 0,6197 | 0,2254 | 8 | 0,5456 | 0,2071 | 24 | 0,6338 | 0,1557 | 0,714 | 0,148 | 0,598 | 0,115 | 0,524 | 0,150 | 0,612 | 0,060 |
|  | **Succinyladenosine** | 8 | -0,0067 | 0,1490 | 8 | 0,2229 | 0,1552 | 8 | 0,1461 | 0,2252 | 8 | 0,0573 | 0,1068 | 24 | 0,1421 | 0,0946 | 0,230 | 0,304 | 0,153 | 0,581 | 0,064 | 0,733 | 0,149 | 0,428 |
|  | **Uridine** | 8 | 0,0136 | 0,1124 | 8 | -0,0252 | 0,0912 | 8 | -0,1290 | 0,1237 | 8 | -0,0716 | 0,0895 | 24 | -0,0753 | 0,0573 | -0,039 | 0,792 | -0,143 | 0,408 | -0,085 | 0,563 | -0,089 | 0,459 |
|  | **UMP** | 8 | 0,1404 | 0,3762 | 8 | 0,7177 | 0,4446 | 8 | 0,3582 | 0,3901 | 8 | 0,5309 | 0,6969 | 24 | 0,5356 | 0,2927 | 0,577 | 0,338 | 0,218 | 0,694 | 0,391 | 0,630 | 0,395 | 0,481 |
| **Organic acids** | **2-hydroxybutyric acid** | 8 | -0,0075 | 0,1819 | 8 | -0,3254 | 0,2072 | 8 | -0,1019 | 0,1206 | 8 | -0,0338 | 0,1492 | 24 | -0,1537 | 0,0936 | -0,318 | 0,268 | -0,094 | 0,672 | -0,026 | 0,913 | -0,146 | 0,454 |
|  | **2-hydroxyglutaric acid** | 8 | -0,0143 | 0,0882 | 8 | -0,0791 | 0,1190 | 8 | -0,2336 | 0,1209 | 8 | 0,1474 | 0,1538 | 24 | -0,0551 | 0,0799 | -0,065 | 0,668 | -0,219 | 0,165 | 0,162 | 0,377 | -0,041 | 0,785 |
|  | **2-oxoglutaric acid** | 8 | -0,0285 | 0,1754 | 8 | 0,0768 | 0,3457 | 8 | 0,4948 | 0,3176 | 8 | 0,9795 | 0,1128 | 24 | 0,5170 | 0,1719 | 0,105 | 0,790 | 0,523 | 0,171 | 1,008 | 0,000 | 0,546 | 0,095 |
|  | **3-hydroxybutyric acid** | 8 | -0,0234 | 0,1879 | 8 | -0,2890 | 0,0399 | 8 | -0,2016 | 0,0698 | 8 | -0,1199 | 0,0334 | 24 | -0,2035 | 0,0313 | -0,266 | 0,188 | -0,178 | 0,389 | -0,097 | 0,621 | -0,180 | 0,138 |
|  | **3-hydroxyglutaric acid** | 8 | -0,0205 | 0,0978 | 8 | 0,0559 | 0,0625 | 8 | -0,0691 | 0,1200 | 8 | 0,1465 | 0,1266 | 24 | 0,0445 | 0,0618 | 0,076 | 0,521 | -0,049 | 0,758 | 0,167 | 0,314 | 0,065 | 0,596 |
|  | **3-indoxyl sulfuric acid** | 8 | 0,0127 | 0,1967 | 8 | 0,7911 | 0,5731 | 8 | -0,1902 | 0,1915 | 8 | 0,0338 | 0,4199 | 24 | 0,2116 | 0,2502 | 0,778 | 0,220 | -0,203 | 0,472 | 0,021 | 0,964 | 0,199 | 0,663 |
|  | **Azelaic acid** | 8 | -0,0011 | 0,0393 | 8 | -0,0207 | 0,0260 | 8 | -0,0286 | 0,0388 | 8 | -0,0680 | 0,0338 | 24 | -0,0391 | 0,0188 | -0,020 | 0,683 | -0,028 | 0,626 | -0,067 | 0,218 | -0,038 | 0,345 |
|  | **Carnitine C14:1(OH)** | 6 | -0,0950 | 0,4240 | 8 | 0,6404 | 0,2858 | 8 | 0,6952 | 0,4347 | 6 | -0,4663 | 0,5106 | 22 | 0,3585 | 0,2488 | 0,735 | 0,161 | 0,790 | 0,229 | -0,371 | 0,588 | 0,454 | 0,397 |
|  | **Carnitine** | 8 | 0,0365 | 0,1714 | 8 | 0,1141 | 0,0821 | 8 | 0,1552 | 0,0998 | 8 | 0,2208 | 0,0781 | 24 | 0,1633 | 0,0490 | 0,078 | 0,689 | 0,119 | 0,559 | 0,184 | 0,345 | 0,127 | 0,331 |
|  | **Citric acid;Isocitric acid** | 8 | -0,0092 | 0,0623 | 8 | -0,1223 | 0,0818 | 8 | -0,1273 | 0,1987 | 8 | -0,2528 | 0,0786 | 24 | -0,1675 | 0,0739 | -0,113 | 0,290 | -0,118 | 0,580 | -0,244 | 0,029 | -0,158 | 0,247 |
|  | **Desaminotyrosine** | 8 | -0,0189 | 0,3160 | 8 | -0,0475 | 0,3263 | 8 | 0,1181 | 0,1792 | 8 | -0,1168 | 0,2148 | 24 | -0,0154 | 0,1384 | -0,029 | 0,951 | 0,137 | 0,712 | -0,098 | 0,801 | 0,003 | 0,991 |
|  | **Fumaric acid** | 8 | -0,0149 | 0,1118 | 8 | 0,0417 | 0,1190 | 8 | 0,0709 | 0,0949 | 8 | 0,0970 | 0,1005 | 24 | 0,0699 | 0,0583 | 0,057 | 0,734 | 0,086 | 0,568 | 0,112 | 0,469 | 0,085 | 0,484 |
|  | **Hypotaurine** | 8 | -0,0361 | 0,1847 | 8 | 0,1159 | 0,1085 | 8 | 0,0070 | 0,2934 | 8 | 0,0724 | 0,1747 | 24 | 0,0651 | 0,1145 | 0,152 | 0,490 | 0,043 | 0,903 | 0,109 | 0,676 | 0,101 | 0,657 |
|  | **Ketoisocaproic acid** | 8 | -0,0223 | 0,1675 | 8 | 0,0878 | 0,1975 | 8 | 0,1488 | 0,2186 | 8 | 0,3021 | 0,0802 | 24 | 0,1796 | 0,0991 | 0,110 | 0,677 | 0,171 | 0,545 | 0,324 | 0,103 | 0,202 | 0,314 |
|  | **Ketoisovaleric acid** | 8 | -0,0234 | 0,1170 | 8 | 0,1026 | 0,2184 | 8 | 0,2108 | 0,2566 | 8 | 0,6125 | 0,0459 | 24 | 0,3086 | 0,1176 | 0,126 | 0,619 | 0,234 | 0,420 | 0,636 | 0,000 | 0,332 | 0,135 |
|  | **Lactic acid** | 8 | -0,0288 | 0,1041 | 8 | 0,1346 | 0,1733 | 8 | -0,1227 | 0,1851 | 8 | 0,3035 | 0,1014 | 24 | 0,1051 | 0,0943 | 0,163 | 0,432 | -0,094 | 0,665 | 0,332 | 0,038 | 0,134 | 0,450 |
|  | **Malic acid** | 8 | -0,0280 | 0,1271 | 8 | 0,3042 | 0,1363 | 8 | 0,0478 | 0,1691 | 8 | 0,5646 | 0,1051 | 24 | 0,3055 | 0,0886 | 0,332 | 0,096 | 0,076 | 0,726 | 0,593 | 0,003 | 0,334 | 0,060 |
|  | **Mandelic acid** | 8 | 0,0512 | 0,2480 | 8 | -0,0756 | 0,2627 | 8 | 0,0148 | 0,1855 | 8 | 0,2512 | 0,1228 | 24 | 0,0634 | 0,1133 | -0,127 | 0,731 | -0,036 | 0,908 | 0,200 | 0,482 | 0,012 | 0,960 |
|  | **Methylmalonic acid** | 8 | -0,0101 | 0,0860 | 8 | 0,0032 | 0,1015 | 8 | 0,1681 | 0,1219 | 8 | 0,0160 | 0,0805 | 24 | 0,0624 | 0,0588 | 0,013 | 0,922 | 0,178 | 0,252 | 0,026 | 0,828 | 0,073 | 0,528 |
|  | **Methylsuccinic acid** | 8 | -0,0601 | 0,1409 | 8 | 0,7031 | 0,3161 | 8 | 0,1602 | 0,1865 | 8 | 0,2059 | 0,2426 | 24 | 0,3564 | 0,1492 | 0,763 | 0,045 | 0,220 | 0,362 | 0,266 | 0,359 | 0,417 | 0,137 |
|  | **N-acetylputrescine** | 8 | 0,0407 | 0,1726 | 8 | -0,0739 | 0,1425 | 8 | 0,0868 | 0,1193 | 8 | -0,1223 | 0,1873 | 24 | -0,0364 | 0,0861 | -0,115 | 0,617 | 0,046 | 0,829 | -0,163 | 0,533 | -0,077 | 0,670 |
|  | **O-phosphoethanolamine** | 8 | -0,0054 | 0,0331 | 8 | -0,2593 | 0,1168 | 8 | -0,4852 | 0,1037 | 8 | -0,6150 | 0,1364 | 24 | -0,4532 | 0,0728 | -0,254 | 0,055 | -0,480 | 0,001 | -0,610 | 0,001 | -0,448 | 0,002 |
|  | **Orotic acid** | 8 | -0,1150 | 0,4062 | 8 | 0,3275 | 0,4150 | 8 | 0,8276 | 0,7778 | 8 | 1,5751 | 0,4110 | 24 | 0,9101 | 0,3277 | 0,443 | 0,459 | 0,943 | 0,301 | 1,690 | 0,011 | 1,025 | 0,107 |
|  | **Phosphoenolpyruvic acid** | 8 | 0,0029 | 0,1947 | 8 | 0,4140 | 0,1737 | 8 | 0,4096 | 0,1745 | 8 | 0,4699 | 0,1897 | 24 | 0,4312 | 0,0991 | 0,411 | 0,137 | 0,407 | 0,142 | 0,467 | 0,108 | 0,428 | 0,045 |
|  | **Pyruvic acid;Oxaloacetic acid** | 8 | -0,0457 | 0,2591 | 8 | 0,3994 | 0,3197 | 8 | 0,7792 | 0,4223 | 8 | 1,8967 | 0,1163 | 24 | 1,0251 | 0,2177 | 0,445 | 0,298 | 0,825 | 0,118 | 1,942 | 0,000 | 1,071 | 0,013 |
|  | **Quinolinic acid*** | 6 | 0,1616 | 0,5393 | 8 | -0,3340 | 0,5777 | 6 | 0,2008 | 0,5407 | 8 | -0,2430 | 0,2557 | 22 | -0,1550 | 0,2640 | -0,496 | 0,555 | 0,039 | 0,960 | -0,405 | 0,475 | -0,317 | 0,588 |
|  | **Taurine** | 8 | 0,0144 | 0,1427 | 8 | -0,2559 | 0,1678 | 8 | -0,3422 | 0,1348 | 8 | -0,4450 | 0,1868 | 24 | -0,3477 | 0,0922 | -0,270 | 0,240 | -0,357 | 0,091 | -0,459 | 0,071 | -0,362 | 0,053 |
|  | **Urea** | 8 | -0,0117 | 0,1557 | 8 | 0,1311 | 0,1593 | 8 | 0,0439 | 0,1371 | 8 | 0,0046 | 0,1665 | 24 | 0,0599 | 0,0861 | 0,143 | 0,532 | 0,056 | 0,793 | 0,016 | 0,944 | 0,072 | 0,684 |
| **Unclassified** | **3-phenylpropionic acid** | 8 | -0,0423 | 0,5613 | 7 | -0,2370 | 0,6117 | 7 | -0,3603 | 0,4531 | 7 | -0,0860 | 0,5856 | 21 | -0,2278 | 0,3047 | -0,195 | 0,818 | -0,318 | 0,673 | -0,044 | 0,958 | -0,185 | 0,760 |
|  | **Acetylcholine** | 8 | 0,0061 | 0,0944 | 8 | 0,0673 | 0,0391 | 8 | -0,0588 | 0,1038 | 8 | -0,0308 | 0,1794 | 24 | -0,0074 | 0,0681 | 0,061 | 0,558 | -0,065 | 0,651 | -0,037 | 0,858 | -0,013 | 0,918 |
|  | **Ascorbic acid** | 8 | 0,0009 | 0,0686 | 8 | 0,6934 | 0,2194 | 8 | -0,2633 | 0,3395 | 8 | 0,9313 | 0,2946 | 24 | 0,4538 | 0,1923 | 0,693 | 0,009 | -0,264 | 0,458 | 0,930 | 0,008 | 0,453 | 0,191 |
|  | **Glycylglycine** | 8 | 0,0202 | 0,1205 | 7 | -0,2387 | 0,1662 | 7 | 0,1052 | 0,1265 | 6 | -0,2536 | 0,1199 | 20 | -0,1228 | 0,0862 | -0,259 | 0,221 | 0,085 | 0,635 | -0,274 | 0,141 | -0,143 | 0,369 |
|  | **Hippuric acid** | 8 | -0,0917 | 0,7259 | 8 | -0,4558 | 0,2648 | 8 | 0,4548 | 0,2620 | 8 | -0,1250 | 0,6703 | 24 | -0,0420 | 0,2566 | -0,364 | 0,645 | 0,547 | 0,490 | -0,033 | 0,974 | 0,050 | 0,935 |
|  | **Homovanillic acid** | 8 | 0,0033 | 0,2432 | 8 | 0,1177 | 0,1230 | 8 | 0,1556 | 0,1925 | 8 | 0,2473 | 0,2238 | 24 | 0,1736 | 0,1025 | 0,114 | 0,681 | 0,152 | 0,631 | 0,244 | 0,473 | 0,170 | 0,455 |
|  | **Inositol** | 8 | -0,0154 | 0,1422 | 8 | -0,2352 | 0,2266 | 8 | -0,0804 | 0,2153 | 8 | -0,3704 | 0,1496 | 24 | -0,2287 | 0,1131 | -0,220 | 0,425 | -0,065 | 0,805 | -0,355 | 0,107 | -0,213 | 0,325 |
|  | **Kynurenic acid** | 8 | -0,0078 | 0,0876 | 8 | 0,2407 | 0,1892 | 7 | -0,0520 | 0,0590 | 8 | 0,0076 | 0,0352 | 23 | 0,0705 | 0,0715 | 0,248 | 0,253 | -0,044 | 0,691 | 0,015 | 0,873 | 0,078 | 0,559 |
|  | **Kynurenine** | 8 | 0,0369 | 0,2113 | 8 | -0,1957 | 0,3272 | 8 | 0,2156 | 0,2513 | 8 | -0,3164 | 0,2247 | 24 | -0,0988 | 0,1570 | -0,233 | 0,560 | 0,179 | 0,595 | -0,353 | 0,271 | -0,136 | 0,653 |
|  | **NAAG** | 8 | 0,0106 | 0,0608 | 8 | -0,1447 | 0,3894 | 8 | -0,2994 | 0,2687 | 8 | 0,1011 | 0,1101 | 24 | -0,1143 | 0,1585 | -0,155 | 0,699 | -0,310 | 0,279 | 0,091 | 0,484 | -0,125 | 0,658 |
|  | **Niacinamide** | 8 | 0,0118 | 0,2065 | 8 | -0,0144 | 0,2448 | 8 | -0,1472 | 0,1872 | 8 | 0,1277 | 0,1306 | 24 | -0,0113 | 0,1091 | -0,026 | 0,936 | -0,159 | 0,578 | 0,116 | 0,642 | -0,023 | 0,918 |
|  | **Phenylbutyric acid** | 8 | 0,0269 | 0,0608 | 7 | -0,1374 | 0,0419 | 7 | -0,0528 | 0,0354 | 6 | -0,1657 | 0,0337 | 20 | -0,1163 | 0,0233 | -0,164 | 0,050 | -0,080 | 0,296 | -0,193 | 0,027 | -0,143 | 0,012 |
|  | **Phosphoric acid** | 8 | 0,0023 | 0,0434 | 8 | -0,1117 | 0,0548 | 8 | -0,0573 | 0,0675 | 8 | -0,1297 | 0,0710 | 24 | -0,0996 | 0,0363 | -0,114 | 0,125 | -0,060 | 0,470 | -0,132 | 0,135 | -0,102 | 0,145 |
|  | **Shikimic acid** | 8 | 0,0223 | 0,2387 | 8 | 0,1004 | 0,1535 | 8 | 0,4500 | 0,1666 | 8 | 0,4146 | 0,1506 | 24 | 0,3217 | 0,0926 | 0,078 | 0,787 | 0,428 | 0,164 | 0,392 | 0,186 | 0,299 | 0,164 |
|  | **Tryptamine** | 8 | -0,0150 | 0,0887 | 8 | 0,0059 | 0,1144 | 8 | 0,2759 | 0,1388 | 8 | 0,2547 | 0,0550 | 24 | 0,1788 | 0,0651 | 0,021 | 0,887 | 0,291 | 0,099 | 0,270 | 0,022 | 0,194 | 0,129 |
|  | **Tryptophan** | 6 | -0,2707 | 0,1604 | 8 | -0,2944 | 0,2604 | 6 | 0,0091 | 0,1508 | 8 | -0,0325 | 0,2617 | 22 | -0,1164 | 0,1374 | -0,024 | 0,945 | 0,280 | 0,233 | 0,238 | 0,490 | 0,154 | 0,584 |
| ***Ratios*** | ***aHGA/aKG*** | *8* | *0,0089* | *0,1318* | *8* | *-0,1665* | *0,2637* | *8* | *-0,7407* | *0,2776* | *8* | *-0,8283* | *0,0839* | *24* | *-0,5785* | *0,1390* | *-0,175* | *0,561* | *-0,750* | *0,029* | *-0,837* | *0,000* | *-0,587* | *0,028* |
|  | ***aKG/Glu*** | *8* | *-0,1204* | *0,3620* | *8* | *-0,5708* | *0,2176* | *8* | *-0,0923* | *0,1550* | *8* | *0,4429* | *0,2526* | *24* | *-0,0734* | *0,1455* | *-0,450* | *0,304* | *0,028* | *0,944* | *0,563* | *0,223* | *0,047* | *0,886* |
|  | ***Asn/Asp*** | *8* | *-0,0032* | *0,0706* | *8* | *-0,3697* | *0,1404* | *8* | *-0,0184* | *0,1871* | *8* | *-0,5970* | *0,1669* | *24* | *-0,3284* | *0,1041* | *-0,367* | *0,035* | *-0,015* | *0,941* | *-0,594* | *0,006* | *-0,325* | *0,092* |
|  | ***Asp/Malate*** | *8* | *0,0680* | *0,2162* | *8* | *-0,0469* | *0,1591* | *8* | *0,2708* | *0,1329* | *8* | *-0,1638* | *0,1698* | *24* | *0,0200* | *0,0936* | *-0,115* | *0,675* | *0,203* | *0,438* | *-0,232* | *0,413* | *-0,048* | *0,815* |
|  | ***HOPro/Pro*** | *8* | *-0,0106* | *0,2711* | *8* | *0,4980* | *0,4055* | *8* | *-0,3975* | *0,3065* | *8* | *0,0546* | *0,2868* | *24* | *0,0517* | *0,2009* | *0,509* | *0,315* | *-0,387* | *0,360* | *0,065* | *0,871* | *0,062* | *0,872* |
|  | ***KIC/Leu*** | *8* | *-0,0070* | *0,1326* | *8* | *0,0581* | *0,1643* | *8* | *-0,2838* | *0,1665* | *8* | *0,0548* | *0,1466* | *24* | *-0,0570* | *0,0941* | *0,065* | *0,762* | *-0,277* | *0,215* | *0,062* | *0,759* | *-0,050* | *0,784* |
|  | ***Kyn/Trp*** | *8* | *-0,0061* | *0,1253* | *8* | *-0,0237* | *0,1514* | *8* | *-0,2427* | *0,1993* | *8* | *-0,3332* | *0,1348* | *24* | *-0,1999* | *0,0945* | *-0,018* | *0,930* | *-0,237* | *0,332* | *-0,327* | *0,097* | *-0,194* | *0,289* |
|  | ***Met/Cys*** | *8* | *-0,1248* | *0,3980* | *8* | *0,1509* | *0,3786* | *8* | *-0,4476* | *0,2678* | *8* | *0,4808* | *0,2248* | *24* | *0,0614* | *0,1827* | *0,276* | *0,623* | *-0,323* | *0,512* | *0,606* | *0,206* | *0,186* | *0,636* |
|  | ***Orn/Arg*** | *8* | *-0,0516* | *0,1618* | *8* | *-0,1613* | *0,2129* | *8* | *-0,0799* | *0,1372* | *8* | *0,3209* | *0,2650* | *24* | *0,0265* | *0,1248* | *-0,110* | *0,688* | *-0,028* | *0,896* | *0,372* | *0,250* | *0,078* | *0,743* |
|  | ***Ser/Gly*** | *8* | *-0,0270* | *0,2403* | *8* | *-0,0009* | *0,1867* | *8* | *0,2356* | *0,1423* | *8* | *0,2009* | *0,1675* | *24* | *0,1452* | *0,0944* | *0,026* | *0,933* | *0,263* | *0,363* | *0,228* | *0,449* | *0,172* | *0,425* |
|  | ***Tyr/Phe*** | *8* | *0,0147* | *0,1315* | *8* | *-0,1766* | *0,2088* | *8* | *0,3255* | *0,1003* | *8* | *0,0075* | *0,0772* | *24* | *0,0521* | *0,0890* | *-0,191* | *0,451* | *0,311* | *0,081* | *-0,007* | *0,963* | *0,037* | *0,830* |
|  | ***GABA/Glu*** | *8* | *-0,0714* | *0,1711* | *8* | *-0,0509* | *0,5039* | *8* | *-0,5741* | *0,3692* | *8* | *-0,7617* | *0,2255* | *24* | *-0,4623* | *0,2206* | *0,021* | *0,970* | *-0,503* | *0,237* | *-0,690* | *0,029* | *-0,391* | *0,334* |
|  | ***Glu/Gln*** | *8* | *0,0719* | *0,2172* | *8* | *0,5435* | *0,1540* | *8* | *0,1224* | *0,2302* | *8* | *0,3500* | *0,3150* | *24* | *0,3386* | *0,1383* | *0,472* | *0,098* | *0,051* | *0,875* | *0,278* | *0,479* | *0,267* | *0,333* |
|  | ***Glu/Oxoproline*** | *8* | *0,0966* | *0,2470* | *8* | *0,5874* | *0,2121* | *8* | *0,5869* | *0,2590* | *8* | *0,7892* | *0,2518* | *24* | *0,6545* | *0,1349* | *0,491* | *0,154* | *0,490* | *0,192* | *0,693* | *0,070* | *0,558* | *0,050* |
|  | ***Cys/Ser*** | *8* | *0,1504* | *0,4247* | *8* | *0,0663* | *0,3795* | *8* | *0,8653* | *0,3160* | *8* | *0,1659* | *0,2752* | *24* | *0,3658* | *0,1947* | *-0,084* | *0,885* | *0,715* | *0,198* | *0,016* | *0,976* | *0,215* | *0,608* |
|  | ***DMGly/Betaine*** | *8* | *0,0309* | *0,1097* | *8* | *-0,2542* | *0,1267* | *8* | *0,0892* | *0,1722* | *8* | *-0,1411* | *0,0836* | *24* | *-0,1020* | *0,0790* | *-0,285* | *0,111* | *0,058* | *0,779* | *-0,172* | *0,233* | *-0,133* | *0,386* |
|  | ***Guanine/Guanosine*** | *7* | *0,2440* | *0,6157* | *8* | *0,1633* | *0,4322* | *7* | *-0,6790* | *0,5063* | *8* | *-1,1212* | *0,4196* | *23* | *-0,5398* | *0,2736* | *-0,081* | *0,914* | *-0,923* | *0,269* | *-1,365* | *0,084* | *-0,784* | *0,201* |
|  | ***HXanthine/Inosine*** | *8* | *0,2643* | *0,4490* | *8* | *1,8781* | *0,4020* | *8* | *2,1806* | *0,2930* | *8* | *2,5171* | *0,3422* | *24* | *2,1919* | *0,1999* | *1,614* | *0,018* | *1,916* | *0,003* | *2,253* | *0,001* | *1,928* | *0,000* |
|  | ***Inosine/Adenosine*** | *8* | *-0,0150* | *0,2946* | *8* | *-0,8408* | *0,1450* | *8* | *-0,8942* | *0,2384* | *8* | *-0,8898* | *0,2482* | *24* | *-0,8749* | *0,1190* | *-0,826* | *0,025* | *-0,879* | *0,036* | *-0,875* | *0,039* | *-0,860* | *0,003* |
|  | ***Urate/Xanthine*** | *8* | *-0,0028* | *0,1309* | *8* | *0,0492* | *0,3505* | *8* | *-0,8811* | *0,2888* | *8* | *-0,7799* | *0,4473* | *24* | *-0,5372* | *0,2208* | *0,052* | *0,891* | *-0,878* | *0,015* | *-0,777* | *0,118* | *-0,534* | *0,185* |
|  | ***Urea/Arginine*** | *8* | *-0,0892* | *0,3140* | *8* | *0,1042* | *0,2496* | *8* | *-0,1758* | *0,2077* | *8* | *0,5424* | *0,2831* | *24* | *0,1569* | *0,1504* | *0,193* | *0,637* | *-0,087* | *0,821* | *0,632* | *0,157* | *0,246* | *0,443* |
|  | ***Xanthine/Guanine*** | *8* | *0,0011* | *0,1305* | *8* | *-0,0575* | *0,3529* | *8* | *0,8835* | *0,2915* | *8* | *0,7641* | *0,4475* | *24* | *0,5300* | *0,2218* | *-0,059* | *0,879* | *0,882* | *0,015* | *0,763* | *0,124* | *0,529* | *0,191* |
|  | ***Xanthine/HXanthine*** | *8* | *-0,2992* | *0,4299* | *8* | *-1,3834* | *0,3062* | *8* | *-1,2766* | *0,3092* | *8* | *-1,5739* | *0,3002* | *24* | *-1,4113* | *0,1703* | *-1,084* | *0,059* | *-0,977* | *0,086* | *-1,275* | *0,029* | *-1,112* | *0,007* |
|  | ***Hypox/Uric*** | *8* | *0,0624* | *0,3683* | *8* | *1,3412* | *0,3045* | *8* | *2,0536* | *0,2694* | *8* | *2,2928* | *0,3414* | *24* | *1,8958* | *0,1889* | *1,279* | *0,018* | *1,991* | *0,001* | *2,230* | *0,001* | *1,833* | *0,000* |
|  | ***DCA/GDCA*** | *8* | *0,0001* | *0,2722* | *8* | *-0,5808* | *0,3849* | *8* | *-0,3106* | *0,2258* | *8* | *0,3900* | *0,1649* | *24* | *-0,1671* | *0,1739* | *-0,581* | *0,238* | *-0,311* | *0,395* | *0,390* | *0,241* | *-0,167* | *0,627* |
|  | ***DCA/TDCA*** | *8* | *-0,0949* | *0,2278* | *8* | *-0,6765* | *0,1761* | *8* | *-0,6547* | *0,2645* | *8* | *-1,0073* | *0,2549* | *24* | *-0,7795* | *0,1340* | *-0,582* | *0,063* | *-0,560* | *0,131* | *-0,912* | *0,018* | *-0,685* | *0,016* |
|  | ***CA/GCA*** | *8* | *0,0152* | *0,2321* | *8* | *-0,9722* | *0,4095* | *8* | *0,3200* | *0,4916* | *8* | *0,5591* | *0,2347* | *24* | *-0,0310* | *0,2584* | *-0,987* | *0,055* | *0,305* | *0,584* | *0,544* | *0,122* | *-0,046* | *0,922* |
|  | ***CA/TCA*** | *8* | *0,0857* | *0,2103* | *8* | *-0,5087* | *0,2801* | *8* | *-0,9755* | *0,3039* | *8* | *-1,3406* | *0,3309* | *24* | *-0,9416* | *0,1830* | *-0,594* | *0,112* | *-1,061* | *0,012* | *-1,426* | *0,003* | *-1,027* | *0,005* |
|  | ***Xylose/Xylitol*** | *8* | *-0,0077* | *0,4743* | *8* | *-0,0549* | *0,4694* | *8* | *-0,5054* | *0,2909* | *8* | *-0,1831* | *0,4124* | *24* | *-0,2478* | *0,2231* | *-0,047* | *0,945* | *-0,498* | *0,386* | *-0,175* | *0,784* | *-0,240* | *0,615* |
|  | ***Glc6P/Glc*** | *8* | *-0,0614* | *0,2310* | *8* | *0,4155* | *0,2739* | *8* | *1,3263* | *0,3638* | *8* | *1,3384* | *0,3714* | *24* | *1,0268* | *0,2077* | *0,477* | *0,204* | *1,388* | *0,006* | *1,400* | *0,006* | *1,088* | *0,008* |
|  | ***AcCarn/Carn*** | *8* | *-0,0063* | *0,1981* | *8* | *-0,3216* | *0,2024* | *8* | *-0,1998* | *0,1409* | *8* | *-0,1820* | *0,1508* | *24* | *-0,2345* | *0,0930* | *-0,315* | *0,284* | *-0,194* | *0,439* | *-0,176* | *0,492* | *-0,228* | *0,255* |
|  | ***Stearate/Linolenate*** | *8* | *-0,0795* | *0,3654* | *8* | *0,3303* | *0,1871* | *8* | *0,0738* | *0,3522* | *8* | *0,4069* | *0,2286* | *24* | *0,2703* | *0,1494* | *0,410* | *0,335* | *0,153* | *0,767* | *0,486* | *0,278* | *0,350* | *0,300* |
|  | ***Glyc3P/Glycerol*** | *8* | *-0,0128* | *0,2998* | *8* | *-0,2815* | *0,3776* | *8* | *-0,5682* | *0,3528* | *8* | *-0,4019* | *0,3357* | *24* | *-0,4172* | *0,1978* | *-0,269* | *0,586* | *-0,555* | *0,250* | *-0,389* | *0,402* | *-0,404* | *0,302* |
|  | ***Glyc3P/GPCho*** | *8* | *-0,0487* | *0,1777* | *8* | *0,9897* | *0,1754* | *8* | *0,0223* | *0,2465* | *8* | *-0,1357* | *0,2297* | *24* | *0,2921* | *0,1594* | *1,038* | *0,001* | *0,071* | *0,819* | *-0,087* | *0,769* | *0,341* | *0,259* |
|  | ***OPEt/S1P*** | *8* | *-0,0263* | *0,1091* | *8* | *-0,1640* | *0,0975* | *8* | *-0,6486* | *0,1351* | *8* | *-0,6044* | *0,2102* | *24* | *-0,4723* | *0,0969* | *-0,138* | *0,363* | *-0,622* | *0,003* | *-0,578* | *0,029* | *-0,446* | *0,019* |
|  | ***DeOGuanosine/Guanine*** | *8* | *0,0155* | *0,2675* | *8* | *0,2620* | *0,3823* | *8* | *0,8139* | *0,4073* | *8* | *0,6510* | *0,3383* | *24* | *0,5756* | *0,2135* | *0,247* | *0,606* | *0,798* | *0,124* | *0,635* | *0,163* | *0,560* | *0,174* |
|  | ***Uridine/UMP*** | *8* | *-0,1270* | *0,3483* | *8* | *-0,7426* | *0,4476* | *8* | *-0,5079* | *0,3703* | *8* | *-0,6017* | *0,6991* | *24* | *-0,6174* | *0,2902* | *-0,616* | *0,296* | *-0,381* | *0,466* | *-0,475* | *0,553* | *-0,490* | *0,375* |
|  | ***Fum/Malate*** | *8* | *-0,0029* | *0,0798* | *8* | *-0,2940* | *0,1146* | *8* | *-0,0691* | *0,1640* | *8* | *-0,4563* | *0,1010* | *24* | *-0,2731* | *0,0787* | *-0,291* | *0,056* | *-0,066* | *0,722* | *-0,453* | *0,003* | *-0,270* | *0,072* |
|  | ***Cit/Arg*** | *8* | *-0,0592* | *0,1625* | *8* | *-0,0016* | *0,1535* | *8* | *-0,0459* | *0,1951* | *8* | *0,1694* | *0,1335* | *24* | *0,0406* | *0,0919* | *0,058* | *0,800* | *0,013* | *0,959* | *0,229* | *0,295* | *0,100* | *0,593* |
|  | ***Cit/Orn*** | *7* | *0,0073* | *0,0668* | *8* | *0,1597* | *0,0963* | *8* | *-0,0069* | *0,1206* | *8* | *-0,1466* | *0,1568* | *24* | *0,0021* | *0,0748* | *0,152* | *0,229* | *-0,014* | *0,923* | *-0,154* | *0,407* | *-0,005* | *0,971* |
|  | ***Citrate/Oaa*** | *8* | *0,0278* | *0,2202* | *8* | *-0,5390* | *0,3131* | *8* | *-0,9267* | *0,5486* | *8* | *-2,1433* | *0,1414* | *24* | *-1,2030* | *0,2506* | *-0,567* | *0,161* | *-0,954* | *0,129* | *-2,171* | *0,000* | *-1,231* | *0,011* |
|  | ***Oaa/Asp*** | *8* | *-0,0855* | *0,4037* | *8* | *0,1427* | *0,2960* | *8* | *0,4611* | *0,3332* | *8* | *1,4956* | *0,0914* | *24* | *0,6998* | *0,1884* | *0,228* | *0,655* | *0,547* | *0,314* | *1,581* | *0,002* | *0,785* | *0,059* |
|  | ***Oaa/Malate*** | *8* | *-0,0175* | *0,2481* | *8* | *0,0957* | *0,2846* | *8* | *0,7319* | *0,4071* | *8* | *1,3318* | *0,1063* | *24* | *0,7198* | *0,1930* | *0,113* | *0,769* | *0,749* | *0,138* | *1,349* | *0,000* | *0,737* | *0,053* |
|  | ***PEP/Oaa*** | *8* | *0,0221* | *0,2030* | *8* | *-0,0379* | *0,2917* | *8* | *-0,4307* | *0,4355* | *8* | *-1,4077* | *0,2213* | *24* | *-0,6254* | *0,2174* | *-0,060* | *0,868* | *-0,453* | *0,362* | *-1,430* | *0,000* | *-0,648* | *0,113* |
|  | ***Pyr/Ala*** | *8* | *-0,0728* | *0,4711* | *8* | *0,0774* | *0,3291* | *8* | *0,2645* | *0,3311* | *8* | *1,2566* | *0,1129* | *24* | *0,5328* | *0,1872* | *0,150* | *0,798* | *0,337* | *0,567* | *1,329* | *0,016* | *0,606* | *0,160* |
|  | ***Pyr/Cys*** | *8* | *-0,2078* | *0,6025* | *8* | *0,4218* | *0,4644* | *8* | *-0,2014* | *0,4995* | *8* | *1,9869* | *0,3996* | *24* | *0,7357* | *0,3166* | *0,630* | *0,422* | *0,006* | *0,994* | *2,195* | *0,009* | *0,944* | *0,156* |
|  | ***Pyr/Lac*** | *8* | *-0,0242* | *0,1829* | *8* | *0,2501* | *0,2192* | *8* | *0,8848* | *0,3855* | *8* | *1,5983* | *0,0684* | *24* | *0,9111* | *0,1833* | *0,274* | *0,353* | *0,909* | *0,051* | *1,623* | *0,000* | *0,935* | *0,009* |
|  | ***Pyr/Malate*** | *8* | *-0,0175* | *0,2481* | *8* | *0,0957* | *0,2846* | *8* | *0,6039* | *0,4744* | *8* | *1,3318* | *0,1063* | *24* | *0,6772* | *0,2083* | *0,113* | *0,769* | *0,621* | *0,265* | *1,349* | *0,000* | *0,695* | *0,085* |
|  | ***Pyr/PEP*** | *8* | *-0,0221* | *0,2030* | *8* | *0,0379* | *0,2917* | *8* | *0,4307* | *0,4355* | *8* | *1,4077* | *0,2213* | *24* | *0,6254* | *0,2174* | *0,060* | *0,868* | *0,453* | *0,362* | *1,430* | *0,000* | *0,648* | *0,113* |
|  | ***Pyr/Ser*** | *8* | *-0,0574* | *0,3639* | *8* | *0,4880* | *0,3721* | *8* | *0,6638* | *0,3797* | *8* | *2,1528* | *0,2802* | *24* | *1,1015* | *0,2468* | *0,545* | *0,312* | *0,721* | *0,192* | *2,210* | *0,000* | *1,159* | *0,021* |
|  | ***Val/KIV*** | *8* | *0,0065* | *0,1624* | *8* | *-0,0707* | *0,1910* | *8* | *-0,0128* | *0,2462* | *8* | *-0,4484* | *0,1474* | *24* | *-0,1773* | *0,1170* | *-0,077* | *0,763* | *-0,019* | *0,949* | *-0,455* | *0,057* | *-0,184* | *0,418* |
