# Supplementary material for: Metabolic alterations in plasma from patients with familial and idiopathic Parkinson’s disease
Source: Aging (Albany NY). 2020 Sep 9;12(17):16690–708. doi: 10.18632/aging.103992 (PMC7521510; doi:10.18632/aging.103992)
Supplement: Supplementary Table 2 [file aging-12-103992-s001..docx]

**Supplementary Table 2. Metabolite changes in plasma from 6-OHDA mouse model.**

| **Mice plasma samples** | | **Area values** | | | | | | **Fold Change (Log2)** | |
| --- | --- | --- | --- | --- | --- | --- | --- | --- | --- |
|  |  | **Healthy (Untreated)** | | | **PD (6-OHDA)** | | | **6-OHDA vs Unt** | |
| **Group** | **Metabolite** | **N** | **Average** | **SEM** | **N** | **Average** | **SEM** | **Fold Change** | **TTEST (pvalue)** |
| **Amino acids** | **2-aminoadipic acid** | 10 | 4,44089E-17 | 0,2316 | 8 | 0,4051 | 0,2270 | 0,4051 | 0,3370 |
|  | **3-methylhistidine** | 10 | 0,000 | 0,0939 | 8 | -0,1882 | 0,1170 | -0,1882 | 0,2120 |
|  | **4-hydroxyproline** | 10 | 0 | 0,0880 | 8 | -0,2897 | 0,0650 | -0,2897 | 0,1070 |
|  | **5-aminovaleric acid** | 10 | 1,07553E-17 | 0,0984 | 8 | -0,1553 | 0,1362 | -0,1553 | 0,3197 |
|  | **5-oxoproline** | 10 | 0 | 0,1009 | 8 | 0,1532 | 0,0565 | 0,1532 | 0,2810 |
|  | **Alanine** | 10 | 0,018877778 | 0,0573 | 8 | -0,1672 | 0,0996 | -0,1861 | 0,0480 |
|  | **Arginine** | 10 | 0 | 0,1016 | 8 | 0,1037 | 0,0828 | 0,1037 | 0,4813 |
|  | **Asparagine** | 10 | 0 | 0,1066 | 8 | 0,3462 | 0,1252 | 0,3462 | 0,0442 |
|  | **Aspartic acid** | 10 | 0 | 0,0818 | 8 | 0,4532 | 0,1704 | 0,4532 | 0,0019 |
|  | **Beta-alanine** | 10 | 4,21885E-16 | 0,1447 | 8 | 1,8646 | 0,1472 | 1,8646 | 0,0000 |
|  | **Betaine;Valine** | 10 | 0 | 0,0943 | 8 | -0,1398 | 0,0671 | -0,1398 | 0,2426 |
|  | **Citrulline** | 10 | -4,44089E-17 | 0,0637 | 8 | 0,8053 | 0,1272 | 0,8053 | 0,0000 |
|  | **Creatine** | 10 | 0 | 0,1673 | 8 | -0,4651 | 0,1330 | -0,4651 | 0,1060 |
|  | **Creatinine** | 10 | -8,88178E-17 | 0,0793 | 8 | 0,3566 | 0,1163 | 0,3566 | 0,0667 |
|  | **Cystine** | 10 | 1,11022E-16 | 0,3176 | 8 | -0,7783 | 0,2754 | -0,7783 | 0,0631 |
|  | **Dimethylarginine** | 10 | 0 | 0,0929 | 8 | -0,0487 | 0,1123 | -0,0487 | 0,7003 |
|  | **Dimethylglycine** | 10 | 0 | 0,0436 | 8 | -0,2673 | 0,0795 | -0,2673 | 0,0103 |
|  | **GABA** | 10 | 6,93889E-18 | 0,0872 | 8 | -0,1173 | 0,0620 | -0,1173 | 0,5469 |
|  | **Glutamic acid** | 10 | -2,22045E-17 | 0,0653 | 8 | 0,4787 | 0,1461 | 0,4787 | 0,0006 |
|  | **Glutamine** | 10 | 2,77556E-17 | 0,1310 | 8 | 0,3723 | 0,1563 | 0,3723 | 0,0660 |
|  | **Glycine** | 10 | 0 | 0,0610 | 8 | 0,0885 | 0,0925 | 0,0885 | 0,5280 |
|  | **Histidine** | 10 | 0 | 0,0846 | 8 | 0,3882 | 0,1460 | 0,3882 | 0,0115 |
|  | **Homoserine** | 10 | -4,16334E-17 | 0,1121 | 8 | -0,0043 | 0,1289 | -0,0043 | 0,9822 |
|  | **Isoleucine** | 10 | 0 | 0,1521 | 8 | -0,0092 | 0,1923 | -0,0092 | 0,9656 |
|  | **Leucine** | 10 | 0 | 0,1313 | 8 | 0,1143 | 0,1404 | 0,1143 | 0,4907 |
|  | **Lysine** | 10 | 0 | 0,0705 | 8 | 0,3492 | 0,1165 | 0,3492 | 0,0087 |
|  | **Methionine** | 10 | 0 | 0,0909 | 8 | -0,2363 | 0,1095 | -0,2363 | 0,1325 |
|  | **N-acetylaspartic acid** | 10 | 0 | 0,4290 | 8 | 0,3111 | 0,7059 | 0,3111 | 0,6513 |
|  | **N-acetylglutamic acid** | 10 | 8,32667E-17 | 0,1169 | 8 | 0,1583 | 0,2299 | 0,1583 | 0,4291 |
|  | **N-acetylglutamine** | 10 | -3,88578E-17 | 0,0905 | 8 | -0,3105 | 0,1687 | -0,3105 | 0,0341 |
|  | **N1-acetylspermidine;N8-acetylspermidine** | 10 | 5,89806E-18 | 0,0638 | 8 | -0,2592 | 0,0653 | -0,2592 | 0,0543 |
|  | **N6-acetyllysine;N2-acetyllysine** | 10 | 0 | 0,0811 | 8 | 0,0760 | 0,0788 | 0,0760 | 0,4978 |
|  | **Ornithine** | 10 | -2,77556E-18 | 0,0530 | 8 | 0,1537 | 0,0860 | 0,1537 | 0,1546 |
|  | **Pantothenic acid** | 10 | 4,44089E-17 | 0,0775 | 8 | 0,3400 | 0,0764 | 0,3400 | 0,0104 |
|  | **Phenylalanine** | 10 | -8,32667E-18 | 0,0614 | 8 | 0,2082 | 0,0816 | 0,2082 | 0,0416 |
|  | **Phosphocreatine** | 10 | -3,60822E-17 | 0,1571 | 8 | 0,0443 | 0,1711 | 0,0443 | 0,8946 |
|  | **Proline** | 10 | -1,33227E-16 | 0,0891 | 8 | -0,8813 | 0,1366 | -0,8813 | 0,0000 |
|  | **Proline betaine** | 10 | 7,77156E-17 | 0,1334 | 8 | 0,1666 | 0,1113 | 0,1666 | 0,3089 |
|  | **Serine** | 10 | 4,996E-17 | 0,1147 | 8 | 0,2192 | 0,2030 | 0,2192 | 0,3058 |
|  | **Threonine** | 10 | -3,88578E-17 | 0,0724 | 8 | 0,0334 | 0,0812 | 0,0334 | 0,8209 |
|  | **Trimethyl-lysine** | 10 | 0 | 0,1727 | 8 | 0,0808 | 0,2238 | 0,0808 | 0,7041 |
|  | **Tyrosine** | 10 | -5,55112E-17 | 0,0935 | 8 | 0,2140 | 0,1045 | 0,2140 | 0,1317 |
|  | **Valine** | 10 | -6,93889E-18 | 0,0713 | 8 | -0,0265 | 0,1054 | -0,0265 | 0,8035 |
| **Bases** | **Cytosine** | 10 | 0 | 0,0789 | 8 | -0,2241 | 0,1139 | -0,2241 | 0,0989 |
|  | **Guanine** | 10 | -5,55112E-18 | 0,0425 | 8 | 0,2624 | 0,4066 | 0,2624 | 0,0111 |
|  | **Hypoxanthine** | 10 | 8,88178E-17 | 0,1013 | 8 | 2,8808 | 0,7673 | 2,8808 | 0,0032 |
|  | **Thymine** | 10 | 0 | 0,0705 | 8 | 0,3550 | 0,0712 | 0,3550 | 0,0066 |
|  | **Uracil** | 10 | 5,55112E-17 | 0,1264 | 8 | 0,6911 | 0,1327 | 0,6911 | 0,0019 |
|  | **Uric acid** | 10 | 0 | 0,0310 | 8 | 0,3685 | 0,4074 | 0,3685 | 0,0000 |
|  | **Xanthine** | 10 | 1,753725 | 1,2478 | 8 | 6,8240 | 1,5698 | 5,0703 | 0,0209 |
| **Bilic acids** | **CA** | 10 | 1,77636E-16 | 0,2372 | 8 | 1,1602 | 0,2933 | 1,1602 | 0,0253 |
|  | **DCA** | 10 | 0 | 0,2707 | 8 | 0,6758 | 0,3191 | 0,6758 | 0,1470 |
|  | **G-CA** | 10 | 0 | 0,1147 | 8 | 0,0984 | 0,0715 | 0,0984 | 0,5091 |
|  | **LCA** | 10 | 0 | 0,3034 | 7 | 1,2591 | 0,4294 | 1,2591 | 0,0287 |
|  | **T-CA** | 10 | -1,33227E-16 | 0,3535 | 8 | 0,3136 | 0,1149 | 0,3136 | 0,5899 |
|  | **T-CDCA** | 10 | 0 | 0,2200 | 8 | 0,7768 | 0,1342 | 0,7768 | 0,0859 |
|  | **T-DCA** | 10 | -1,33227E-16 | 0,2283 | 8 | 0,3956 | 0,0908 | 0,3956 | 0,2612 |
|  | **T-UDCA** | 10 | -1,55431E-16 | 0,1854 | 8 | 0,6008 | 0,2130 | 0,6008 | 0,1243 |
|  | **Cholesterol** | 10 | 0 | 0,0523 | 8 | -0,0456 | 0,0785 | -0,0456 | 0,6457 |
| **Carbohydrates** | **Arabinose** | 10 | 2,22045E-17 | 0,0984 | 8 | 0,1916 | 0,0707 | 0,1916 | 0,2151 |
|  | **Arabitol** | 10 | -2,498E-17 | 0,1172 | 8 | -0,1255 | 0,1343 | -0,1255 | 0,5147 |
|  | **Erythritol** | 10 | 0 | 0,0718 | 8 | 0,0796 | 0,1890 | 0,0796 | 0,5074 |
|  | **Fructose** | 10 | 8,32667E-18 | 0,0878 | 8 | 0,3701 | 0,0831 | 0,3701 | 0,1629 |
|  | **Galactitol;Sorbitol;Mannitol** | 10 | 0 | 0,0566 | 8 | -0,0797 | 0,0791 | -0,0797 | 0,2957 |
|  | **Galactose;Glucose;Mannose** | 10 | 0 | 0,0488 | 8 | -0,1310 | 0,0515 | -0,1310 | 0,0487 |
|  | **Glucose-6-phosphate** | 10 | 0 | 0,1831 | 8 | 0,5167 | 0,2697 | 0,5167 | 0,0448 |
|  | **Glyceric acid** | 10 | 0 | 0,0661 | 8 | 0,1434 | 0,1036 | 0,1434 | 0,1983 |
|  | **Glycerol** | 10 | 0 | 0,0949 | 8 | -0,1417 | 0,0677 | -0,1417 | 0,4437 |
|  | **Lactose** | 10 | -2,22045E-17 | 0,1587 | 8 | 0,2440 | 0,1641 | 0,2440 | 0,3431 |
|  | **Maltose;Trehalose** | 10 | 0 | 0,1972 | 8 | -0,3021 | 0,3637 | -0,3021 | 0,2122 |
|  | **Raffinose** | 10 | 0 | 0,2160 | 8 | 0,8324 | 0,2812 | 0,8324 | 0,1916 |
|  | **Ribitol** | 10 | -3,60822E-17 | 0,0874 | 8 | 0,3025 | 0,1270 | 0,3025 | 0,0726 |
|  | **Ribose-5-phosphate** | 10 | 0 | 0,2626 | 8 | 0,6914 | 0,3470 | 0,6914 | 0,2323 |
|  | **Ribose;Ribulose** | 10 | -8,88178E-17 | 0,1652 | 8 | 0,3581 | 0,1180 | 0,3581 | 0,1545 |
|  | **Sucrose** | 10 | 0 | 0,6601 | 8 | 0,4640 | 0,1643 | 0,4640 | 0,6304 |
|  | **Threonic acid** | 10 | 1,22125E-16 | 0,0765 | 8 | 0,2416 | 0,0840 | 0,2416 | 0,0617 |
|  | **Xylitol** | 10 | -4,996E-17 | 0,0702 | 8 | 0,1908 | 0,1150 | 0,1908 | 0,1398 |
|  | **Xylose** | 10 | 0 | 0,2147 | 8 | -0,8208 | 0,1499 | -0,8208 | 0,0074 |
| **Fatty Acyls** | **3-hydroxyisovaleric acid** | 10 | 0 | 0,0436 | 8 | -0,0470 | 0,0663 | -0,0470 | 0,6140 |
|  | **Acetylcarnitine** | 10 | 0 | 0,1493 | 8 | 0,2980 | 0,0429 | 0,2980 | 0,1554 |
|  | **Arachidic acid** | 10 | 3,33067E-17 | 0,1156 | 8 | 0,1079 | 0,0885 | 0,1079 | 0,4814 |
|  | **Arachidonic acid** | 10 | 3,88578E-17 | 0,1089 | 8 | 0,6710 | 0,0858 | 0,6710 | 0,0007 |
|  | **Behenic acid** | 10 | 1,38778E-17 | 0,1165 | 8 | 0,1376 | 0,0483 | 0,1376 | 0,3682 |
|  | **Capric acid** | 10 | 2,22045E-17 | 0,0617 | 8 | 0,0854 | 0,0641 | 0,0854 | 0,4005 |
|  | **Caproic acid** | 10 | 4,44089E-17 | 0,1007 | 8 | -0,1568 | 0,1206 | -0,1568 | 0,2956 |
|  | **Carnitine C10:0** | 10 | 0 | 0,1368 | 8 | 0,8812 | 0,2887 | 0,8812 | 0,0007 |
|  | **Carnitine C10:1** | 10 | 1,88738E-16 | 0,1852 | 8 | 1,0040 | 0,0894 | 1,0040 | 0,0037 |
|  | **Carnitine C12:0** | 10 | 0 | 0,1326 | 8 | 0,8631 | 0,0865 | 0,8631 | 0,0002 |
|  | **Carnitine C12:1** | 3 | 0 | 0,2324 | 6 | 1,1637 | 0,1901 | 1,1637 | 0,0003 |
|  | **Carnitine C14:0** | 10 | -0,028388889 | 0,1191 | 8 | 0,7607 | 0,0825 | 0,7891 | 0,0002 |
|  | **Carnitine C14:0(OH)** | 10 | 0 | 0,1453 | 8 | 1,0905 | 0,1864 | 1,0905 | 0,0000 |
|  | **Carnitine C14:1** | 10 | 0 | 0,1427 | 8 | 1,1798 | 0,1123 | 1,1798 | 0,0002 |
|  | **Carnitine C14:2** | 10 | 0 | 0,2064 | 8 | 1,5116 | 0,0747 | 1,5116 | 0,0003 |
|  | **Carnitine C16:0** | 10 | 0 | 0,1344 | 8 | 0,5962 | 0,0966 | 0,5962 | 0,0048 |
|  | **Carnitine C16:1** | 10 | 0 | 0,1651 | 8 | 1,1294 | 0,1274 | 1,1294 | 0,0006 |
|  | **Carnitine C16:2** | 10 | 1,22125E-16 | 0,1835 | 8 | 1,5311 | 0,0951 | 1,5311 | 0,0001 |
|  | **Carnitine C18:0** | 10 | 0 | 0,1544 | 7 | 0,6536 | 0,1053 | 0,6536 | 0,0063 |
|  | **Carnitine C18:1** | 10 | 0 | 0,1811 | 8 | 1,1213 | 0,1202 | 1,1213 | 0,0012 |
|  | **Carnitine C18:2** | 10 | 0 | 0,1908 | 8 | 1,0965 | 0,1089 | 1,0965 | 0,0011 |
|  | **Carnitine C3:0** | 10 | -1,11022E-17 | 0,1995 | 8 | 0,1730 | 0,2000 | 0,1730 | 0,5155 |
|  | **Carnitine C4:0** | 10 | -3,33067E-17 | 0,1508 | 8 | 0,3996 | 0,3636 | 0,3996 | 0,0622 |
|  | **Carnitine C4:0(OH)** | 10 | 0 | 0,1434 | 8 | 0,1086 | 0,0983 | 0,1086 | 0,5935 |
|  | **Carnitine C5:0** | 10 | 0 | 0,1246 | 8 | 0,3157 | 0,0677 | 0,3157 | 0,0987 |
|  | **Carnitine C6:0** | 10 | 4,44089E-17 | 0,2263 | 8 | 0,9207 | 0,6461 | 0,9207 | 0,0131 |
|  | **Carnitine C8:0** | 10 | -1,11022E-16 | 0,1576 | 8 | 0,8849 | 0,6004 | 0,8849 | 0,0013 |
|  | **Docosadienoic acid** | 10 | 0 | 0,2256 | 8 | 0,6120 | 0,1313 | 0,6120 | 0,0428 |
|  | **Docosahexaenoic acid** | 10 | 0 | 0,1536 | 8 | 0,7823 | 0,1448 | 0,7823 | 0,0063 |
|  | **Docosanedioic acid** | 10 | 0 | 0,0737 | 8 | -0,0242 | 0,0987 | -0,0242 | 0,8332 |
|  | **Docosapentaenoic acid** | 10 | 0 | 0,1626 | 8 | 0,5422 | 0,1532 | 0,5422 | 0,0502 |
|  | **Docosatetraenoic acid** | 10 | 0 | 0,1598 | 8 | 0,9972 | 0,1462 | 0,9972 | 0,0005 |
|  | **Docosatrienoic acid** | 10 | 0 | 0,1907 | 8 | 1,0830 | 0,1836 | 1,0830 | 0,0004 |
|  | **Docosenoic acid** | 10 | 0 | 0,2002 | 8 | 0,2503 | 0,1116 | 0,2503 | 0,3757 |
|  | **Dodecanedioic acid** | 8 | 0 | 0,1050 | 8 | 0,1361 | 0,1170 | 0,1361 | 0,3689 |
|  | **Dodecenedioic acid** | 10 | 0 | 0,1039 | 8 | -0,0663 | 0,0735 | -0,0663 | 0,7049 |
|  | **Dodecenoic acid** | 10 | 0 | 0,2056 | 8 | 0,9933 | 0,1351 | 0,9933 | 0,0017 |
|  | **Eicosadienoic acid** | 10 | 0 | 0,2020 | 8 | 0,8476 | 0,1449 | 0,8476 | 0,0046 |
|  | **Eicosapentaenoic acid** | 10 | 0 | 0,0928 | 8 | 0,0380 | 0,1209 | 0,0380 | 0,8192 |
|  | **Eicosatrienoic acid** | 10 | 0 | 0,1368 | 8 | 0,8812 | 0,1560 | 0,8812 | 0,0008 |
|  | **Eicosenoic acid** | 10 | 0 | 0,1965 | 8 | 0,6159 | 0,1210 | 0,6159 | 0,0214 |
|  | **Glutarylcarnitine** | 10 | 6,10623E-17 | 0,1403 | 8 | 0,2440 | 0,2412 | 0,2440 | 0,2822 |
|  | **Heptadecatrienoic acid** | 10 | 0 | 0,1778 | 8 | 0,2014 | 0,1243 | 0,2014 | 0,3557 |
|  | **Heptadecenoic acid** | 10 | 0 | 0,2375 | 8 | 0,5674 | 0,1694 | 0,5674 | 0,0785 |
|  | **Hexadecadienoic acid** | 10 | 0 | 0,2138 | 8 | 0,7480 | 0,1199 | 0,7480 | 0,0129 |
|  | **Hexadecanedioic acid** | 10 | 3,33067E-17 | 0,0699 | 8 | 0,0315 | 0,0593 | 0,0315 | 0,7627 |
|  | **Lauric acid** | 10 | 0 | 0,1058 | 8 | -0,0170 | 0,1553 | -0,0170 | 0,9168 |
|  | **Linoleic acid** | 10 | 0 | 0,1137 | 8 | 0,4420 | 0,0745 | 0,4420 | 0,0194 |
|  | **Linolenic acid** | 10 | 0 | 0,2789 | 8 | 0,5289 | 0,2136 | 0,5289 | 0,1279 |
|  | **Malonylcarnitine** | 10 | 0 | 0,1106 | 8 | -0,1860 | 0,1298 | -0,1860 | 0,3333 |
|  | **Margaric acid** | 10 | 2,91434E-17 | 0,0538 | 8 | 0,1803 | 0,0274 | 0,1803 | 0,0741 |
|  | **Myristic acid** | 10 | 0 | 0,1681 | 8 | 0,3162 | 0,1379 | 0,3162 | 0,1506 |
|  | **Myristoleic acid** | 10 | 0 | 0,1832 | 8 | 0,6301 | 0,1533 | 0,6301 | 0,0220 |
|  | **Nonadecanoic acid** | 10 | 0 | 0,1612 | 8 | 0,6947 | 0,1487 | 0,6947 | 0,0049 |
|  | **Nonadecenoic acid** | 10 | 0 | 0,2263 | 8 | 0,6801 | 0,1506 | 0,6801 | 0,0329 |
|  | **Nonanoic acid** | 9 | 0 | 0,0969 | 8 | -0,1032 | 0,0882 | -0,1032 | 0,7368 |
|  | **Oleic acid;Elaidic acid** | 10 | 0 | 0,1193 | 8 | 0,5397 | 0,1141 | 0,5397 | 0,0049 |
|  | **Palmitic acid** | 10 | 0 | 0,1333 | 8 | 0,2310 | 0,1338 | 0,2310 | 0,2095 |
|  | **Palmitoleic acid** | 10 | 0 | 0,1261 | 8 | 0,3414 | 0,1244 | 0,3414 | 0,0732 |
|  | **Pentadecanoic acid** | 10 | 0 | 0,1873 | 8 | 0,3513 | 0,1391 | 0,3513 | 0,1443 |
|  | **Pentadecenoic acid** | 10 | 0 | 0,1437 | 8 | 0,2239 | 0,0978 | 0,2239 | 0,2258 |
|  | **Stearic acid** | 10 | 0 | 0,0984 | 8 | 0,1929 | 0,1137 | 0,1929 | 0,1856 |
|  | **Stearidonic acid** | 10 | 0 | 0,2421 | 8 | 0,0323 | 0,1643 | 0,0323 | 0,9128 |
|  | **Tetracosenoic acid** | 10 | 0 | 0,2515 | 8 | 0,4198 | 0,1683 | 0,4198 | 0,1761 |
|  | **Tetradecadienoic acid** | 4 | 0 | 0,4316 | 7 | 1,6622 | 0,2152 | 1,6622 | 0,0081 |
|  | **Tetradecanedioic acid** | 10 | 0 | 0,0841 | 8 | 0,2585 | 0,1185 | 0,2585 | 0,1033 |
|  | **Undecanedioic acid** | 10 | 3,05311E-17 | 0,0674 | 8 | 0,0876 | 0,0779 | 0,0876 | 0,3980 |
|  | **Undecanoic acid** | 10 | 0 | 0,0713 | 8 | -0,1163 | 0,0811 | -0,1163 | 0,3243 |
| **Indoles** | **Indole-3-propionic acid** | 9 | 0 | 0,1855 | 8 | -0,9695 | 0,6776 | -0,9695 | 0,0120 |
|  | **Tryptamine** | 9 | -2,46716E-17 | 0,1205 | 8 | 0,2826 | 0,1054 | 0,2826 | 0,1750 |
|  | **Tryptophan** | 10 | 0 | 0,1819 | 8 | 0,2846 | 0,1305 | 0,2846 | 0,2532 |
| **Lipids** | **Glycerol-3-phosphate** | 10 | 2,77556E-17 | 0,0942 | 8 | 0,3070 | 0,1796 | 0,3070 | 0,1205 |
|  | **Glycerophosphorylcholine** | 10 | 0 | 0,1074 | 8 | 0,5864 | 0,1312 | 0,5864 | 0,0017 |
|  | **PCae(12:0)** | 10 | 5,55112E-17 | 0,2548 | 8 | 0,5216 | 0,1854 | 0,5216 | 0,1318 |
|  | **PCae(14:0)** | 10 | 0 | 0,1192 | 8 | 0,5210 | 0,1014 | 0,5210 | 0,0081 |
|  | **PCae(15:0)** | 10 | 0 | 0,1817 | 8 | 0,5254 | 0,1159 | 0,5254 | 0,0457 |
|  | **PCae(16:0)** | 10 | 0 | 0,0790 | 8 | 0,2770 | 0,0913 | 0,2770 | 0,0312 |
|  | **PCae(16:1)** | 10 | 0 | 0,1399 | 8 | 0,5387 | 0,1047 | 0,5387 | 0,0134 |
|  | **PCae(17:0)** | 10 | 0 | 0,1327 | 8 | 0,6669 | 0,1126 | 0,6669 | 0,0041 |
|  | **PCae(18:0)** | 10 | 0 | 0,0832 | 8 | 0,6035 | 0,1263 | 0,6035 | 0,0010 |
|  | **PCae(18:1)** | 10 | 0 | 0,1076 | 8 | 0,5224 | 0,1260 | 0,5224 | 0,0033 |
|  | **PCae(18:2)** | 10 | 0 | 0,1069 | 8 | 0,3818 | 0,0708 | 0,3818 | 0,0148 |
|  | **PCae(18:3)** | 10 | 0 | 0,1191 | 8 | 0,4891 | 0,0671 | 0,4891 | 0,0077 |
|  | **PCae(20:0)** | 10 | -9,99201E-17 | 0,1367 | 8 | 0,1812 | 0,1256 | 0,1812 | 0,3830 |
|  | **PCae(20:1)** | 10 | 0 | 0,1283 | 8 | 0,5731 | 0,1415 | 0,5731 | 0,0054 |
|  | **PCae(20:2)** | 10 | 0 | 0,1473 | 8 | 0,7185 | 0,1849 | 0,7185 | 0,0026 |
|  | **PCae(20:3)** | 10 | 0 | 0,1173 | 8 | 0,8010 | 0,1979 | 0,8010 | 0,0003 |
|  | **PCae(20:4)** | 10 | 0 | 0,0698 | 8 | 0,9931 | 0,1603 | 0,9931 | 0,0000 |
|  | **PCae(22:0)** | 10 | 1,11022E-16 | 0,2006 | 8 | 0,2916 | 0,1591 | 0,2916 | 0,2851 |
|  | **PCae(22:1)** | 10 | 0 | 0,1704 | 8 | 0,4412 | 0,1463 | 0,4412 | 0,0733 |
|  | **PCae(22:2)** | 9 | 0 | 0,5662 | 8 | 1,6461 | 0,5304 | 1,6461 | 0,0191 |
|  | **PCae(22:3)** | 10 | 0 | 0,4274 | 8 | 0,9582 | 0,4347 | 0,9582 | 0,2007 |
|  | **PCae(22:4)** | 10 | 0 | 0,1098 | 8 | 1,1313 | 0,2101 | 1,1313 | 0,0000 |
|  | **PCae(22:6)** | 10 | 0 | 0,0764 | 8 | 0,7225 | 0,1164 | 0,7225 | 0,0000 |
|  | **PCee(16:0)** | 10 | 0 | 0,1026 | 8 | 0,5034 | 0,1069 | 0,5034 | 0,0039 |
|  | **Sphingosine-1-phosphate** | 10 | 0 | 0,0810 | 8 | 0,3877 | 0,1043 | 0,3877 | 0,0111 |
| **Nucleosides** | **Adenosine;2-deoxyguanosine** | 10 | -1,55431E-16 | 0,1464 | 8 | 0,3449 | 0,1312 | 0,3449 | 0,0981 |
|  | **Cytidine** | 10 | 4,44089E-17 | 0,1056 | 8 | 0,3735 | 0,1047 | 0,3735 | 0,0355 |
|  | **Inosine** | 10 | 0 | 0,0737 | 8 | 0,0758 | 0,0770 | 0,0758 | 0,4455 |
|  | **S-adenosylhomocysteine** | 10 | 0 | 0,1748 | 8 | 0,4154 | 0,2190 | 0,4154 | 0,0883 |
|  | **S-adenosylmethionine** | 10 | 0 | 0,1564 | 8 | 0,0174 | 0,2597 | 0,0174 | 0,9412 |
|  | **Succinyladenosine** | 10 | 0 | 0,1641 | 8 | 0,4828 | 0,2928 | 0,4828 | 0,0507 |
|  | **Uridine** | 10 | 0 | 0,0905 | 8 | 0,3505 | 0,1740 | 0,3505 | 0,0080 |
| **Organic acids** | **2-hydroxybutyric acid** | 10 | 1,66533E-17 | 0,0593 | 8 | -0,1329 | 0,0589 | -0,1329 | 0,2007 |
|  | **2-hydroxyglutaric acid** | 10 | 0 | 0,0716 | 8 | 0,3295 | 0,1164 | 0,3295 | 0,0116 |
|  | **2-oxoglutaric acid** | 10 | 0 | 0,1036 | 8 | 0,1447 | 0,1611 | 0,1447 | 0,4070 |
|  | **3-hydroxybutyric acid** | 10 | 1,38778E-17 | 0,0497 | 8 | 0,1354 | 0,0513 | 0,1354 | 0,0769 |
|  | **3-hydroxyglutaric acid** | 10 | 0 | 0,7032 | 8 | 0,7555 | 0,6988 | 0,7555 | 0,4648 |
|  | **Allantoin** | 10 | 0 | 0,0876 | 8 | 0,3220 | 0,0450 | 0,3220 | 0,0120 |
|  | **Azelaic acid** | 9 | 0 | 0,2156 | 8 | -0,8596 | 0,2502 | -0,8596 | 0,3141 |
|  | **Carnitine** | 10 | 0 | 0,0770 | 8 | -0,1350 | 0,0601 | -0,1350 | 0,3066 |
|  | **Carnitine C14:1(OH)** | 10 | 0 | 0,2228 | 8 | 1,3407 | 0,1870 | 1,3407 | 0,0010 |
|  | **Citric acid;Isocitric acid** | 10 | 4,44089E-17 | 0,0734 | 8 | 0,3708 | 0,0773 | 0,3708 | 0,0107 |
|  | **Fumaric acid** | 10 | 0 | 0,1017 | 8 | 0,1751 | 0,1964 | 0,1751 | 0,3224 |
|  | **Glycolic acid** | 10 | -2,66454E-16 | 0,0393 | 8 | -1,3034 | 0,0446 | -1,3034 | 0,0000 |
|  | **Hypotaurine** | 10 | 0 | 0,0692 | 8 | -0,0318 | 0,1138 | -0,0318 | 0,8774 |
|  | **Ketoisocaproic acid** | 10 | 0 | 0,1462 | 8 | -0,0633 | 0,1611 | -0,0633 | 0,8115 |
|  | **Ketoisovaleric acid** | 10 | 0 | 0,0948 | 8 | -0,0892 | 0,1300 | -0,0892 | 0,6449 |
|  | **Lactic acid** | 10 | 0 | 0,0424 | 8 | 0,1565 | 0,0257 | 0,1565 | 0,0107 |
|  | **Malic acid** | 10 | 0 | 0,1055 | 8 | 0,2276 | 0,1054 | 0,2276 | 0,2085 |
|  | **Methylmalonic acid** | 10 | -4,44089E-17 | 0,0918 | 8 | 0,3134 | 0,4040 | 0,3134 | 0,0389 |
|  | **N-acetylputrescine** | 10 | 0 | 0,1128 | 8 | -0,5295 | 0,1525 | -0,5295 | 0,0075 |
|  | **O-phosphoethanolamine** | 10 | 0 | 0,1484 | 8 | 0,4578 | 0,1909 | 0,4578 | 0,0657 |
|  | **Phosphoenolpyruvic acid** | 10 | 0 | 0,2209 | 8 | 0,1204 | 0,2692 | 0,1204 | 0,6543 |
|  | **Pyruvic acid;Oxaloacetic acid** | 10 | -2,77556E-17 | 0,1108 | 8 | 0,2971 | 0,0651 | 0,2971 | 0,0586 |
|  | **Succinic acid** | 10 | 5,55112E-17 | 0,0852 | 8 | 0,1811 | 0,3761 | 0,1811 | 0,1412 |
|  | **Taurine** | 10 | -3,33067E-17 | 0,0509 | 8 | 0,6842 | 0,0642 | 0,6842 | 0,0000 |
|  | **Urea** | 10 | 3,60822E-17 | 0,0440 | 8 | 0,2707 | 0,0745 | 0,2707 | 0,0068 |
| **Peptides** | **Glycylglycine** | 10 | -3,88578E-17 | 0,0810 | 8 | 0,1956 | 0,1550 | 0,1956 | 0,0901 |
|  | **NAAG** | 10 | -2,22045E-16 | 0,3645 | 8 | 0,2715 | 0,1989 | 0,2715 | 0,5456 |
|  | **Ox, glutathione** | 10 | 0 | 0,2362 | 8 | 0,6533 | 0,3458 | 0,6533 | 0,0520 |
|  | **S-lactoylglutathione** | 10 | 0 | 0,3442 | 8 | 0,7977 | 0,3361 | 0,7977 | 0,0930 |
| **Unclassified** | **3-phenylpropionic acid** | 9 | 0 | 0,2550 | 7 | -0,4040 | 0,2790 | -0,4040 | 0,3182 |
|  | **3-methylglutarylcarnitine*** | 10 | -5,55112E-17 | 0,1387 | 8 | -0,0669 | 0,0985 | -0,0669 | 0,7280 |
|  | **Desaminotyrosine*** | 10 | -4,71845E-17 | 0,3016 | 8 | -0,5016 | 0,1306 | -0,5016 | 0,1764 |
|  | **Hexanoylglycine*** | 10 | 0 | 0,3300 | 8 | 0,8800 | 0,6756 | 0,8800 | 0,0591 |
|  | **Hydroxydodecanoic acid*** | 10 | 0 | 0,0418 | 8 | 0,0433 | 0,0732 | 0,0433 | 0,5521 |
|  | **Mandelic acid*** | 10 | -1,66533E-17 | 0,0894 | 8 | 0,1523 | 0,0369 | 0,1523 | 0,3175 |
|  | **Methylsuccinic acid*** | 10 | -7,21645E-17 | 0,1368 | 8 | 0,0876 | 0,2622 | 0,0876 | 0,7071 |
|  | **Orotic acid*** | 10 | -1,22125E-16 | 0,8648 | 8 | 0,6635 | 0,5163 | 0,6635 | 0,5949 |
|  | **Alpha-tocopherol** | 10 | -0,0655 | 0,0914 | 8 | 0,0928 | 0,0734 | 0,1583 | 0,2461 |
|  | **Acetylcholine** | 10 | -1,11022E-16 | 0,1233 | 8 | 0,3738 | 0,0642 | 0,3738 | 0,0353 |
|  | **Anthranilic acid** | 10 | 2,22045E-17 | 0,1328 | 8 | -0,3494 | 0,2371 | -0,3494 | 0,0656 |
|  | **Ascorbic acid** | 9 | 0 | 0,3902 | 7 | 0,6027 | 0,4566 | 0,6027 | 0,3512 |
|  | **Hippuric acid** | 10 | 0 | 0,2609 | 8 | 0,3666 | 0,1665 | 0,3666 | 0,3865 |
|  | **Homovanillic acid** | 9 | -3,45403E-16 | 0,9992 | 7 | 1,6980 | 0,7189 | 1,6980 | 0,1852 |
|  | **Inositol** | 10 | -4,44089E-17 | 0,0554 | 8 | 0,3843 | 0,0501 | 0,3843 | 0,0033 |
|  | **Kynurenic acid** | 9 | 0 | 0,2726 | 8 | 0,1629 | 0,3061 | 0,1629 | 0,6627 |
|  | **Kynurenine** | 10 | -2,77556E-17 | 0,0944 | 8 | 0,7090 | 0,1710 | 0,7090 | 0,0015 |
|  | **Niacinamide** | 10 | 0 | 0,1136 | 8 | -0,1536 | 0,1576 | -0,1536 | 0,4304 |
|  | **Nicotinic acid** | 10 | 3,33067E-17 | 0,0573 | 8 | 0,1110 | 0,0623 | 0,1110 | 0,4282 |
|  | **Phosphoric acid** | 10 | 0 | 0,0469 | 8 | 0,0648 | 0,0377 | 0,0648 | 0,3087 |
|  | **Putrescine** | 10 | 0 | 0,1480 | 8 | 0,1742 | 0,2126 | 0,1742 | 0,4239 |
|  | **Shikimic acid** | 10 | -3,88578E-17 | 0,1341 | 8 | 0,3126 | 0,1257 | 0,3126 | 0,1265 |
| ***Ratios*** | ***AcCarn/Carn*** | *10* | *0* | *0,0836* | *8* | *0,4297* | *0,0333* | *0,4297* | *0,0083* |
|  | ***AcPut/Put*** | *10* | *-3,33067E-17* | *0,1967* | *8* | *-0,7147* | *0,2691* | *-0,7147* | *0,0087* |
|  | ***aHGA/aKG*** | *10* | *0* | *0,0449* | *8* | *0,1736* | *0,0661* | *0,1736* | *0,0670* |
|  | ***aKG/Glu*** | *10* | *0* | *0,0646* | *8* | *-0,3193* | *0,2526* | *-0,3193* | *0,0191* |
|  | ***Anthr/Kyn*** | *10* | *0* | *0,1771* | *8* | *-1,0580* | *0,2186* | *-1,0580* | *0,0011* |
|  | ***Asn/Asp*** | *10* | *0* | *0,1347* | *8* | *-0,1129* | *0,2341* | *-0,1129* | *0,5206* |
|  | ***Asp/Malate*** | *10* | *3,60822E-17* | *0,0892* | *8* | *0,2350* | *0,1837* | *0,2350* | *0,0984* |
|  | ***Asp/NAcAsp*** | *10* | *0* | *0,5782* | *8* | *0,2328* | *0,9215* | *0,2328* | *0,7770* |
|  | ***BetaAla/Asp*** | *10* | *-8,88178E-17* | *0,1291* | *8* | *1,3647* | *0,2226* | *1,3647* | *0,0000* |
|  | ***Cit/Arg*** | *10* | *0* | *0,2740* | *8* | *0,7801* | *0,3290* | *0,7801* | *0,0719* |
|  | ***Cit/Orn*** | *10* | *-5,55112E-18* | *0,0573* | *8* | *0,6423* | *0,0686* | *0,6423* | *0,0000* |
|  | ***Citrate/Oaa*** | *10* | *0* | *0,1114* | *8* | *0,0855* | *0,1161* | *0,0855* | *0,5421* |
|  | ***DeOGuanosine/Guanine*** | *10* | *0* | *0,1987* | *8* | *0,0513* | *0,4187* | *0,0513* | *0,8498* |
|  | ***DMGly/Betaine*** | *10* | *-3,81639E-18* | *0,1218* | *8* | *-0,1317* | *0,0431* | *-0,1317* | *0,3994* |
|  | ***Fum/Malate*** | *10* | *-1,94289E-17* | *0,0381* | *8* | *-0,0683* | *0,1238* | *-0,0683* | *0,2707* |
|  | ***Fum/Suc*** | *10* | *0* | *0,0779* | *8* | *-0,0270* | *0,2776* | *-0,0270* | *0,8763* |
|  | ***GABA/Glu*** | *10* | *0* | *0,1049* | *8* | *-0,5901* | *0,1852* | *-0,5901* | *0,0113* |
|  | ***Glc6P/Glc*** | *10* | *3,33067E-17* | *0,2276* | *8* | *0,5206* | *0,3221* | *0,5206* | *0,1039* |
|  | ***Glu/Gln*** | *10* | *0* | *0,1500* | *8* | *0,1302* | *0,1442* | *0,1302* | *0,5064* |
|  | ***Glu/Oxoproline*** | *10* | *1,94289E-17* | *0,0590* | *8* | *0,3168* | *0,1375* | *0,3168* | *0,0017* |
|  | ***Glyc3P/Glycerol*** | *10* | *0* | *0,2610* | *8* | *0,3298* | *0,3651* | *0,3298* | *0,3926* |
|  | ***Glyc3P/GPCho*** | *10* | *0* | *0,1586* | *8* | *-0,3285* | *0,2263* | *-0,3285* | *0,1624* |
|  | ***HOPro/Pro*** | *10* | *0* | *0,1136* | *8* | *0,6244* | *0,0766* | *0,6244* | *0,0034* |
|  | ***HXanthine/Inosine*** | *10* | *1,9984E-16* | *0,0798* | *8* | *2,7685* | *0,7381* | *2,7685* | *0,0038* |
|  | ***Inosine/Adenosine*** | *10* | *0* | *0,2119* | *8* | *-0,2169* | *0,1472* | *-0,2169* | *0,4155* |
|  | ***KIC/Leu*** | *10* | *0* | *0,1263* | *8* | *-0,1608* | *0,1162* | *-0,1608* | *0,5070* |
|  | ***Kyn/Trp*** | *10* | *0* | *0,1175* | *8* | *0,4128* | *0,0972* | *0,4128* | *0,0408* |
|  | ***NAAG/NAcAsp*** | *10* | *0* | *0,5906* | *8* | *-0,0196* | *0,7540* | *-0,0196* | *0,9810* |
|  | ***Oaa/Asp*** | *10* | *0* | *0,0798* | *8* | *-0,1724* | *0,1977* | *-0,1724* | *0,1011* |
|  | ***Oaa/Malate*** | *10* | *4,44089E-17* | *0,0875* | *8* | *0,0626* | *0,1171* | *0,0626* | *0,6210* |
|  | ***OPEt/S1P*** | *10* | *0* | *0,3079* | *8* | *0,2471* | *0,3630* | *0,2471* | *0,6019* |
|  | ***Orn/Arg*** | *10* | *0* | *0,2594* | *8* | *0,1378* | *0,2930* | *0,1378* | *0,7103* |
|  | ***PCr/Creatine*** | *10* | *0* | *0,2591* | *8* | *0,4194* | *0,3324* | *0,4194* | *0,2873* |
|  | ***PEP/Oaa*** | *10* | *0* | *0,1896* | *8* | *-0,1708* | *0,3088* | *-0,1708* | *0,4535* |
|  | ***Put/Orn*** | *10* | *0* | *0,1609* | *8* | *-0,0016* | *0,2580* | *-0,0016* | *0,9936* |
|  | ***Pyr/Ala*** | *10* | *1,11022E-17* | *0,0908* | *8* | *0,4840* | *0,0808* | *0,4840* | *0,0015* |
|  | ***Pyr/Lac*** | *10* | *0* | *0,0735* | *8* | *0,1152* | *0,0435* | *0,1152* | *0,2327* |
|  | ***Pyr/Malate*** | *10* | *4,44089E-17* | *0,0875* | *8* | *0,0626* | *0,1171* | *0,0626* | *0,6210* |
|  | ***Pyr/PEP*** | *10* | *0* | *0,1896* | *8* | *0,1708* | *0,3088* | *0,1708* | *0,4535* |
|  | ***Pyr/Ser*** | *10* | *0* | *0,0912* | *8* | *0,0934* | *0,1678* | *0,0934* | *0,5523* |
|  | ***Rib5P/Ribose*** | *10* | *-4,44089E-17* | *0,1143* | *8* | *0,2498* | *0,2101* | *0,2498* | *0,5249* |
|  | ***SAM/Met*** | *10* | *0* | *0,1476* | *8* | *0,2343* | *0,2411* | *0,2343* | *0,3252* |
|  | ***SAM/SAH*** | *10* | *0* | *0,1338* | *8* | *-0,3685* | *0,1478* | *-0,3685* | *0,0372* |
|  | ***Ser/Gly*** | *10* | *0* | *0,0989* | *8* | *0,1286* | *0,1205* | *0,1286* | *0,3691* |
|  | ***Stearate/Linolenate*** | *10* | *0* | *0,0674* | *8* | *-0,5034* | *0,0590* | *-0,5034* | *0,0000* |
|  | ***Sucrose/Raffinose*** | *10* | *0* | *0,7006* | *8* | *-0,3574* | *0,1742* | *-0,3574* | *0,7663* |
|  | ***Tyr/Phe*** | *10* | *0* | *0,1000* | *8* | *0,0150* | *0,0833* | *0,0150* | *0,9178* |
|  | ***Uracil/Uridine*** | *10* | *0* | *0,2141* | *8* | *0,4075* | *0,2627* | *0,4075* | *0,2197* |
|  | ***Urate/Xanthine*** | *10* | *-1,77636E-16* | *0,8913* | *8* | *-5,2875* | *1,1962* | *-5,2875* | *0,0079* |
|  | ***Urea/Arginine*** | *10* | *0* | *0,2379* | *8* | *0,2617* | *0,2905* | *0,2617* | *0,4741* |
|  | ***Val/KIV*** | *10* | *0* | *0,0855* | *8* | *0,0598* | *0,0986* | *0,0598* | *0,7876* |
|  | ***Xanthine/Guanine*** | *10* | *-1,33227E-16* | *0,8878* | *8* | *5,3932* | *1,1998* | *5,3932* | *0,0059* |
|  | ***Xanthine/HXanthine*** | *10* | *1,88738E-16* | *0,8820* | *8* | *2,7903* | *0,8362* | *2,7903* | *0,0409* |
|  | ***Xylose/Xylitol*** | *10* | *0* | *0,2005* | *8* | *-1,0194* | *0,1721* | *-1,0194* | *0,0007* |
